# The clinical heterogeneity of coenzyme Q_10_ deficiency results from genotypic differences in the *Coq9* gene

**DOI:** 10.15252/emmm.201404632

**Published:** 2015-03-23

**Authors:** Marta Luna-Sánchez, Elena Díaz-Casado, Emanuele Barca, Miguel Ángel Tejada, Ángeles Montilla-García, Enrique Javier Cobos, Germaine Escames, Dario Acuña-Castroviejo, Catarina M Quinzii, Luis Carlos López

**Affiliations:** 1 Departamento de Fisiología, Facultad de Medicina, Universidad de GranadaGranada, Spain; 2 Centro de Investigación Biomédica, Instituto de Biotecnología, Parque Tecnológico de Ciencias de la SaludGranada, Spain; 3 Department of Neurology, Columbia University Medical CenterNew York, NY, USA; 4 Departamento de Farmacología, Facultad de Medicina, Universidad de GranadaGranada, Spain; 5 Centro de Investigación Biomédica, Instituto de Neurociencias, Parque Tecnológico de Ciencias de la SaludGranada, Spain

**Keywords:** CoQ multiprotein complex, *Coq9*, mitochondrial myopathy, mouse model, nonsense-mediated mRNA decay

## Abstract

Primary coenzyme Q_10_ (CoQ_10_) deficiency is due to mutations in genes involved in CoQ biosynthesis. The disease has been associated with five major phenotypes, but a genotype–phenotype correlation is unclear. Here, we compare two mouse models with a genetic modification in *Coq9* gene (*Coq9*^*Q95X*^ and *Coq9*^*R239X*^), and their responses to 2,4-dihydroxybenzoic acid (2,4-diHB). *Coq9*^*R239X*^ mice manifest severe widespread CoQ deficiency associated with fatal encephalomyopathy and respond to 2,4-diHB increasing CoQ levels. In contrast, *Coq9*^*Q95X*^ mice exhibit mild CoQ deficiency manifesting with reduction in CI+III activity and mitochondrial respiration in skeletal muscle, and late-onset mild mitochondrial myopathy, which does not respond to 2,4-diHB. We show that these differences are due to the levels of COQ biosynthetic proteins, suggesting that the presence of a truncated version of COQ9 protein in *Coq9*^*R239X*^ mice destabilizes the CoQ multiprotein complex. Our study points out the importance of the multiprotein complex for CoQ biosynthesis in mammals, which may provide new insights to understand the genotype–phenotype heterogeneity associated with human CoQ deficiency and may have a potential impact on the treatment of this mitochondrial disorder.

## Introduction

Coenzyme Q (CoQ) is an essential molecule for mitochondrial ATP synthesis and other metabolic processes (Turunen *et al*, 2004; Garcia-Corzo *et al*, 2013). Its endogenous biosynthesis occurs ubiquitously in the mitochondria and starts with the formation of a 4-hydroxybenzoate (4-HB) head group and a lipophilic polyisoprenoid tail. While the quinone ring is derived from tyrosine or phenylalanine, the isoprenoid side chain is produced by addition of isopentenyl diphosphate molecules to farnesyl diphosphate or geranylgeranyl diphosphate in multiple steps catalyzed by polyprenyl diphosphate synthase (PDSS1–PDSS2). Then, 4-para-hydroxybenzoate:polyprenyl transferase, encoded by *Coq2*, mediates the conjugation of the aromatic ring precursor, 4-HB, to the side chain, while five other enzymes, encoded by *Coq3* to *Coq7*, reside in the mitochondrial inner membrane and modify the quinone ring of CoQ (Supplementary Fig S1) (Tran & Clarke, 2007). Other proteins are thought to have regulatory functions in the CoQ biosynthetic pathway: (i) COQ9 is essential for the function of COQ7, an enzyme that catalyzes the hydroxylation of demethoxyubiquinone to produce 5-hydroxyquinone (Garcia-Corzo *et al*, 2013); (ii) ADCK3 and ADCK4 regulate other CoQ biosynthetic proteins by their kinase activities (Tran & Clarke, 2007); and (iii) PTC7 regulates the activity of COQ7 by its phosphatase activity (Martin-Montalvo *et al*, 2013). Moreover, several studies have shown evidence that, in yeast, the enzymes required for CoQ biosynthesis are organized in a multiprotein complex. This organization would allow channeling of labile/reactive intermediates, enhance catalytic efficiency, and provide a mechanism for coordinative regulation of components (Tran & Clarke, 2007). However, there is no proof of the existence of a multiprotein complex for CoQ biosynthesis in mammals.

Mutations in CoQ biosynthetic genes produce primary CoQ_10_ deficiency, a mitochondrial syndrome with five major clinical presentations: (i) encephalomyopathy with brain involvement and recurrent myoglobinuria; (ii) infantile multisystem disorder with encephalopathy usually associated with nephropathy and variable involvement of other organs; (iii) ataxic syndrome with cerebellar atrophy; (iv) isolated myopathy; and (v) steroid-resistant nephrotic syndrome (Emmanuele *et al*, 2012). Moreover, mutations in *COQ2* have been recently reported in Japanese patients with multiple system atrophy (Multiple-System Atrophy Research, 2013). The causes of this clinical variability are unknown, and it is difficult to explain why mutations in the same gene may cause different phenotypes, for example, mutations in *COQ2* and *COQ6* have been associated with isolated nephropathy or multisystemic disease (Quinzii *et al*, 2006; Diomedi-Camassei *et al*, 2007; Heeringa *et al*, 2011; Jakobs *et al*, 2013), due to the limited number of patients described.

To better understand the pathophysiologic consequences of primary CoQ_10_ deficiency, we recently generated a mouse model carrying a homozygous mutation in *Coq9* gene (R239X, *Coq9*^*R239X*^). This mutation is homologue to the human R244X mutation (Duncan *et al*, 2009). *Coq9*^*R239X*^ mice showed widespread CoQ deficiency (Garcia-Corzo *et al*, 2013), and their characterization demonstrated that: (i) the presence of a dysfunctional COQ9 protein and/or the deficit in CoQ in the brain causes an increase in free complex III, leading to a decrease in mitochondrial respiration and ATP synthesis, (ii) mitochondrial dysfunction in the brain induces oxidative damage and a caspase-independent apoptotic cell death, and (iii) the encephalomyopathic form of CoQ deficiency is progressive and takes place with neuronal death, severe reactive astrogliosis and spongiform degeneration. Therefore, *Coq9*^*R239X*^ mice show clinical, histopathological, biochemical and molecular signs of a fatal mitochondrial encephalomyopathy (Garcia-Corzo *et al*, 2013).

To understand the molecular mechanisms underlying the genotype–phenotype correlation in CoQ deficiency, we have generated and characterized at biochemical, molecular and clinical level a new mouse model with a different genetic defect in the same *Coq9* gene, specifically, a homozygous *Coq9* Q95X mutant (*Coq9*^*Q95X*^*),* to compare with the *Coq9*^*R239X*^ mouse model.

## Results

### Lack of the Coq9 protein causes moderate CoQ deficiency

**Figure 1:**
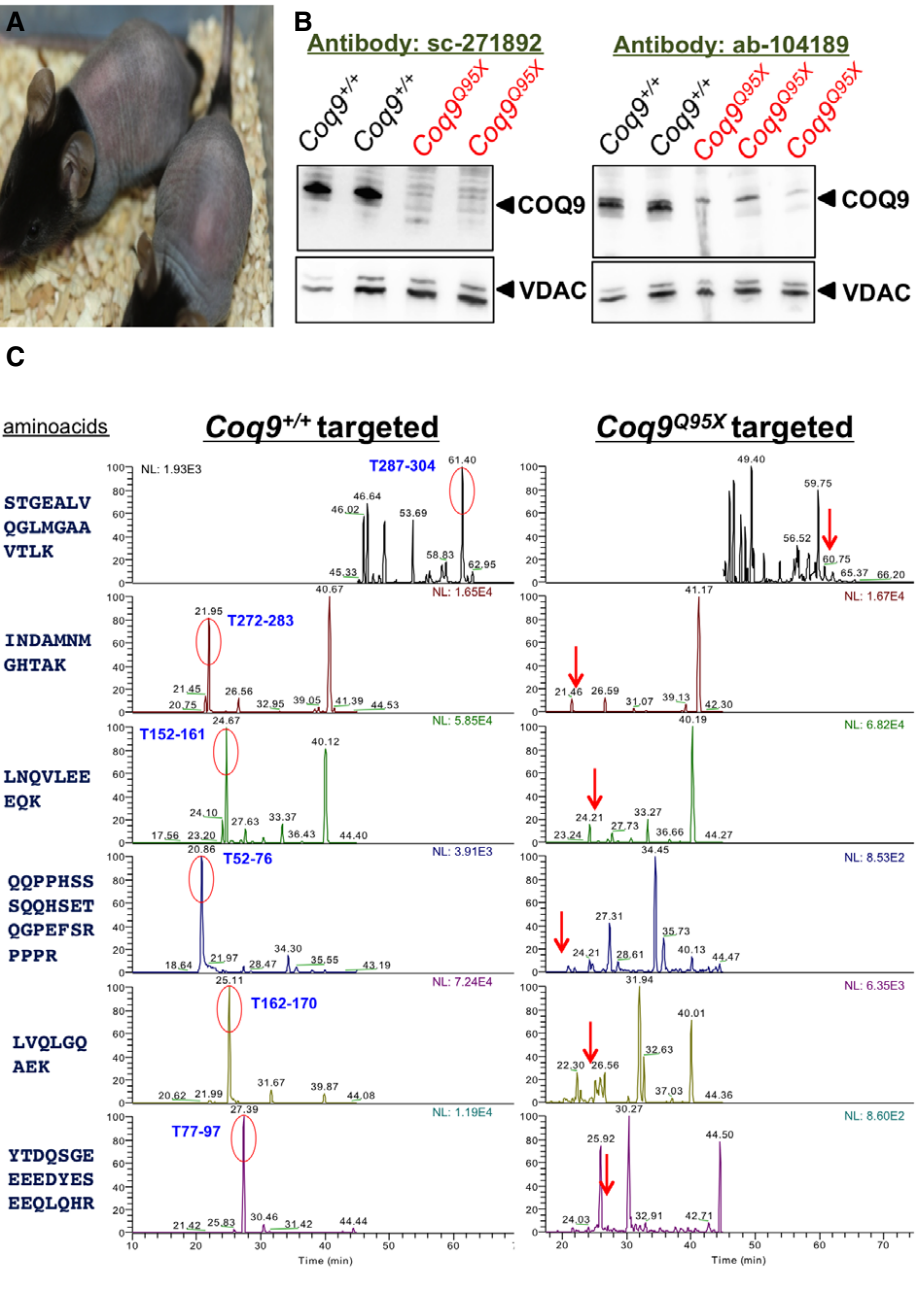
*Coq9*^*Q95X*^ mice at 21 postnatal days and analysis of COQ9 protein

*Coq9*^*Q95X*^ mice pups had normal development and were indistinguishable from wild-type mice (*Coq9*^+/+^). As described in the *Coq9*^*R239X*^, by postnatal day 21, *Coq9*^*Q95X*^ mice had also lost their body hair (Fig1A), which grew back during the next hair growth cycle.

*Coq9*^*Q95X*^ mice at 21 postnatal days showing the loss of corporal hair.

Representative Western blot images of COQ9 protein in kidney homogenate from *Coq9*^+/+^ (*n* = 4) and *Coq9*^*Q95X*^ mice (*n* = 4) at 3 months of age. Antibody sc-271892 was used to map the C-terminal region of the COQ9 protein and antibody ab-104189 was used to map the internal sequence of the COQ9 protein.

High-resolution LC-MS/MS proteomic analysis of kidney mitochondria from *Coq9*^+/+^ (*n* = 3) and *Coq9*^*Q95X*^ mice (*n* = 3) at 3 months of age. None of the six peptides of the COQ9 protein identified in *Coq9*^+/+^ mice was detected in *Coq9*^*Q95X*^ mice. Source data are available online for this figure.

To prove that wild-type COQ9 protein was not produced in *Coq9*^*Q95X*^ mice, we first performed an immunoblotting analysis using two different anti-COQ9 antibodies: one against amino acids 165–318, to map the C-terminal region of the protein (sc-271892), and the other against amino acids 160–190, corresponding to a region within internal sequence of the COQ9 protein (ab-104189). No protein was detected in *Coq9*^*Q95X*^ mice compared to wild-type (Fig1B). To check whether the premature termination of the COQ9 protein induces the complete loss of the protein, we also performed a proteomic analysis by high-resolution LC-MS/MS against six identified peptides from the COQ9 protein, one of them being (QQPPHSSSQQHSETQGPEFSRPPR) present in a possible truncated version of the protein of 95 amino acids (Pagliarini *et al*, 2008). Although COQ9 was clearly detected in the wild-type samples, none of its peptides were observed in *Coq9*^*Q95X*^ mice (Fig1C), demonstrating that the COQ9 protein was completely absent in *Coq9*^*Q95X*^ mice. In contrast, in *Coq9*^*R239X*^ mice, a truncated version of COQ9 protein was observed by Western blot using an antibody against the internal sequence of the protein (Supplementary Fig S2).

**Figure 2:**
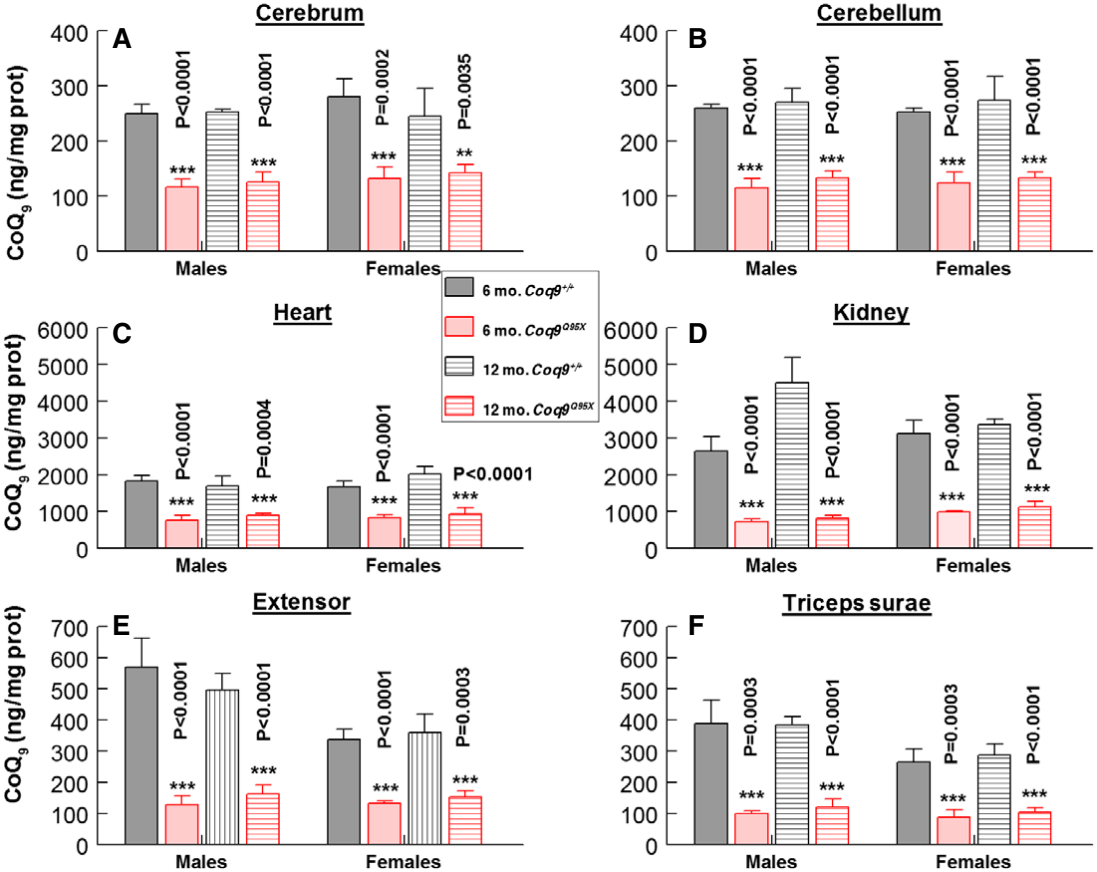
*Coq9*^*Q95X*^ mice showed moderate CoQ deficiency

The consequence of the lack of the COQ9 protein was a significant decrease of both CoQ_9_ (the major form of ubiquinone in rodents) and CoQ_10_ levels in all examined tissues (cerebrum, cerebellum, heart, kidney, extensor and *triceps surae*) of *Coq9*^*Q95X*^ mice compared with the age-mated *Coq9*^+/+^mice (Fig2A–F and Supplementary Fig S3A–F). While CoQ_9_ levels were around 50% in cerebrum, cerebellum and heart (Fig2A–C), kidney and skeletal muscle had 30% of residual CoQ_9_ levels compared with wild-type animals (Fig2D–F).

A–F CoQ_9_ levels in tissue homogenates from brain (A), cerebellum (B), heart (C), kidney (D), extensor (E) and *triceps surae* (F) of male and female *Coq9*^+/+^ and *Coq9*^*Q95X*^ mice at 6 and 12 months of age. Data are expressed as mean ± SD. Statistical analysis was performed on 6*-*month-old *Coq9*^+/+^ mice versus 6*-*month-old *Coq9*^*Q95X*^ mice and 12*-*month-old *Coq9*^+/+^ mice versus 12*-*month-old *Coq9*^*Q95X*^ mice. **P < 0.01; ***P < 0.001. Student's *t*-test (*n* = 8 for each group). Source data are available online for this figure.

**Figure 3:**
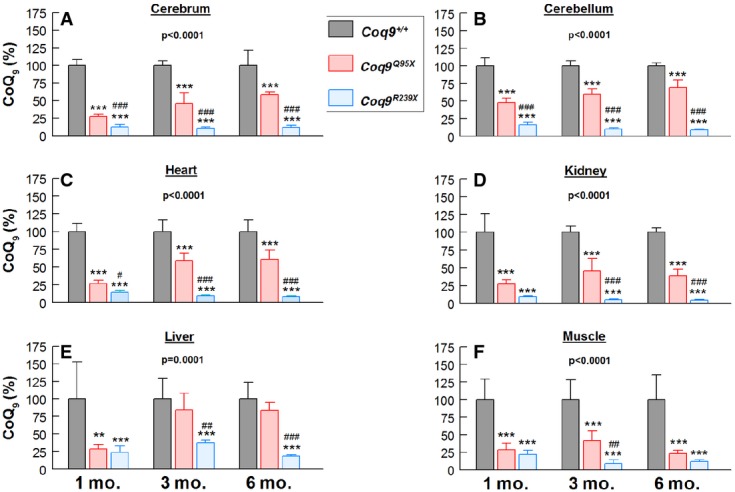
*Coq9*^*Q95X*^ mice exhibited higher CoQ levels compared with *Coq9*^*R239X*^ mice

An intriguing observation was that in all tissues, CoQ_9_ levels in *Coq9*^*Q95X*^ mice were higher compared with *Coq9*^*R239X*^ mice, in which residual CoQ_9_ levels were around 20% compared to wild-type animals (Fig3A–F). However, muscle was the tissue with more similar CoQ_9_ levels between both models (Fig3F).

A–F Residual CoQ_9_ levels in tissue homogenates from brain (A), cerebellum (B), heart (C), kidney (D), liver (E) and skeletal muscle (F) of *Coq9*^+/+^, *Coq9*^*Q95X*^ and *Coq9*^*R239X*^ mice at 1, 3 and 5 months of age. Data are expressed as mean ± SD. **P < 0.01; ***P < 0.001; *Coq9*^*Q95X*^ and *Coq9*^*R239X*^ mice versus *Coq9*^+/+^ mice. ^#^*P* < 0.05; ^##^*P* < 0.01; ^###^*P* < 0.001; *Coq9*^*Q95X*^ versus *Coq9*^*R239X*^ mice (one-way ANOVA with a Tukey's *post hoc* test; *n* = 8 for each group; numbers above columns indicate *P*-values of the one-way ANOVA test).

### Two distinctive mutations in the *Coq9* gene induce different changes in the Coq biosynthetic gene expression and protein level

The differences found in CoQ_9_ levels between the *Coq9*^*Q95X*^ and *Coq9*^*R239X*^ mice may be due to differences in the expression of the *Coq* genes or, alternatively, to differences in the stability of the CoQ multiprotein complex manifested by different levels of COQ proteins.

By virtue of that, we first determined mRNA expression levels of some genes encoding proteins that are involved in CoQ biosynthesis and are components of the CoQ mutiprotein complex, that is, *Coq9*, *Coq7*, *Coq6, Coq5* and *Adck3,* in cerebrum, kidney and muscle from age-matched *Coq9*^+/+^, *Coq9*^*Q95X*^ and *Coq9*^*R239X*^ mice.

**Figure 4:**
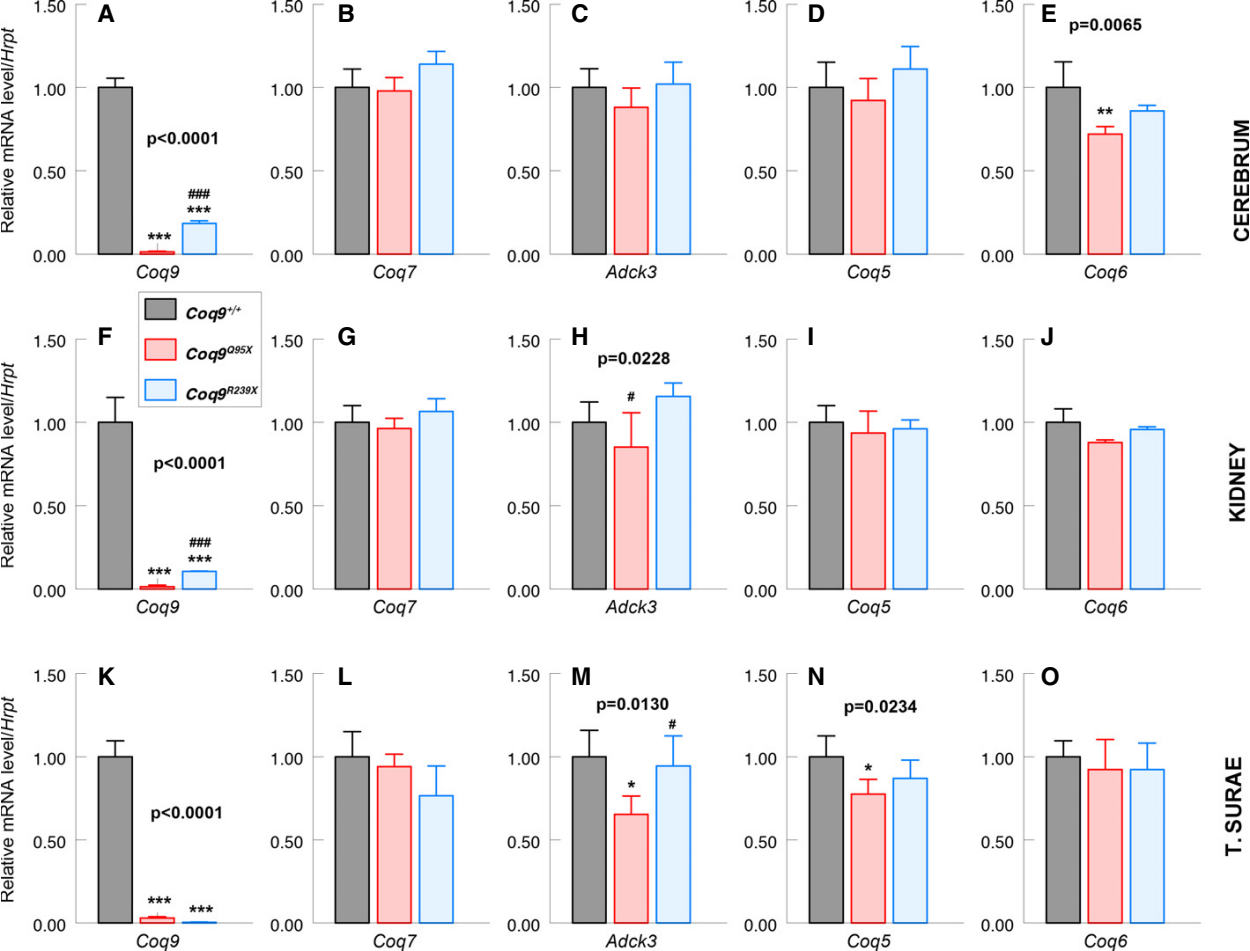
CoQ biosynthetic gene expression

In cerebrum and kidney, *Coq9* mRNA levels were nearly undetectable in *Coq9*^*Q95X*^ compared with *Coq9*^+/+^ mice (1 ± 0.38 and 1 ± 0.90%, respectively) and significantly decreased in *Coq9*^*R239X*^ compared with *Coq9*^+/+^ mice (18 ± 0.38 and 10 ± 0.21%, respectively) (Fig4A and F). Similar levels of *Coq7* and *Coq5* mRNA expression were detected in cerebrum and kidney of *Coq9*^*Q95X*^, *Coq9*^*R239X*^ and *Coq9*^+/+^ mice (Fig4B, D, G and I), while *Coq6* was significantly decreased (72.1 ± 4.35%) only in cerebrum of *Coq9*^*Q95X*^ compared with *Coq9*^+/+^ mice (Fig4D); *Adck3* was slightly increased in kidney of *Coq9*^*R239X*^ compared to Coq9^*Q95X*^ (116 ± 7.9 versus 85.1 ± 20.6%) (Fig4H). In muscle, *Coq9* mRNA levels were similarly decreased in both *Coq9*^*Q95X*^ and *Coq9*^*R239X*^ (3 ± 0.9 and 0.5 ± 0.2%) compared to *Coq9*^+/+^ mice (Fig4K). Moreover, *Adck3* and *Coq5* mRNA levels were significantly decreased in *Coq9*^*Q95X*^ mice compared to *Coq9*^+/+^ mice (65.3 ± 11.1% for *Adck3* and 77.6 ± 8.9% for *Coq5*) (Fig4M and N). Comparing the two mutant mice, it is remarkable that *Coq9* mRNA expression levels in cerebrum and kidney of *Coq9*^*R239X*^ mice were significantly higher compared to *Coq9*^*Q95X*^ (18.3 ± 1.6 versus 1.3 ± 0.4% in cerebrum and 10.6 ± 2.2 versus 1.3 ± 0.9% in kidney) (Fig4A and F). In contrast, in muscle, there were no differences in *Coq9* mRNA levels between the two mutant models (Fig4K). The degradation of the mutant *Coq9* mRNA in both mouse models (*Coq9*^*Q95X*^ and *Coq9*^*R239X*^) is due to nonsense-mediated mRNA decay (NMD) since the treatment of mutant MEFs with cyclohexamide, an inhibitor of NMD (Rio Frio *et al*, 2008), increased the levels of *Coq9* mRNA in *Coq9*^*Q95X*^ (fold increase 5.5 ± 1.1, treated/untreated) and *Coq9*^*R239X*^ (fold increase 21.4 ± 6.8, treated/untreated) compared to the mild effect in *Coq9*^+/+^ (fold increase 1.5 ± 0.1, treated/untreated) cells (Table1).

**Table 1:** Administration of cyclohexamide (CH) inhibits NMD in MEFs from *Coq9*^*Q95X*^ and *Coq9*^*R239X*^ mice

	*Coq9* mRNA (CH-treated/untreated)
*Coq9*^+/+^	1.54 ± 0.12
*Coq9*^*Q95X*^	5.47 ± 1.14*
*Coq9*^*R239X*^	21.44 ± 6.8**,##

The results are represented as fold increase of *Coq9* mRNA levels after cyclohexamide administration. Data are expressed as the mean ± SD of five experiments in triplicates per group. One-way ANOVA with a Tukey *post hoc* test.

* *P* < 0.05;

** *P* < 0.01; *Coq9^Q95X^* and *Coq9^R239X^* mice versus *Coq9*^+/+^ mice.

## *P* < 0.01; *Coq9^Q95X^* versus *Coq9^R239X^* mice. One-way ANOVA for comparison between the three experimental groups: *P* = 0.0022.

A–E mRNA expression levels of *Coq9* (A), *Coq7* (B), *Adck3* (C), *Coq5* (D) and *Coq6* (E) on cerebrum of *Coq9*^+/+^, *Coq9*^*Q95X*^ and *Coq9*^*Q95X*^ mice at 3 months of age. **P < 0.01; ***P < 0.001; *Coq9*^*Q95X*^ and *Coq9*^*R239X*^ mice versus *Coq9*^+/+^ mice. ^###^*P* < 0.001; *Coq9*^*Q95X*^ versus *Coq9*^*R239X*^ mice.

F–J mRNA expression levels of *Coq9* (F), *Coq7* (G), *Adck3* (H), *Coq5* (I) and *Coq6* (J) on kidney of *Coq9*^+/+^, *Coq9*^*Q95X*^ and *Coq9*^*Q95X*^ mice at 3 months of age. ***P < 0.001; *Coq9*^*Q95X*^ and *Coq9*^*R239X*^ mice versus *Coq9*^+/+^ mice. ^#^*P* < 0.05; ^###^*P* < 0.001; *Coq9*^*Q95X*^ versus *Coq9*^*R239X*^ mice.

K–O mRNA expression levels of *Coq9* (K), *Coq7* (L), *Adck3* (M), *Coq5* (N*)* and *Coq6* (O) on *triceps surae* of *Coq9*^+/+^, *Coq9*^*Q95X*^ and *Coq9*^*Q95X*^ mice at 3 months of age. *P < 0.05; ***P < 0.001; *Coq9*^*Q95X*^ and *Coq9*^*R239X*^ mice versus *Coq9*^+/+^ mice. ^#^*P* < 0.05; *Coq9*^*Q95X*^ versus *Coq9*^*R239X*^ mice. Data information: All values are presented as mean ± SD. One-way ANOVA with a Tukey's *post hoc* test. Numbers above columns indicate *P*-values of the one-way ANOVA test (*n* = 5 for each group).

**Figure 5:**
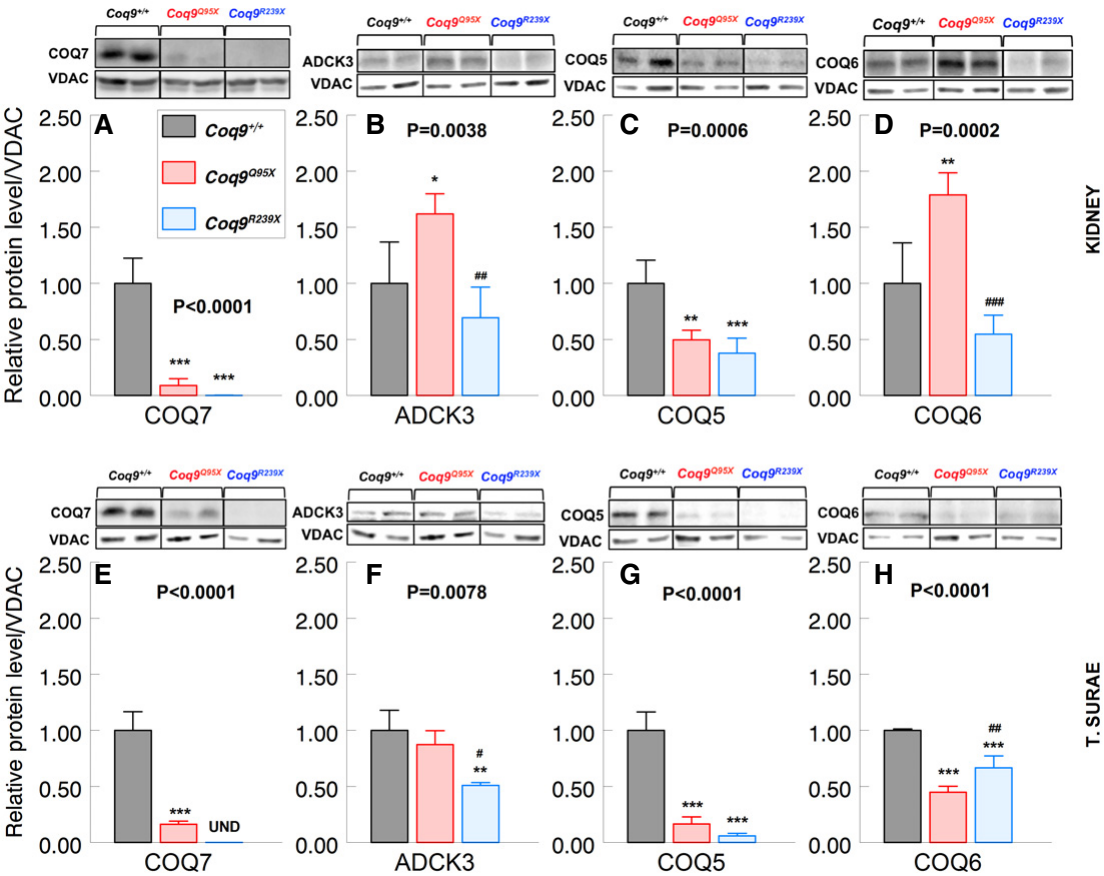
Levels of COQ biosynthetic proteins

Secondly, we measured the levels of the CoQ biosynthetic proteins encoded by these genes. In *Coq9*^*Q95X*^ mice, steady-state levels of COQ7 and COQ5 were significantly decreased in cerebrum (19 ± 9 and 41 ± 13%), kidney (9 ± 6 and 50 ± 9%) and muscle (16 ± 3 and 17 ± 6%) compared with *Coq9*^+/+^ mice. *Coq9*^*R239X*^ mice showed extremely reduced levels of COQ5 and COQ7 in cerebrum (0.1 ± 0.1 and 35 ± 11%), kidney (0.1 ± 0.1 and 38 ± 14%), and muscle (undetectable, and 17 ± 6%) compared to *Coq9*^+/+^ mice (Supplementary Fig S4A and C; Fig5A, C, E and G).

A–D Representative Western blot and quantitation of Western blot bands of COQ7 (A), ADCK3 (B), COQ5 (C) and COQ6 (D), and VDAC1 as a loading control in the kidneys of 3-month-old mice. *P < 0.05; **P < 0.01; ***P < 0.001; *Coq9*^*Q95X*^ and *Coq9*^*R239X*^ mice versus *Coq9*^+/+^ mice. ^##^*P* < 0.01; ^###^*P* < 0.001; *Coq9*^*Q95X*^ versus *Coq9*^*R239X*^ mice. One-way ANOVA with a Tukey's *post hoc* test.

E–H Representative Western blot and quantitation of Western blot bands of COQ7 (E), ADCK3 (F), COQ5 (G) and COQ6 (H), and VDAC1 as a loading control in skeletal muscle of 3-month-old mice. **P < 0.01; ***P < 0.001; *Coq9*^*Q95X*^ and *Coq9*^*R239X*^ mice versus *Coq9*^+/+^ mice. ^#^*P* < 0.05; ^##^*P* < 0.01; *Coq9*^*Q95X*^ versus *Coq9*^*R239X*^ mice. Data information: All values are presented as mean ± SD. One-way ANOVA with a Tukey's *post hoc* test. Numbers above columns indicate *P*-values of the one-way ANOVA test. *Coq9*^+/+^ mice *n* = 4; *Coq9*^*Q95X*^ and *Coq9*^*R239X*^ mice *n* = 5. Source data are available online for this figure.

In cerebrum and muscle, ADCK3 levels were unchanged in *Coq9*^*Q95X*^ mice and decreased in *Coq9*^*R239X*^ mice compared to *Coq9*^+/+^ mice (55 ± 19 and 51 ± 3%) (Supplementary Fig S4; Fig5F). In kidney, ADCK3 and COQ6 levels were significantly increased in *Coq9*^*Q95X*^ mice (162 ± 18 and 179 ± 20%) compared with *Coq9*^+/+^ mice and reduced in *Coq9*^*R239X*^ mice compared with *Coq9*^*Q95X*^ mice (43 ± 17% for ADCK3 and 31 ± 9% for COQ6) (Fig5B and D). Muscle of *Coq9*^*Q95X*^ mice also showed a significant decrease of COQ6 compared to *Coq9*^+/+^ mice (45 ± 5%) (Fig5H).

Consistent with the results obtained in *Coq9*^*R239X*^ mice, human skin fibroblasts carrying the R239X homologue mutation (*COQ9*^*R244X*^) showed a reduction in COQ9, COQ7, ADCK3 and COQ5 protein levels (Supplementary Fig S5A–D).

### Moderate CoQ deficiency in *Coq9*^*Q95X*^ mice leads to impaired mitochondrial bioenergetics function

**Figure 6:**
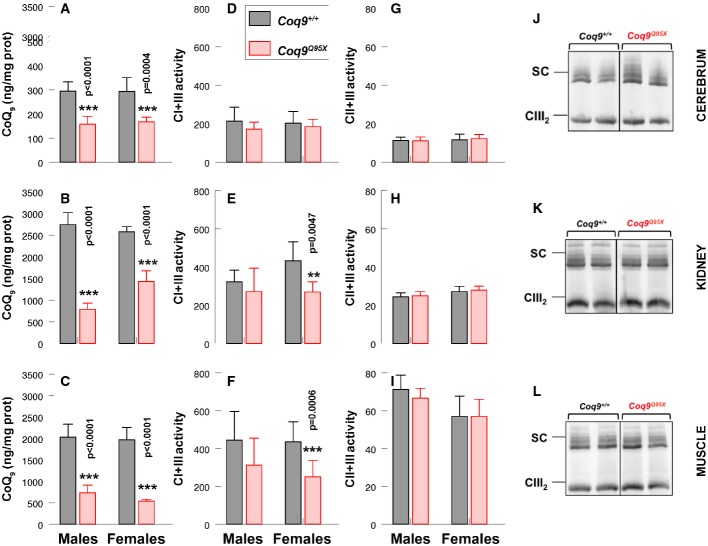
Moderate CoQ deficiency in *Coq9*^*Q95X*^ mice leads to impaired mitochondrial bioenergetics function

To assess whether there was a direct correlation between the tissue CoQ deficiency and the bioenergetics defect, we next evaluated CoQ levels and mitochondrial respiratory chain function in isolated mitochondria from cerebrum, kidney and muscle of *Coq9*^*Q95X*^ and control mice at 6 months of age. Mitochondrial CoQ levels were significantly decreased in cerebrum, kidney and muscle of *Coq9*^*Q95X*^ compared with *Coq9*^+/+^ mice (Fig6A–C), and the level of CoQ deficiency correlated with the CoQ levels measured in tissue homogenates.

A–C Mitochondrial CoQ_9_ levels from cerebrum (A), kidney (B) and skeletal muscle (C) of *Coq9*^+/+^ and *Coq9*^*Q95X*^ males and females. *n* = 8 for each group.

D–F CI+CIII activity in cerebrum (D), kidney (E) and skeletal muscle (F) of male and female *Coq9*^+/+^ and *Coq9*^*Q95X*^ mice. *n* = 6 for each group.

G–I CII+CIII activity in cerebrum (G), kidney (H) and skeletal muscle (I) of male and female *Coq9*^+/+^ and *Coq9*^*Q95X*^ mice. *n* = 6 for each group.

J–L Blue-native gel electrophoresis (BNGE) followed by immunoblotting analysis of mitochondrial supercomplexes from *Coq9*^+/+^ (*n* = 3) and *Coq9*^*Q95X*^ mice (*n* = 4) at 3 months of age. Data information: (A–I) Data are expressed as mean ± SD. Statistical analyses were performed on *Coq9*^+/+^ male mice versus *Coq9*^*Q95X*^ male mice and *Coq9*^+/+^ female mice versus *Coq9*^*Q95X*^ female mice. **P < 0.01; ***P < 0.001. Student's *t*-test. Complex I+III, NADH-cytochrome *c* reductase; complex II+III, SDH-cytochrome *c* reductase. Source data are available online for this figure.

CoQ-dependent mitochondrial CI+III activity was considerably reduced only in kidney and muscle of female *Coq9*^*Q95X*^ mice, while there were no differences in mutant males when compared with the wild-type littermates (Fig6D–F). On the contrary, CoQ-dependent CII+III activities were comparable in mutant and control mice (Fig6G–I). These results correlate with the levels of CoQ because the decrease in CI+III and CII+III activities were more pronounced in *Coq9*^*R239X*^ mice (Garcia-Corzo *et al*, 2013).

The analysis by blue native gel electrophoresis (BNGE) followed by immunoblotting with an anti-core I (complex III subunit) antibody showed that the overall amount of complex III substantially forming SC, as well as the free complex III, was similar in cerebral, kidney and muscle mitochondria of *Coq9*^*Q95X*^ and *Coq9*^+/+^ mice (Fig6J–L). These results differ from those in *Coq9*^*R239X*^ mice, where an increase of free complex III was detected in cerebrum and kidney (Garcia-Corzo *et al*, 2013).

**Figure 7:**
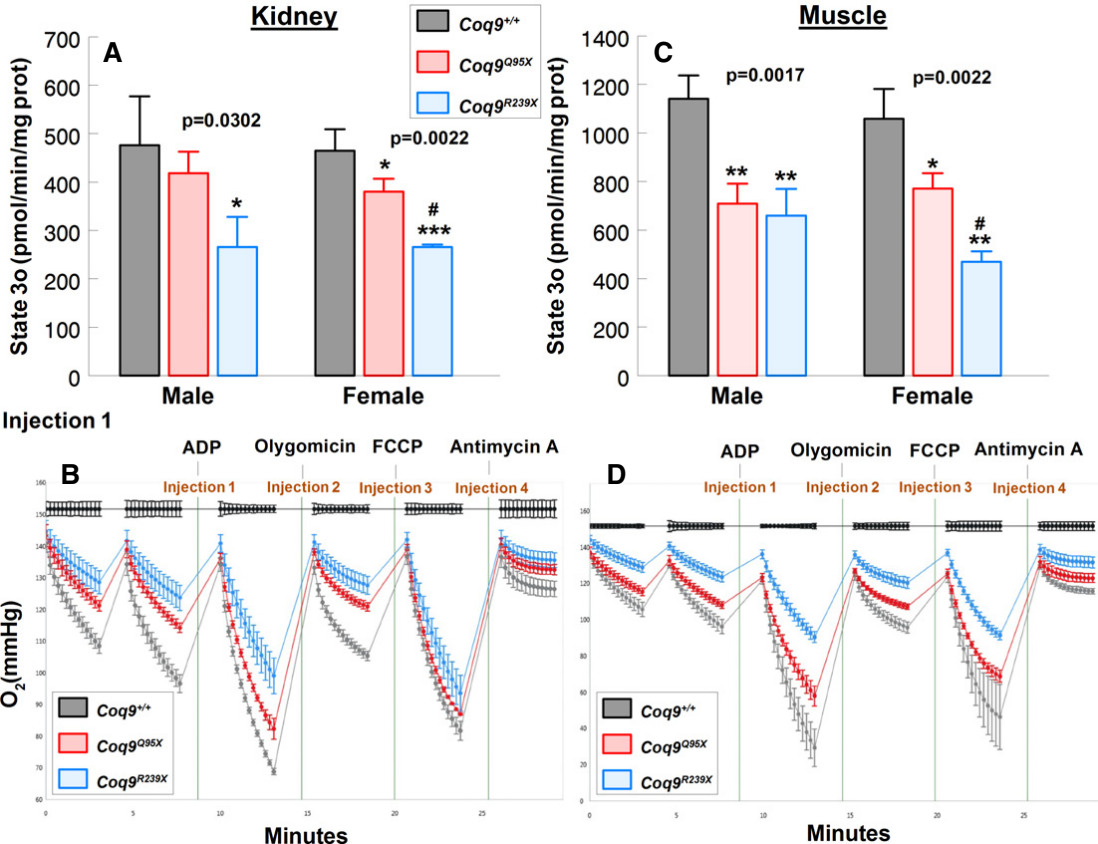
Mitochondrial respiration of *Coq9*^+/+^, *Coq9*^*Q95X*^ and *Coq9*^*R239X*^ mice

The bioenergetics defect in kidney and muscle of *Coq9*^*Q95X*^ mice was confirmed by measurement of mitochondrial O_2_ consumption using isolated mitochondria in the XF^e^24 Extracellular Flux Analyzer (Seahorse Bioscience). In kidney, the phosphorylating respiration (State 3o, in the presence of ADP and substrates) showed a significant decrease in *Coq9*^*Q95X*^ females (82 ± 6%), and *Coq9*^*R239X*^ males and females (56 ± 13 and 57 ± 1%, respectively) compared with wild-type controls (Fig7A and B and Supplementary Fig S7A). In muscle, State 3o was significantly decreased in *Coq9*^*Q95X*^ (62 ± 7% in males and 73 ± 6% in females) and *Coq9*^*R239X*^ mice (58 ± 10% in males and 44 ± 4% in females) (Fig7C and D and Supplementary Fig S7B). In both mutant models, the percentage of decrease in the ADP-stimulated respiration was higher in muscle than in kidney (Fig7A and C). Similar data were obtained in other respiratory states, for example, basal respiration (State 2), resting respiration (State 4, after the addition of oligomycin) and maximal uncoupler-stimulated respiration (State 3u, after the addition of FCCP) (Supplementary Figs S6A–F and S7A and B and Fig7B and D).

A–D Measurement of phosphorylating respiration (represented as State 3o, in the presence of ADP and substrates) in kidney (A) and skeletal muscle (C) from male and female *Coq9*^+/+^, *Coq9*^*Q95X*^ and *Coq9*^*R239X*^ mice at 3 months of age. Representative O_2_ consumption graphic in kidney (B) and skeletal muscle (D) from female *Coq9*^+/+^, *Coq9*^*Q95X*^ and *Coq9*^*R239X*^ mice. Data information: All values are presented as mean ± SD. (A, C) *P < 0.05; **P < 0.01; ***P < 0.001; *Coq9*^*Q95X*^ and *Coq9*^*R239X*^ mice versus *Coq9*^+/+^ mice. ^#^*P* < 0.05; *Coq9*^*Q95X*^ versus *Coq9*^*R239X*^ mice. One-way ANOVA with a Tukey's *post hoc* test. Numbers above columns indicate *P*-values of the one-way ANOVA test (*n* = 3 for each group).

### Morphological evaluation of *Coq9*^*Q95X*^ mice

To assess whether the moderate CoQ deficiency and mitochondrial bioenergetics impairment lead to structural changes in *Coq9*^*Q95X*^ mice tissues, we performed histopathological and histochemical analysis of different sections from cerebrum, kidney and muscle at different ages and compared them with the age- and sex-matched *Coq9*^+/+^ littermates.

Hematoxylin and eosin (H&E) and Luxol fast blue (LFB) stains of cerebrum did not show any structural abnormalities at 3 months of age (Supplementary Fig S8A–D). Likewise, the periodic acid-Schiff (PAS) stain did not reveal histologic alterations in kidney (Supplementary Fig S8E and F). Further evaluation of kidney at 12 and 18 months of age did not show any anatomopathological changes (Supplementary Fig S11A–H). These results, together with the normal biomarkers levels obtained from urine albumin and urea (Supplementary Table S1), suggest that *Coq9*^*Q95X*^ mice did not manifest evidence of kidney diseases associated with CoQ deficiency.

**Figure 8:**
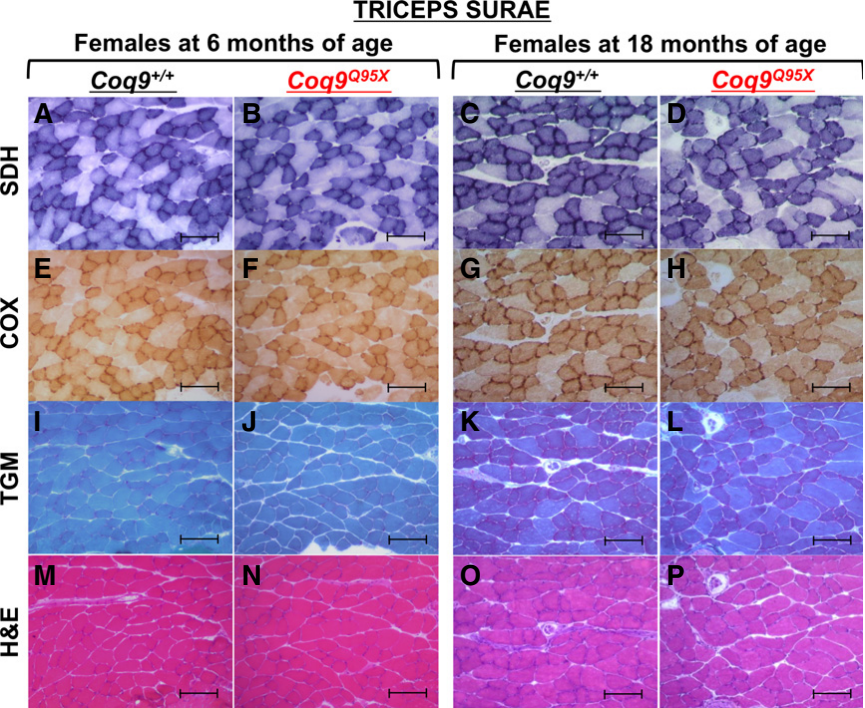
Histopathology of muscle from female *Coq9*^+/+^ and *Coq9*^*Q95X*^ mice at 6 and 18 months of age

In *triceps surae* muscle, we observed round-shaped muscle fibers with central nuclei in one *Coq9*^*Q95X*^ female sample (out of six) (Supplementary Fig S8G–J). To check whether this was an isolated event or it was a sign of muscle pathology, we next performed a histochemical examination of *triceps surae* in controls and homozygous mutant mice at 3, 6, 12 and 18 months of age. In younger *Coq9*^*Q95X*^ mice (3–12 months old), cytochrome *c* oxidase (COX) and succinate dehydrogenase (SDH) activity did not differ compared to *Coq9*^+/+^ littermates (Fig8A, B, E and F and Supplementary Fig S9A–H). Nevertheless, at 18 months, *Coq9*^*Q95X*^ females showed a higher number of COX- and SDH-negative fibers (Fig8C, D, G and H), suggesting that there was a shift from type I fibers (slow-twitch) to type II fibers (fast-twitch). The Gomori trichrome stain did not show signs of mitochondrial proliferation and scattered ragged red fibers (RRF) (Fig8I–L and Supplementary Fig S9I–L). No changes in the overall architecture and general morphology were detected by H&E stain (Fig8M–P and Supplementary Fig S9M–P).

A–H Complex II (SDH) and complex IV (COX) histochemistry of *triceps surae* showing a decreased stain in 18-month-old *Coq9*^*Q95X*^ female mice (D, H) in contrast to normal SDH and COX activity in 6- and 18-month-old *Coq9*^+/+^ (A, C, E, G), as well as 6-month-old *Coq9*^*Q95X*^ female mice (B, F).

I–L Gomori trichrome stain (TGM) of *triceps surae* showed no differences between 6- and 18-month-old *Coq9*^+/+^ and *Coq9*^*Q95X*^ female mice.

M–P Hematoxylin and eosin (H&E) stains of *triceps surae* did not reveal any structural abnormality. Data information: Scale bars: 100 μm. *n* = 3 for each group. Complex IV, cytochrome *c* oxidase (COX); complex II, succinate dehydrogenase (SDH).

Immunohistochemistry with primary anti-glial fibrillary acid protein (GFAP) antibody did not show significant changes in the distribution and number of astrocytes in diencephalon (Supplementary Fig S10A, B, E and F) and pons (Supplementary Fig S10I, J, M and N) of 12-month-old *Coq9*^*Q95X*^. At 18 months of age, there was an overall increase of astrocytes proliferation with no differences between mutants and control animals (Supplementary Fig S10C, D, G, H, K, L, O and P). Heart evaluation at 12 and 18 months of age did not show any anatomopathological changes (Supplementary Fig S11I–P).

### Female *Coq9*^*Q95X*^ mice develop a mild myopathic phenotype with exercise intolerance

**Figure 9:**
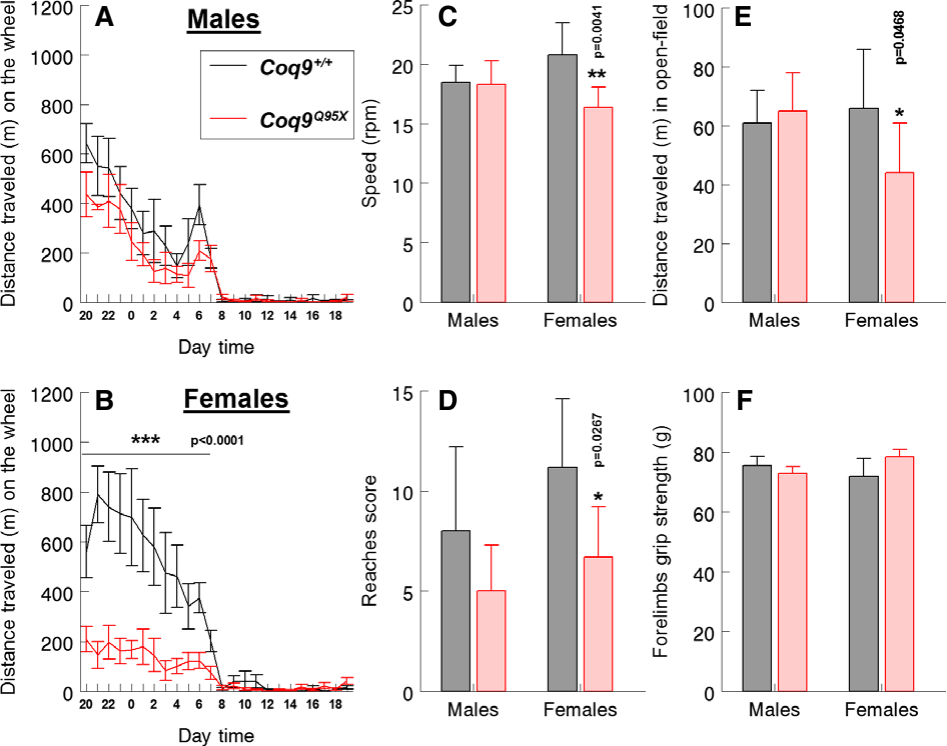
Female *Coq9*^*Q95X*^ mice develop a mild myopathic phenotype with exercise intolerance

Because the muscle was the most impaired tissue in *Coq9*^*Q95X*^ homozygous mice, we assessed the locomotor activity and muscle strength at 6 months of age. Compared to sex-matched wild-type controls, *Coq9*^*Q95X*^ females showed a significant reduction on the average speed during the use of the wheel and spontaneous wheel activity, while there were no differences between mutant and control male animals (Fig9A–C). The decrease in the distance travelled in the home-cage running wheels was corroborated by the observation of reduced spontaneous movement in the open-field test (Fig9E). Likewise, the reaches score obtained in the hanging wire test was lower just in homozygous mutant females (Fig9D). However, muscle strength of forelimbs was not affected (Fig9F). The life span of *Coq9*^*Q95X*^ and *Coq9*^+/+^ mice was similar in both genders.

A–C Voluntary wheel running test. Distance traveled on the wheel and average speed during the use of the wheel were decreased in female *Coq9*^*Q95X*^ mice at 6 months of age (B, C).

D Hanging wire test. *Coq9*^*Q95X*^ female mice obtained less reaches score in the ‘fall and reaches’ method.

E Open-field test. *Coq9*^*Q95X*^ mice showed a reduction in the average distance traveled in *Coq9*^*Q95X*^ female mice at 6 months of age.

F Grip test: Muscle strength was not affected in *Coq9*^*Q95X*^ mice at 6 months of age. Data information: Data are expressed as mean ± SD. Statistical analysis was performed on *Coq9*^+/+^ male mice versus *Coq9*^*Q95X*^ male mice and *Coq9*^+/+^ females versus *Coq9*^*Q95X*^ females. *P < 0.05; **P < 0.01 and ***P < 0.001. Student's *t*-test. *n* = 8 for each group.

### Effects of oral administration of 2,4-dihydroxybenzoic acid (2,4-diHB)

**Figure 10:**
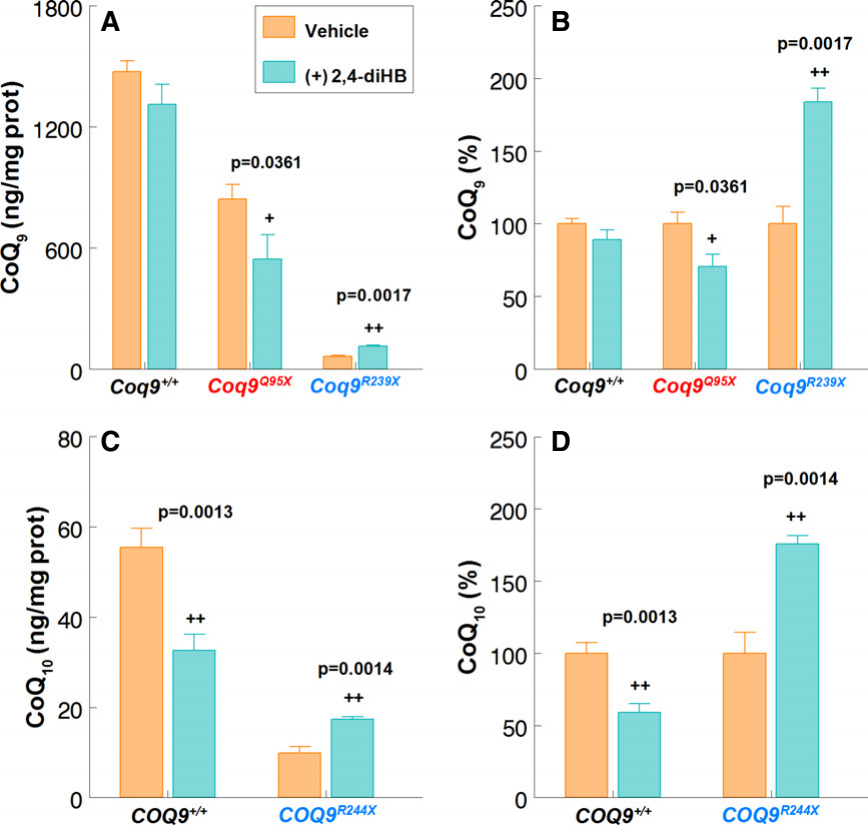
Effects of oral administration of 2,4-dihydroxybenzoic acid (2,4-diHB) in *Coq9*^+/+^, *Coq9*^*Q95X*^ and *Coq9*^*R239X*^ mice and *COQ9*^*R244X*^ patient fibroblasts

As a proof of concept, we also evaluated whether the stability of the CoQ multiprotein complex would affect a possible bypass therapy. For that purpose, we treated *Coq9*^+/+^, *Coq9*^*Q95X*^ and *Coq9*^*R239X*^ mice with oral 2,4-dihydroxybenzoic acid (2,4-diHB), which has been previously tested as a bypass therapy for Δ*coq7 Saccharomyces cerevisiae* strains (Xie *et al*, 2012; Doimo *et al*, 2014). After 1 month of treatment, *Coq9*^*Q95X*^ and *Coq9*^+/+^ mice showed a reduction of kidney CoQ_9_ levels compared with the non-treated littermate (Fig10A and B and Supplementary Fig S12A–D). On the contrary, *Coq9*^*R239X*^ mice treated with 2,4-diHB exhibited significantly higher levels of CoQ_9_ (184 ± 9.3%) compared with untreated *Coq9*^*R239X*^ mice (Fig10A and B and Supplementary Fig S12E and F). Interestingly, this increase in CoQ_9_ levels in *Coq9*^*R239X*^ mice was also observed in the skin fibroblasts from the patient with the homolog *COQ9*^*R244X*^ molecular defect treated with 2,4-diHB (175.8 ± 5.6%), while on control fibroblasts, CoQ_10_ biosynthesis was inhibited by 2,4-diHB supplementation (Fig10C and D and Supplementary Fig S12G and H).

A, B Kidney CoQ_9_ levels in *Coq9*^+/+^, *Coq9*^*Q95X*^ and *Coq9*^*R239X*^ mice treated with 2,4-diHB (+2,4-diHB) compared with the non-treated littermate (vehicle). Statistical analysis was performed on +2,4-diHB *Coq9*^+/+^, *Coq9*^*Q95X*^ and *Coq9*^*R239X*^ mice versus vehicle *Coq9*^+/+^, *Coq9*^*Q95X*^ and *Coq9*^*R239X*^ mice, respectively (*n* = 3 for each group).

C, D CoQ_10_ levels in *COQ9*^*R244X*^ skin fibroblasts treated with 2,4-DiHB (+2,4-diHB) compared with the non-treated controls (vehicle). Statistical analysis was performed on +2,4-diHB *COQ9*^*R244X*^ versus vehicle *COQ9*^*R244X*^ (*n* = 4 for each group). Data information: Data are expressed as mean ± SD. Student' *t*-test. ^+^*P* < 0.05; ^++^*P* < 0.01.

The HPLC chromatographs used to quantify the CoQ levels showed an abnormal peak in *Coq9*^+/+^, *Coq9*^*Q95X*^ and *Coq9*^*R239X*^ mice treated with 2,4-diHB. The retention time of this additional peak was 7.5 min. The mass spectral identification of this lipid exhibited a molecular ion peak of 767.634 [M + H]^+^ and 789.616 [M + Na]^+^ (Fig10I) and could thus be identified as the reduced demethoxyubiquinone 9 (DMQ_9_H_2_) (theoretical mass [C_53_H_82_O_3_] = 767.63422 [M + H]^+^ and 789.61616 [M + Na]^+^).

## Discussion

Primary CoQ_10_ deficiency is an autosomal recessive condition with extremely variable age of onset and clinical manifestations. The reason for the marked diversity in the clinical phenotypes associated with mutations in individual genes remains still unclear (Desbats *et al*, 2014). In this study, we demonstrate that two different premature terminations in the COQ9 protein distinctively affect the levels of other COQ proteins, suggesting that the truncated version of the COQ9 protein produced in the *Coq9*^*R239X*^ mouse model induces a dominant-negative effect on the multiprotein complex for CoQ biosynthesis. As a consequence, the *Coq9*^*R239X*^ mouse model has a global reduction in the COQ proteins, which causes severe CoQ deficiency and clinical phenotype. In contrast, in the new *Coq9*^*Q95X*^ mouse model reported here, the lack of COQ9 protein results in decreased levels of only COQ7 and COQ5 proteins, which leads to moderate CoQ deficiency and a mild mitochondrial myopathy, especially evident in females. Therefore, the stability of this multiprotein complex is a key factor in the CoQ biosynthesis rate and, consequently, in the degree of the severity of CoQ deficiency and in the development of a particular clinical phenotype.

Genetic diseases caused by nonsense or frameshift mutations can generate premature termination codons, which usually trigger nonsense-mediated mRNA decay (NMD). This process is considered to be a surveillance pathway reducing the amount of non-functional mRNA that would produce truncated proteins with dominant-negative or deleterious gain-of-function activities (Brogna & Wen, 2009). Because premature terminations of COQ9 are induced in both mouse models, *Coq9*^*Q95X*^ and *Coq9*^*R239X*^, it was expected a degradation of *Coq9* mRNA by NMD. Accordingly, *Coq9* mRNA was undetectable in cerebrum, kidney and muscle of *Coq9*^*Q95X*^ mice. On the contrary, *Coq9* mRNA was detectable in cerebrum and kidney of *Coq9*^*R239X*^ mice, being the levels around 15% of the control values. As in other genetic diseases (Holbrook *et al*, 2004; Rio Frio *et al*, 2008), the low levels of *Coq9* mRNA are due to NMD because the incubation of *Coq9*^*Q95X*^ and *Coq9*^*R239X*^ MEFs with the NMD inhibitor cyclohexamide increased the *Coq9* mRNA levels. Therefore, the differences in *Coq9* mRNA levels between the two mouse models may account for differences in the efficiency of the NMD to degrade the *Coq9* mRNA containing two nonsense mutations that cause different premature terminations (Inoue *et al*, 2004; Gong *et al*, 2014). A different pattern was, however, observed in muscle, where *Coq9* mRNA levels were almost undetectable in both *Coq9*^*Q95X*^ and *Coq9*^*R239X*^ mice, suggesting that there is tissue specificity in the efficiency of NMD. The existence of this tissue specificity of RNA surveillance has been previously reported in other diseases, for example, osteogenesis imperfecta type I due to premature termination codon mutations *COL1A1* gene (Bateman *et al*, 2003; Zetoune *et al*, 2008). These differences in the efficiency of NMD between tissues are due to variable expression of the NMD factors (Zetoune *et al*, 2008) and contribute to how disease manifests in different tissues (Khajavi *et al*, 2006).

In *Coq9*^*R239X*^ mice, the residual *Coq9* mRNA observed in cerebrum and kidney from incomplete nonsense-mediated decay is translated into an aberrant COQ9 protein without the C-terminal 75 amino acid residues of the mature COQ9 protein. This truncated COQ9 protein may produce a dominant-negative or gain-of-function effect, as it has been reported in other mitochondrial diseases (Tyynismaa *et al*, 2009; Torres-Torronteras *et al*, 2011). The deleterious gain-of-function effect of the truncated COQ9 protein in *Coq9*^*R239X*^ mice affects the stability of the CoQ multiprotein complex since the overall levels of COQ proteins were lower in *Coq9*^*R239X*^ mice than those measured in *Coq9*^*Q95X*^ mice. Accordingly, we propose that the truncation of the COQ9 protein in the *Coq9*^*R239X*^ mouse model would have two consequences: (i) severe and moderate reduction of COQ7 and COQ5 levels, respectively, and (ii) destabilization of the multiprotein complex, decreasing therefore the levels of the other COQ proteins. Similar results in the levels of COQ proteins were obtained by LC-MS/MS in *Coq9*^*R239X*^ mice (Lohman *et al*, 2014), as well as in the skin fibroblasts belonging to the patient with the homologues *COQ9* mutation (*COQ9*^*R244X*^) (Duncan *et al*, 2009). On the contrary, in the *Coq9*^*Q95X*^ mouse model, the absence of the COQ9 protein only affects the levels of COQ7 and COQ5 protein and not the integrity of the multi-subunit complex. While the reason behind the decrease in COQ5 levels is unclear, the decrease in COQ7 levels is justified by the direct physical interaction of COQ9–COQ7, which is needed by COQ9 to expose demethoxyubiquinone, the substrate for the reaction catalyzed by COQ7 (Garcia-Corzo *et al*, 2013; Lohman *et al*, 2014). The different responses of both mutant mice to the treatment with 2,4-diHB also suggest that *Coq9*^*Q95X*^ mice have a stable CoQ mutiprotein complex that is able to regulate CoQ biosynthesis and provide mechanisms of competitive and/or substrate inhibition (Tran & Clarke, 2007; Quinzii *et al*, 2012), in contrast to *Coq9*^*R239X*^ mice. The differences found in the levels of COQ proteins between *Coq9*^*R239X*^ and *Coq9*^*Q95X*^ mice are also supported by the yeasts studies, where phenotypes of certain *COQ* point mutants dramatically differ from the respective null mutants (Belogrudov *et al*, 2001; Baba *et al*, 2004; Tran *et al*, 2006). Moreover, we observed two tissue-specific differences in the COQ protein levels: (i) ADCK3 and COQ6 protein levels were increased only in kidney of *Coq9*^*Q95X*^ mice, and (ii) COQ6 protein level was decreased in skeletal muscle but not in kidney of *Coq9*^*Q95X*^ mice. These divergences could reflect a tissue-specific regulatory feature of CoQ biosynthesis and CoQ multiprotein complex formation.

The imbalance of the CoQ biosynthetic multiprotein complex would explain the severe reduction of CoQ levels in *Coq9*^*R239X*^ mice compared to the moderate CoQ deficiency found in *Coq9*^*Q95X*^ mice. The bioenergetics repercussion of having an intermediate CoQ deficiency was a reduction of CoQ-dependent respiratory complex I+III activity and mitochondrial respiration in kidney and muscle from *Coq9*^*Q95X*^ females. This decrease was not due to the impairment on the distribution between free complex III and supercomplex-associated complex III and may be attributed to the low residual CoQ levels in these tissues (30% of normal). This proportion of the complex I+III activity independent of the supercomplex I-III is supported by our recent study on the effects of ubiquinol-10 supplementation in *Coq9*^*R239X*^ mice, which showed that ubiquinol-10 treatment increases complex I+III activity without increasing the amount of complex III associated to the supercomplex (Garcia-Corzo *et al*, 2014).

Although muscle and kidney of *Coq9*^*Q95X*^ mice had the lowest CoQ content and the most bioenergetics defect, the function and the histologic structure of the kidneys were not affected. This is consistent with the previous study on *Coq9*^*R239X*^ mice, which do not manifest kidney disease either (Garcia-Corzo *et al*, 2013). However, it remains unclear why *Pdss2*^*kd/kd*^ mice develop nephrotic syndrome and Coq9 mutant mice do not (Peng *et al*, 2008; Quinzii *et al*, 2013). On the contrary, histochemical evaluation of muscle revealed an increased number of COX- and SDH-negative fibers in *Coq9*^*Q95X*^ females at 18 months of age, suggestive of a late-onset mild myopathy. This reduction in the muscle mitochondrial energetic activity suggests a skeletal muscle fiber-type transformation from slow fibers (type I) to fast fibers (type II). The changes in fiber-type composition were first reported in an experimental model of respiratory chain myopathy as a compensatory mechanism for the enzymatic deficiency to maintenance muscle strength via increased recruitment of glycolysis for ATP production, at the expense of increased energetic cost (Venhoff *et al*, 2012). Similar to our results, Sommerville *et al* (2013) found an increased frequency of type IIC fibers in morphologically normal muscle biopsies from 18 patients with CoQ_10_ deficiency. Moreover, muscles with a slow/oxidative phenotypic profile contain higher levels of CoQ than muscles with a fast/glycolytic phenotypic profile (Nierobisz *et al*, 2010), suggesting that type I fibers are more susceptible to CoQ deficiency.

Results from the locomotor activity tests also showed a gender difference that is correlated to the bioenergetics and histological findings, that is, *Coq9*^*Q95X*^ females, and not males, had reduced exercise tolerance. Increased susceptibility of female mice to mitochondrial myopathy was also observed in a muscle-specific knockout mouse model of *COX10* (Diaz *et al*, 2005) and may account to the effect of testosterone in muscle mass (Schulte-Hostedde *et al*, 2003). This is consistent with the decreased voluntary activity of androgen receptor knockout male mice (Rana *et al*, 2011). Additionally, it has been reported that the lower levels of CoQ in females could predispose them to a major susceptibility to myopathy associated to statin consumption (Bhardwaj *et al*, 2013). Our results show lower CoQ levels in muscle tissues of females compared to male mice, supporting the concept of a greater sensitivity of female to CoQ deficiency.

In conclusion, our study provides the first evidence of the existence of a multiprotein complex for CoQ biosynthesis in mammals and its importance in determining the degree of CoQ deficiency and the clinical phenotype. Our study suggests that the presence of a COQ9-truncated protein because of an incomplete NMD induces instability of the CoQ mutiprotein complex and contributes in this way to the genetic and tissue-specific pathomechanisms. Furthermore, our work describes the first mouse model of mitochondrial myopathy with exercise intolerance associated to CoQ deficiency, providing new insights to understand the genotype–phenotype disparity associated to CoQ deficiency. Finally, our results may have a potential impact on the treatment of this mitochondrial disorder in two ways: (i) The efficacy of the bypass therapy recently proposed for primary CoQ deficiency caused by molecular defects in proteins of the biosynthetic multicomplex may differ according to the stability of the CoQ multiprotein complex (Xie *et al*, 2012; Doimo *et al*, 2014), and (ii) increasing CoQ levels above 50% of its normal levels may be enough to avoid a severe clinical phenotype.

## Materials and Methods

### Generation of the genetically modified mouse models

The *Coq9*^*Q95X*^ mouse model used in this study was generated by the Wellcome Trust Sanger Institute from ES cell clone EPD0112_2_A09 obtained from the supported KOMP Repository (http://www.komp.org). The ‘knockout first’ cassette was inserted into the C57BL/6N genetic background (project #CSD38115) (Supplementary Fig S13A). Male heterozygous *Coq9*^*Q95X/*+^ mice (C57BL/6N genetic background) were crossbred with female *Coq9*^+/+^ mice under C57BL/6J genetic background. Heterozygous *Coq9*^*Q95X/*+^ mice of the offspring were, consequently, a mix of C57BL/6N and C57BL/6J genetic background (Supplementary Fig S13B). Thus, *Coq9*^*Q95X/*+^ mice were crossbred in order to generate *Coq9*^+/+^*, Coq9*^*Q95X/*+^ *and Coq9*^*Q95X/Q95X*^ (referred in the article as *Coq9*^*Q95X*^).

The *Coq9*^*R239X*^ mouse model was previously generated and characterized under mix of C57BL/6N and C57BL/6J genetic background (Supplementary Fig S13B) (Garcia-Corzo *et al*, 2013).

Only homozygous wild-type and mutant mice from both models were used in the study.

Mice were housed in the Animal Facility of the University of Granada under an SPF zone with lights on at 7:00 AM and off at 7:00 PM. Mice had unlimited access to water and rodent chow. All experiments were performed according to a protocol approved by the Institutional Animal Care and Use Committee of the University of Granada (procedures CEEA 2009-254 and 2010-275) and were in accordance with the European Convention for the Protection of Vertebrate Animals used for Experimental and Other Scientific Purposes (CETS #123) and the Spanish law (R.D. 53/2013). Animals were randomly assigned in experimental groups. Data were randomly collected and processed as well.

### Cells culture and pharmacological treatment

Mouse embryonic fibroblasts (MEFs) from *Coq9*^+/+^, *Coq9*^*Q95X*^ and *Coq9*^*R239X*^ mice, as well as primary mutant and control fibroblasts, were grown in high glucose DMEM-GlutaMAX medium supplemented with 10% FBS, 1% MEM non-essential amino acids and 1% antibiotics/antimycotic. MEFs were treated for 6 h with 28 μg/ml of cycloheximide (Sigma; from a 5 mg/ml stock solution prepared in water) (Rio Frio *et al*, 2008). After treatment, cells were collected and analyzed.

### PCR analyses of regions corresponding to exons 7–11 of *Nnt*

DNA was extracted from the mice tail tips, and PCR of *Nnt* gene was performed as previously described (Mekada *et al*, 2009). *Nnt* gene is complete in the sub-strain C57/BL6N while presents a deletion in exons 7–11 in the sub-strains C57/BL6J. Therefore, exon 6 was used to identify the sub-strains C57/BL6J and C57/BL6N, and exon 7 was used to identify the sub-strain C57/BL6N. To amplify exon 6, we used the following primers: forward, GGGTTTTCGATTGCTGTCATT; reverse, AGTCAGCAGCACTCCTCCAT. To amplify exon 7, we used the following primers: forward, ATTTAGCTGCTGAGGCTGGA; reverse, GACAAAGACCCGAGAAGCAC.

### Proteomic analysis of kidney mitochondria by high-resolution LC-MS/MS

Mitochondrial isolation was performed as describe elsewhere (Fernandez-Vizarra *et al*, 2002). Mitochondrial pellets were solubilized in 200 μl 2% SDS, 100 mM DTT, and 100 mM Tris–HCl, pH 7.4. Proteins were quantified by Bradford, and 50 μg of each extract was digested by filter-aided sample preparation (FASP) method. The tryptic extracts from the wild-type samples (*Coq9*^+/+)^ were analyzed by high-resolution LC-MS/MS in data-dependent mode with an inclusion list. All the COQ9 peptides obtained from the tryptic digestion, in the range from 500 to 4,000, were included in the list (allowing one missed cleavage). One microgram of peptide extract was diluted with 20 μl of 5% MeOH: 1% HCOOH in order to be injected and analyzed by LC-MS/MS. LC-MS/MS spectra were searched using SEQUEST (Proteome Discoverer v1.4; ThermoFisher) and the following parameters: peptide mass tolerance 10 ppm, fragment tolerance 0.02 Da, enzyme set as trypsin and allowance up to three missed cleavages, dynamic modification of methionine oxidation (+ 16 Da) and fixed modification of cysteine carbamidomethylation (+  57 Da). The database used for searching was *Mus musculus*. Peptide identifications were filtered at 1% FDR using the Percolator algorithm included in the Proteome Discoverer software.

The MS system used was an LTQ XL Orbitrap (ThermoFisher) equipped with a nanoESI ion source. A volume of 20 μl from each sample was loaded into the chromatographic system consisting of a C18 preconcentration cartridge (Agilent Technologies) connected to a 15-cm-long, 100 μm i.d. C18 column (Nikkyo Technos Co.). The separation was done at 0.4 μl/min in a 90-min acetonitrile gradient from 3 to 40% (solvent A: 0.1% formic acid, solvent B: acetonitrile 0.1% formic acid). The HPLC system was composed of an Agilent 1200 capillary nano pump, a binary pump, a thermostated micro injector and a micro switch valve. The LTQ XL Orbitrap was operated in the positive ion mode with a spray voltage of 1.8 kV. The spectrometric analysis was performed in a data-dependent mode, acquiring a full scan followed by eight MS/MS scans of the eight most intense signals detected in the MS scan from the global list. The full MS (range 400–1,700) was acquired in the Orbitrap with a resolution of 60,000. The MS/MS spectra were done in the linear–ion trap. From the data-dependent analysis, six COQ9 peptides of the protein were characterized. After the characterization, a targeted method for the analysis of the six detected peptides was designed. Wild-type samples were used to validate the targeted method.

### Quantification of CoQ_9_ and CoQ_10_ levels in mice tissues and mitochondrial fraction

After lipid extraction from homogenized tissues or cultured skin fibroblasts, CoQ_9_ and CoQ_10_ levels were determined via reversed-phase HPLC coupled to electrochemical (EC) detection (Lopez *et al*, 2010; Garcia-Corzo *et al*, 2013). The results were expressed in ng CoQ/mg prot.

### Gene expression analyses

Total cellular RNA from frozen tissue was extracted and electrophoresed in agarose 1.5% to check RNA integrity. RNA from muscle and cerebrum samples was extracted with RNeasy Fibrous Tissue Midi kit (for muscle) and RNeasy Lipid Tissue Mini kit (for cerebrum) (Qiagen, Hilden, Germany) and treated with RNase-Free DNase (Qiagen). RNA from kidney samples was extracted with Real Total RNA Spin Plus Kit (Real). Total RNA was quantified by optical density at 260/280 nm and was used to generate cDNA with High Capacity cDNA Reverse Transcription Kit (Applied Biosystems). Amplification was performed with quantitative real-time PCR, by standard curve method, with specific Taqman probes (from Applied Biosystems) for the targeted gene mouse Coq9 (Mm00804236_m1), Coq7 (Mm00501588_m1), Coq6 (Mm00553570_m1), Coq5 (Mm005018239_m1), Adck3 (mM00469737_m1) and the mouse Hprt probe as a standard loading control (Mm01545399_m1).

### Sample preparation and Western blot analysis in mice tissues

Western blot analyses were performed in cerebrum, kidney and muscle homogenates. Samples were homogenized in buffer A (50 mM Tris–HCl, 1% Triton X-100, 1 mM dithiothreitol, pH 7.6, protease inhibitor cocktail) at 1,100 rpm in a glass–teflon homogenizer. Homogenates were sonicated and centrifuged at 1,000 *g* for 5 min at 4°C, and the resultant supernatant was used for Western blot analysis. 60 μg of proteins from the sample extracts was electrophoresed in 12% Mini-PROTEAN TGX™ precast gels (Bio-Rad) using the electrophoresis system mini-PROTEAN Tetra Cell (Bio-Rad). To detect the truncated version of the COQ9 protein in *Coq9*^*R239X*^ mice, 70 μg of proteins from mitochondrial samples extracts was prepared in XT sample buffer + XT-reducing agent (Bio-Rad) and electrophoresed in a 10% Criterion™ XT precast gel (Bio-Rad) using MOPS running buffer and the electrophoresis system Criterion Cell (Bio-Rad). In all experiments, proteins were transferred onto PVDF 0.45-μm membranes using a mini Trans-blot Cell (Bio-rad) or Trans-blot Cell (Bio-Rad) and probed with target antibodies. Protein–antibody interactions were detected with peroxidase-conjugated horse anti-mouse, anti-rabbit or anti-goat IgG antibodies using Amersham ECL™ Prime Western Blotting Detection Reagent (GE Healthcare, Buckinghamshire, UK). Band quantification was carried out using an Image Station 2000R (Kodak, Spain) and a Kodak 1D 3.6 software. COQ protein band intensity was normalized to Vdac1, and the data expressed in terms of percent relative to wild-type mice (Garcia-Corzo *et al*, 2013).

The following primary antibodies were used: anti-COQ7 (generously provided by Dr Hekimi, McGill University, Canada), anti-COQ6 (Santa Cruz Biotechnology, sc-393932), anti-COQ5 (Proteintech™, 17453-1-AP), anti-ADCK3 (Abnova, M04A) anti-COQ9 (Santa Cruz, sc-271892), anti-COQ9 (Abcam, ab104189) and anti-VDAC1 (Abcam, ab14734).

### Sample preparation and Western blot analysis in human skin fibroblasts

About 1 × 10^5^ cells were collected, washed twice with 1× PBS, homogenated in 1× PBS and sonicated on ice for 10 s. 30 μg of protein was mixed with 4× LDS sample buffer and 25% DDT. After denaturation at 55°C for 5 min, samples were loaded in 12% SDS–PAGE gel. Proteins were transferred on PDVF membranes and incubated overnight at 4°C with primary antibodies: 1:100 COQ7 rabbit polyclonal antibody (Thermo Scientific; PA5-25774), 1:100 COQ6 rabbit polyclonal antibody (Abcam; ab128652), 1:100 COQ9 rabbit polyclonal antibody (Thermo Scientific; PA5-24816), 1:100 COQ5 rabbit polyclonal antibody (Thermo Scientific; PA5-26327), 1:100 COQ8/ADCK3 mouse monoclonal antibody (Abnova; H00056997-M04A) and 1:1,000 vinculin monoclonal mouse antibody (Abcam SPM227), used as a loading control. Protein–antibody interaction was detected by peroxidase-conjugated mouse antibody, peroxidase-conjugated rabbit antibody or peroxidase-conjugated goat antibody using ECL Prime Western Blotting Detection Reagents (GE Healthcare). Band intensity was assessed by Image J.

### CoQ-dependent respiratory chain activities

CoQ-dependent respiratory chain activities (CI + III and CII + III) were measured in submitochondrial particles as described elsewhere (Kirby *et al*, 2007; Garcia-Corzo *et al*, 2013). The results were expressed in nmol reduced cyt c/min/mg prot.

### Evaluation of supercomplex formation by BNGE

BNGE was performed on the mitochondrial fraction from mice tissues. Mitochondrial isolation was performed as previously described (Fernandez-Vizarra *et al*, 2002; Garcia-Corzo *et al*, 2013). Mitochondrial membrane proteins (100 μg) were applied and run on a 3–13% first-dimension gradient BNGE gel as previously described (Schagger, 2001; Acin-Perez *et al*, 2008; Garcia-Corzo *et al*, 2013). After electrophoresis, the complexes were electroblotted onto PVDF filters and sequentially probed with specific antibodies against complex III, anti-ubiquinol-Cytochrome *c* Reductase Core Protein I (Abcam, ab110252).

### Mitochondrial respiration

To isolate fresh mitochondria, mice were sacrificed and the organs were extracted rapidly on ice. Muscle (*triceps surae* and *vastus lateralis*) was submerged in 1 mg/ml proteinase K solution for 60 s. Then, muscle was homogenized (1: 10, w/v) in isolation buffer (250 mM sucrose, 2 mM EDTA, 10 mM Tris, 0,5% free fatty acids albumin, pH 7.4) at 800 rpm at 4°C with a glass–teflon homogenizer. The homogenate was centrifuged twice at 1,000 *g* for 5 min at 4°C, and the supernatant was centrifuged at 23,000 *g* for 10 min at 4°C. Then, the mitochondrial pellet was resuspended in 100 μl of isolation buffer, and a 10 μl aliquot was used for protein determination. The remaining sample was washed with 900 μl of isolation buffer and centrifuged at 13,000 *g* for 3 min at 4°C. The final crude mitochondrial pellet was resuspended in 90 μl MAS 1× medium [70 mM sucrose, 220 mM mannitol, 10 mM KH_2_PO_4_, 5 mM MgCl_2_, 2 mM HEPES, 1 mM EGTA and 0.2% (w/v) fatty acid-free BSA, pH 7.2]. Kidney was homogenated (1:10, w/v) in a respiration buffer A (250 mM sucrose, 0.5 mM Na_2_EDTA, 10 mM Tris and 1% free fatty acid albumin) at 800 rpm in a glass–teflon homogenizer. Then, homogenate was centrifuged at 500 *g* for 7 min at 4°C, and the supernatant was centrifuged at 7,800 *g* for 10 min at 4°C. The pellet was then resuspended in respiration buffer B (250 mM sucrose, 0.5 mM Na_2_EDTA and 10 mM Tris), and a 5 μl aliquot was used for protein determination. The remaining sample was then centrifuged at 6,000 *g* for 10 min at 4°C. The pellet was resuspended in buffer A and centrifuged again at 6,000 *g* for 10 min at 4°C. The final crude mitochondrial pellet was re-suspended in 95 μl MAS 1× medium.

Mitochondrial respiration was measured by using an XF^e^24 Extracellular Flux Analyzer (Seahorse Bioscience) (Rogers *et al*, 2011). Mitochondria were first diluted to the needed concentration required for plating in cold 1× MAS (2.5 μg/well in kidney; 1.5 μg/well in muscle). Next, 50 μl of mitochondrial suspension was delivered to each well (except for background correction wells) while the plate was on ice. The plate was then centrifuged at 2,000 *g* for 10 min at 4°C. After centrifugation, 450 μl of 1× MAS + substrate (10 mM succinate, 2 mM malate, 2 mM glutamate and 10 mM pyruvate) was added to each well. Respiration by the mitochondria was sequentially measured in a coupled state with substrate present (basal respiration or State 2), followed by State 3o (phosphorylating respiration, in the presence of ADP and substrates); State 4 (non-phosphorylating or resting respiration) was measured after the addition of oligomycin when all ADP was consumed, and then maximal uncoupler-stimulated respiration (State 3u). Injections were as follows: port A, 50 μl of 40 mM ADP (4 mM final); port B, 55 μl of 30 μg/ml oligomycin (3 μg/ml final); port C, 60 μl of 40 μM FCCP (4 μM final); and port D, 65 μl of 40 μM antimycin A (4 μM final). All data were expressed in pmol/min/μg protein.

### Histology and immunohistochemistry

Cerebrum, heart and kidney were formalin-fixed and paraffin-embedded. Multiple sections (4 μm) were deparaffinized with xylene and stained with hematoxylin and eosin (H&E), Masson's trichrome (TCM), periodic acid–Schiff (PAS) and Luxol fast blue (LFB) (Garcia-Corzo *et al*, 2013). Immunohistochemistry was carried out in the same sections, using the following primary antibodies: glial fibrillary acidic protein or anti-GFAP (Millipore, MAB360), anti-oligodendrocytes (Millipore, MAB1580) and neuronal class III β-tubulin anti-TUJ1 (Covance, MMS-435P) (Garcia-Corzo *et al*, 2013). Dako Animal Research Kit for mouse primary antibodies (Dako Diagnóstico S.A., Spain) was used for the qualitative identification of antigens by light microscopy. Sections were examined at 40–400× magnifications with an OLYMPUS CX41 microscope, and the images were scanned under equal light conditions with the CELL A computer program.

Muscle samples (*triceps surae*) were snap-frozen in isopentane cooled in liquid nitrogen. Cross sections (8 μm thick) of frozen muscle were stained for succinate dehydrogenase (SDH) and cytochrome oxidase (COX) activities (Tanji & Bonilla, 2008). Muscle sections were also stained with hematoxylin–eosin and Gomori Trichrome to assess muscle fiber area and general morphology (Tanji & Bonilla, 2008).

### Determination of the metabolite profile in urine

Urine samples were collected for 24 h and analyzed in a BS-200 Clinical Chemistry Analyzer (Mindray Medical España S.L., Spain) at 37°C. The following colorimetric tests were performed: urea and albumin (Linear Chemicals S.L., Spain) (Garcia-Corzo *et al*, 2013).

### Assessment of the locomotor activity

Locomotor activity was tested in age-matched mice (6 months old) with a similar averaged body weight. Voluntary wheel running was assessed in polycarbonate cages (20.5 cm wide × 36.5 cm long × 14 cm high) with free access to stainless steel activity wheels (diameter 23 cm; width 5 cm) with a ball-bearing axle (Bioseb, Boulogne, France). The wheels were connected to a computer that automatically recorded the distance traveled by the mice per hour of recording, as well as the duration and speed of every running bout during the whole recording period. Spontaneous wheel running was monitored continuously during 48 h, starting at the beginning of the dark period (20:00 h), and the results obtained in the 2 days of evaluation were averaged. Animals had food and water available *ad libitum* and were trained to the wheels for 1 day before the data collection period (Cobos *et al*, 2012).

The open-field test consisted of a square arena in a ground space of 25 × 25 × 25 cm. Walls were opaque, so the animals could not see the room. Each mouse was placed in the center of the square arena between 8 and 9 PM under red light exposure and its movement monitored through the video-tracking system SMART® (Panlab S.L., Spain) for 30 min after an adaption period of 30 min (Pallud *et al*, 2011). Distance travelled (cm) of each mouse was quantified (Garcia-Corzo *et al*, 2013).

Muscle strength was assessed using a computerized grip strength meter (Model 47200, Ugo-Basile, Varese, Italy). The experimenter held the mouse gently by the base of the tail, allowing the animal to grab the metal bar with the forelimbs before being gently pulled until it released its grip. The peak force of each measurement was automatically recorded by the device and expressed in grams (g). The forelimb grip strength of each mouse was measured in duplicate with at least 1 min between measurements.

For the hanging wire test, we chose the ‘falls and reaches’ method (Raymackers *et al*, 2003). Mice were subjected to a 180 s lasting hanging test, during which a ‘reaching’ score is recorded. At the beginning of the test, each animal was given a reaching score of 0. Animals were suspended by their forelimbs to a 1.5-mm-thick, 55-cm-long metallic wire suspended 45 cm above soft ground, and then, the timer was started.

The timer was stopped anytime the animal fell and restarted when it was placed again on the wire until 180 s. If the animal reached one end of the wire, timer was stopped and ‘reaching’ score was increased by 1. Results were expressed as the ‘average of reaches score’.

### Supplementation with 2,4-diHB

The supplementation procedure in mice consisted of administering 2,4-diHB in the drinking water in a dose of 1 g/kg bw/day. The treatment started at 1 month of age, and the mice were sacrificed at 2 months of age. The drinking water was changed twice a week. The supplementation procedure in primary fibroblasts consisted of administering 0.5 mM or 2.5 mM 2,4-diHB. A control group with vehicle (DMSO) at the same dose was also studied. Cells were collected 1 week after the supplementation.

### Ultra carrying out liquid chromatography–mass spectrometer (MS/MS) analysis of intermediate metabolites

Lipid extracts were obtained as described above for the CoQ quantification. Samples were analyzed using an Acquity Ultra-Performance liquid chromatography system coupled to a high definition QTOF SynaptG2 detector of mass spectrometry (MS/MS) (Waters Corporation). The analytical separation column was a BEH C18, 1.7 μm, 2.1 × 50 mm column (Waters, Spain). The mobile phase consisted of methanol and 0.1% formic acid at the constant flow rate of 0.5 ml/min. Source and probe temperatures were set at 100 and 500°C, respectively. Nitrogen was used as both cone gas (30 l/h) and desolvation gas (600 l/h). Acquisition range was between 50 and 1,200 uma.

### Statistical analysis

All statistical analyses were performed using the GraphPad scientific software. Data are expressed as the mean ± SD of 3–10 experiments per group. A one-way ANOVA with a Tukey's *post hoc* test was used to compare the differences between three experimental groups. Studies with two experimental groups were evaluated using unpaired Student's *t*-test. A *P*-value of 0.05 was considered to be statistically significant.

Effect size was calculated using the application available in http://www.biomath.info/power/ttest.htm. Number of animals in each group were calculated in order to detect gross ~60% changes in the biomarkers measurements (based upon α = 0.05 and power of β = 0.8) using the application available in http://www.biomath.info/power/index.htm. The Gaussian distribution was checked using GraphPad Software: http://graphpad.com/quickcalcs/probability1.cfm.

**The paper explained**

**Problem**

The biosynthesis of coenzyme Q10 (CoQ_10_) occurs in mitochondria and involves at least 11 different proteins that are associated, at least in yeasts, in a multiprotein complex. Primary CoQ_10_ deficiency is due to mutations in genes involved in CoQ biosynthesis. The disease has been associated with six major phenotypes: (i) encephalomyopathy, (ii) severe infantile multisystemic disease, (iii) nephropathy, (iv) cerebellar ataxia, (v) isolated myopathy, and (vi) multiple system atrophy. Curiously, mutations in the same gene may cause different phenotypes; for example, mutations in *COQ2* and *COQ6* have been indistinctly attributed to nephropathy or multisystemic disease. To try to understand genotype–phenotype disparities, we compare two mouse models with a genetic modification in *Coq9* gene, that is, *Coq9*^*Q95X*^ and *Coq9*^*R239X*^.

**Results**

Contrary to *Coq9*^*R239X*^, which manifests severe widespread CoQ_10_ deficiency associated with fatal encephalomyopathy, *Coq9*^*Q95X*^ mice exhibited mild CoQ deficiency manifesting with reduction in CI+III activity and mitochondrial respiration in skeletal muscle, leading to a late-onset mild mitochondrial myopathy with decreased locomotor activity. Moreover, 2,4-dihydroxybenzoic acid (2,4-diHB) supplementation increased the levels of CoQ_9_ only in *Coq9*^*R239X*^ mice. We show that these differences were due to the levels of COQ biosynthetic proteins, suggesting that the presence of a truncated version of COQ9 protein in *Coq9*^*R239X*^ mice destabilizes the CoQ multiprotein complex.

**Impact**

Our study provides the first evidence of the existence of a multiprotein complex for CoQ biosynthesis in mammals and its importance in determining the degree of CoQ deficiency and the clinical phenotype. Our study suggests that the presence of a COQ9-truncated protein because of an incomplete nonsense-mediated mRNA decay (NMD) induces instability of the CoQ mutiprotein complex and contributes in this way to the genetic and tissue-specific pathomechanisms. Furthermore, our work describes the first mouse model of mitochondrial myopathy with exercise intolerance associated to CoQ deficiency, providing new insights to understand the genotype–phenotype disparity associated to CoQ deficiency and may have a potential impact on the treatment of this mitochondrial disorder.

## For more information

Online Mendelian Inheritance in Man (OMIM)

COQ9: S. Cerevisiae, homolog of; COQ9

http://omim.org/entry/612837#0001

International mito-patients

http://www.mitopatients.org/index.html

The association of mitochondrial disease patients in Spain

http://www.aepmi.org/publicoIngles/index.php

The United Mitochondrial Disease Foundation

http://www.umdf.org/

## Supporting Information

Source Data for Supplementary Figure S4

Review Process File

Source Data for Figure 1B

Source Data for Figure 2E
