# Supplementary figures and images for: The clinical heterogeneity of coenzyme Q10 deficiency results from genotypic differences in the Coq9 gene
Source: EMBO Mol Med. 2015 Mar 23;7(5):670–87. doi: 10.15252/emmm.201404632 (PMC4492823; doi:10.15252/emmm.201404632)

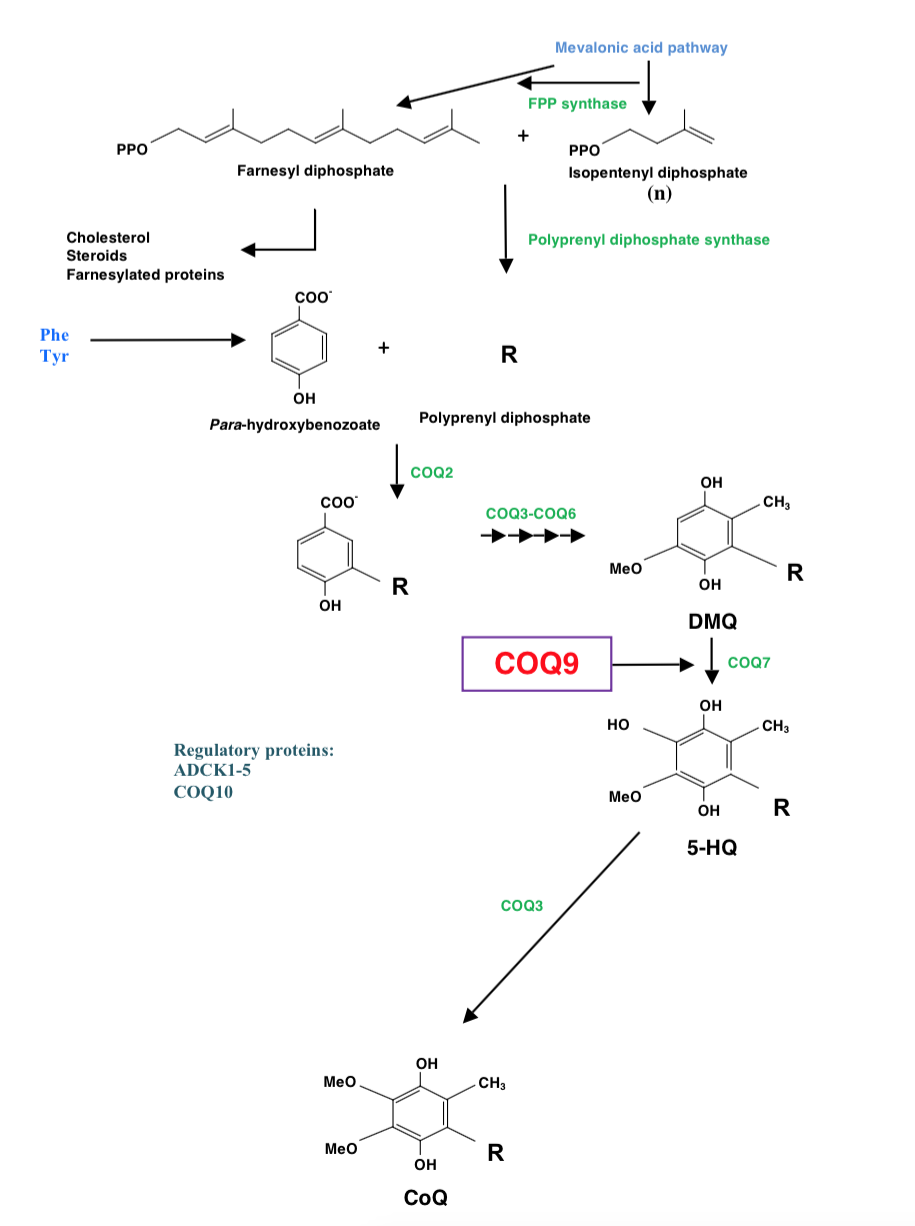

Supplement: Supplementary file 1 [file emmm0007-0670-sd1.tif]

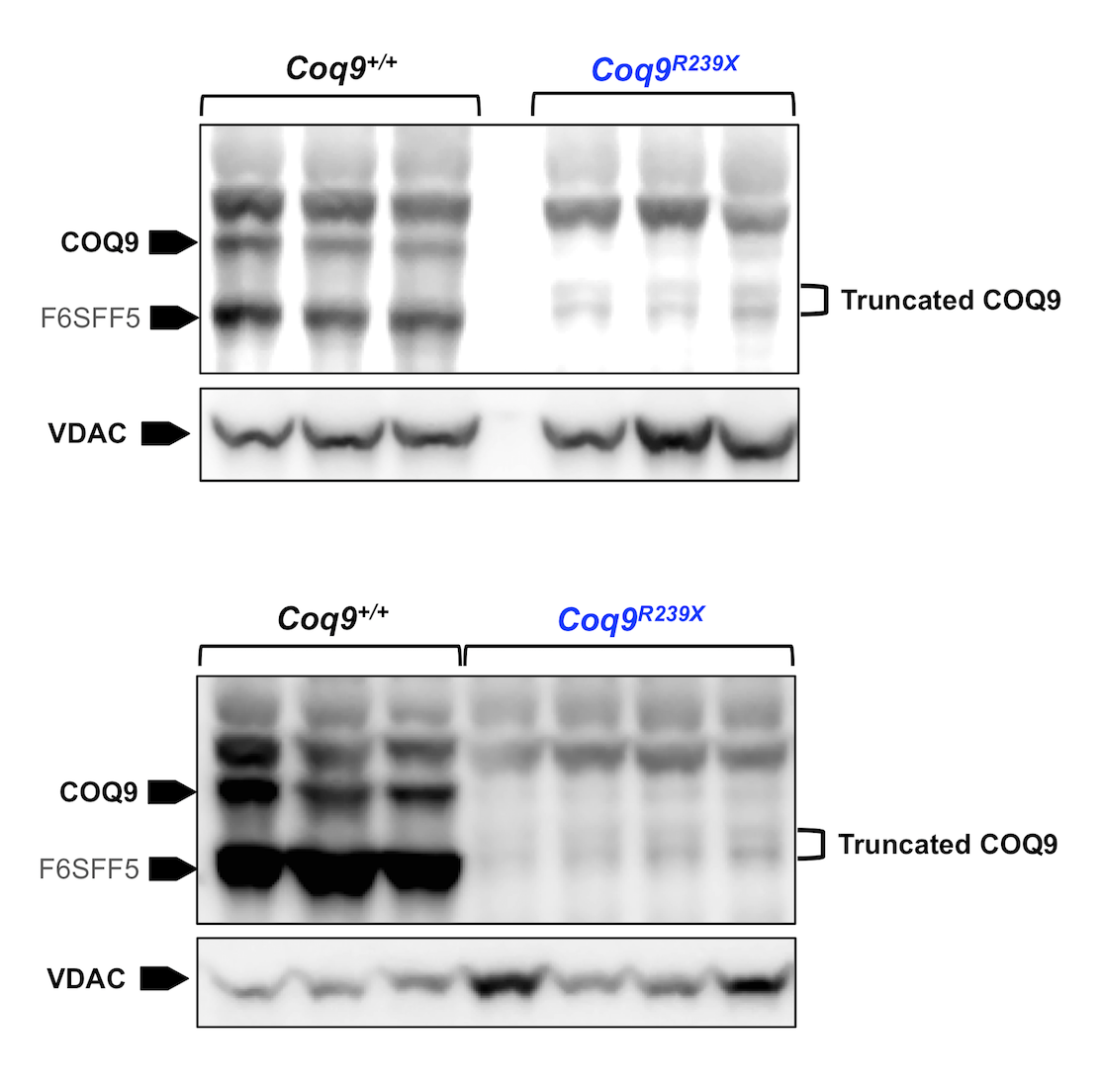

Supplement: Supplementary file 2 [file emmm0007-0670-sd2.tif]

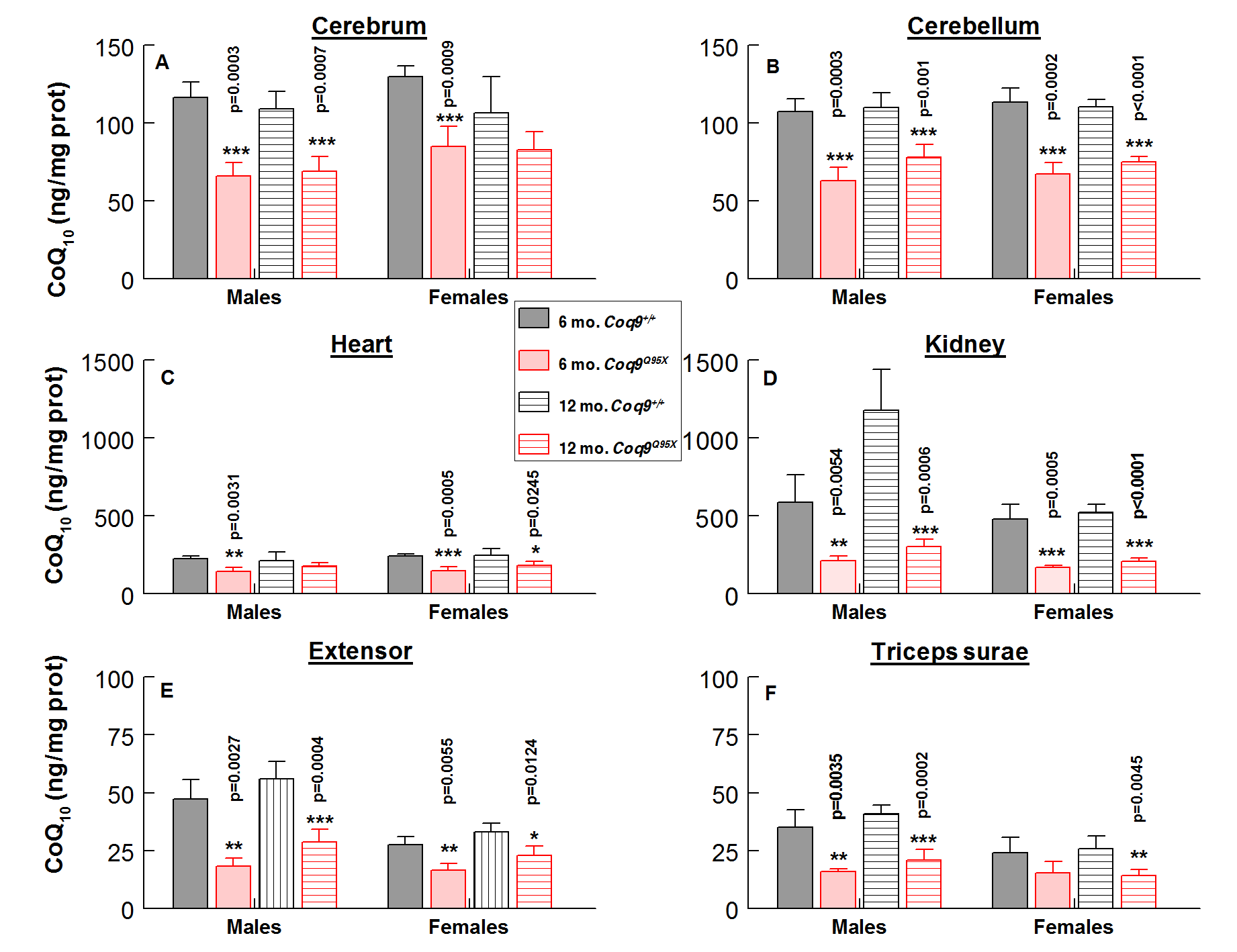

Supplement: Supplementary file 3 [file emmm0007-0670-sd3.tif]

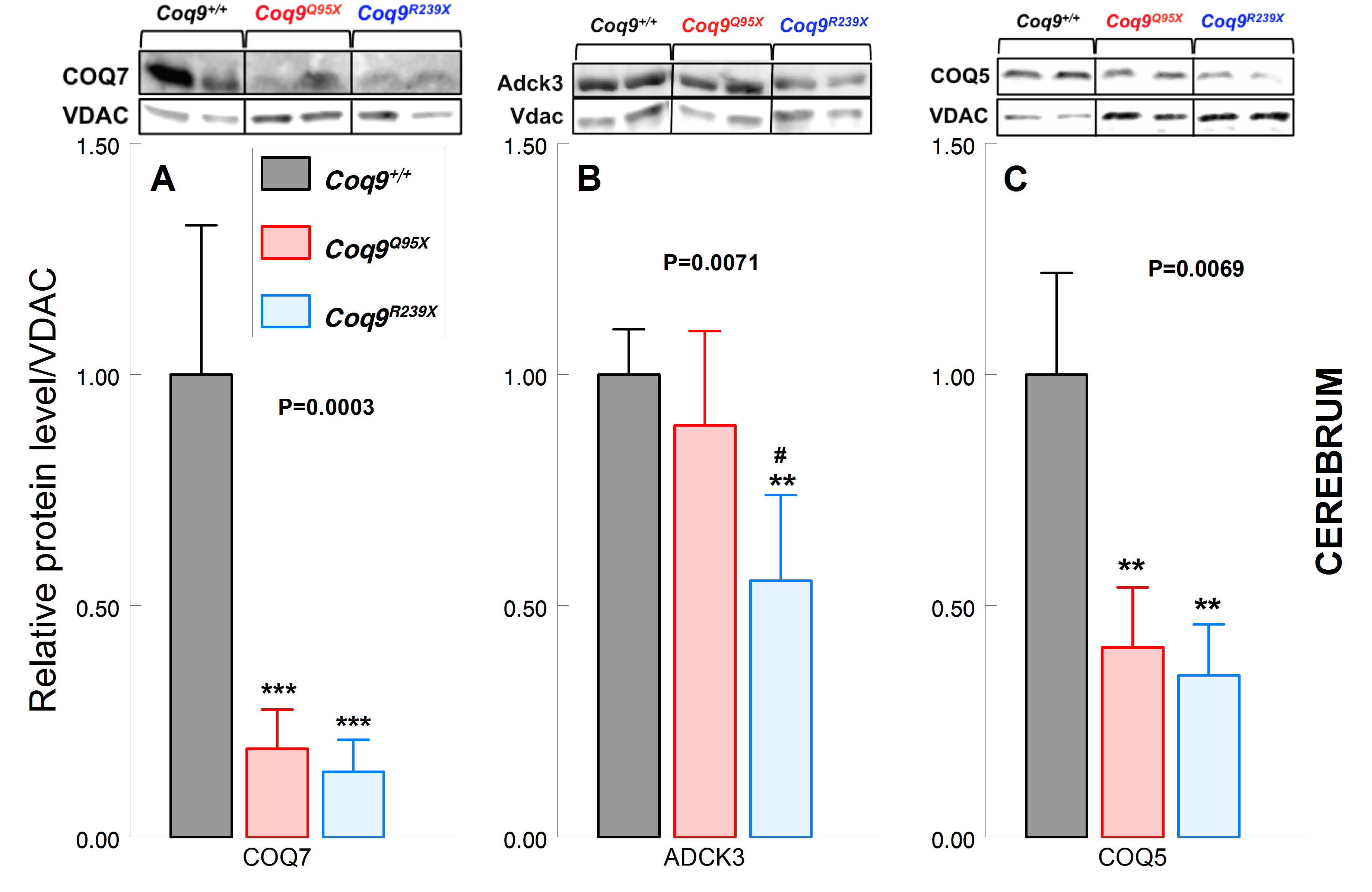

Supplement: Supplementary file 4 [file emmm0007-0670-sd4.tif]

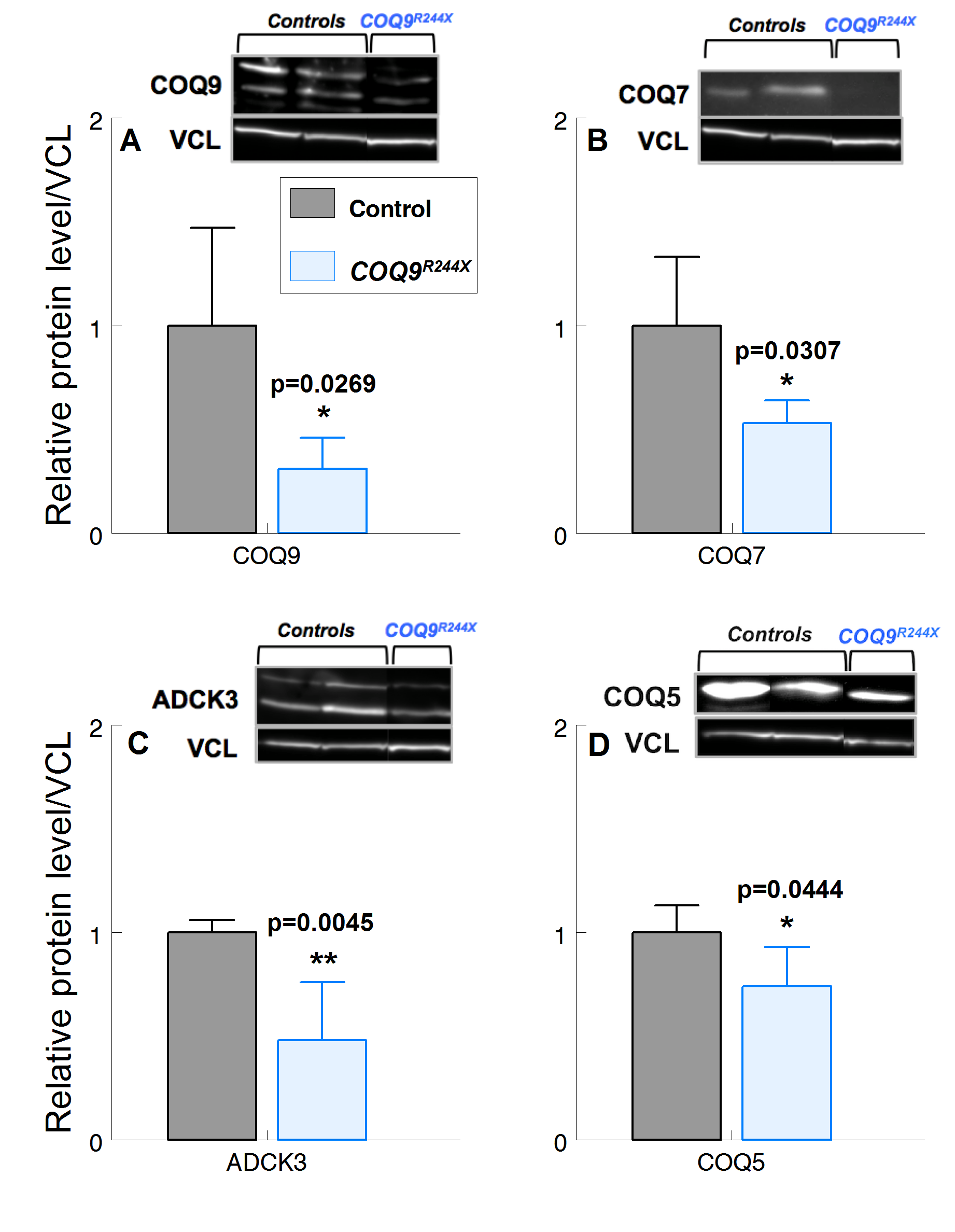

Supplement: Supplementary file 5 [file emmm0007-0670-sd5.tif]

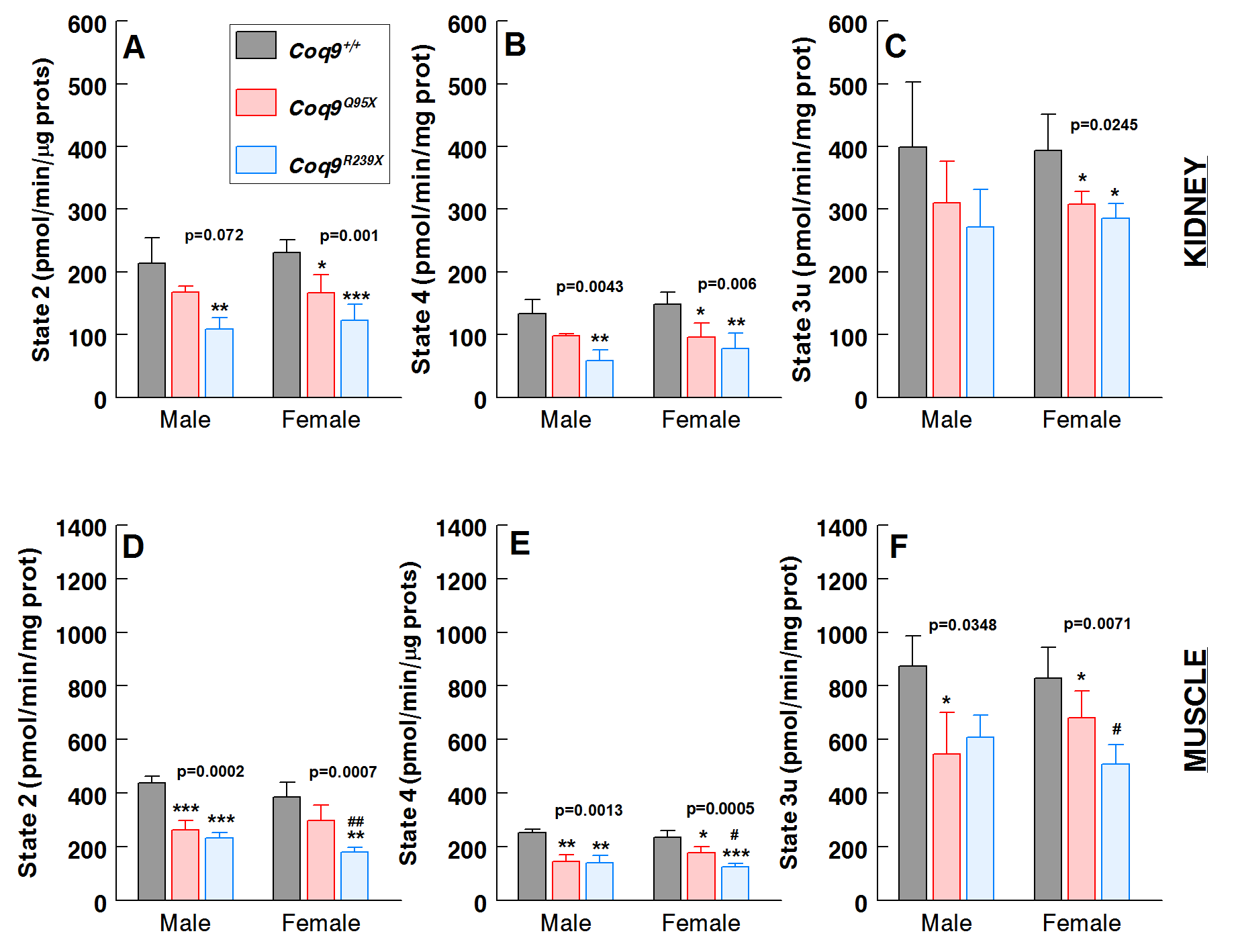

Supplement: Supplementary file 6 [file emmm0007-0670-sd6.tif]

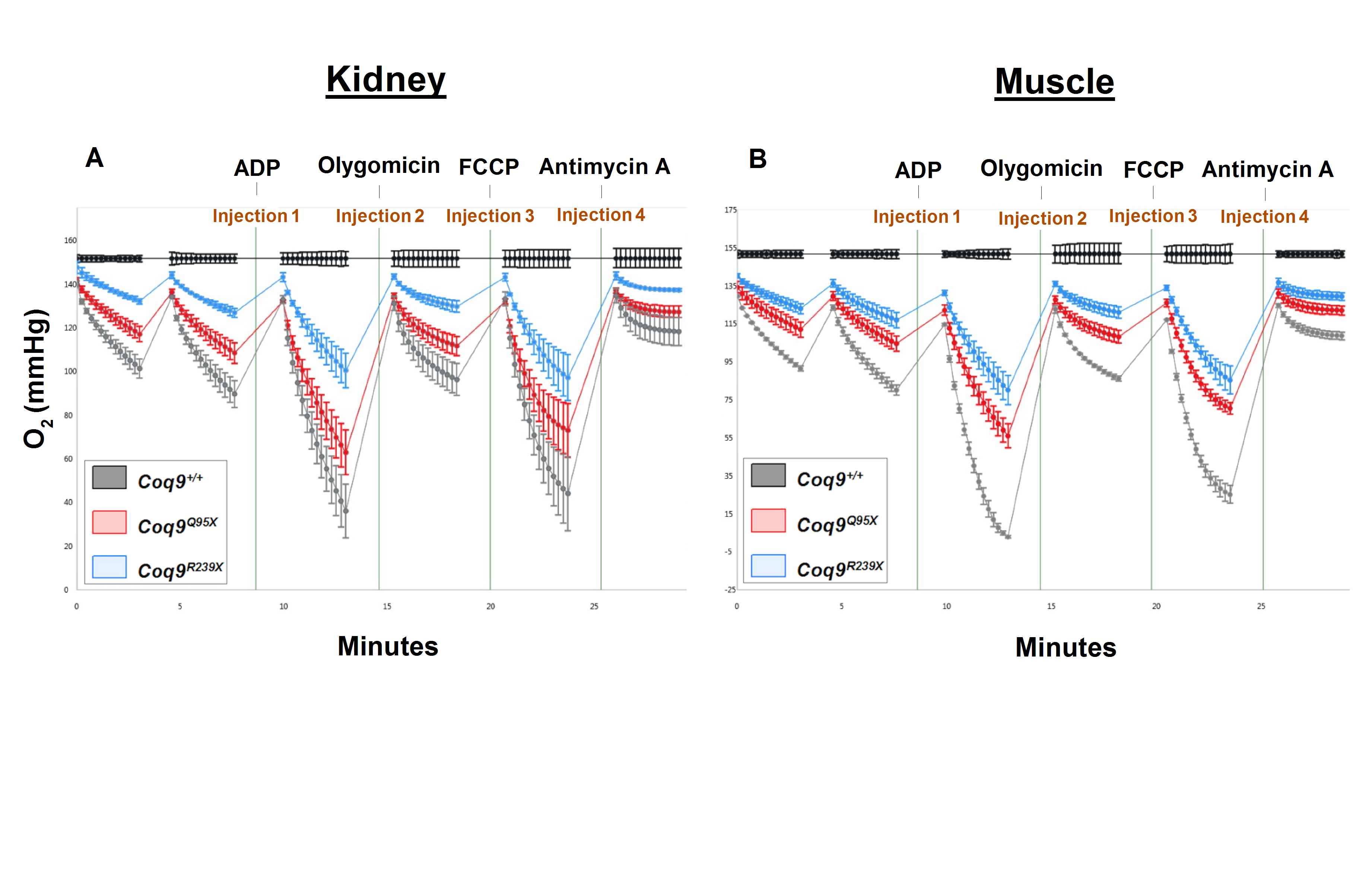

Supplement: Supplementary file 7 [file emmm0007-0670-sd7.tif]

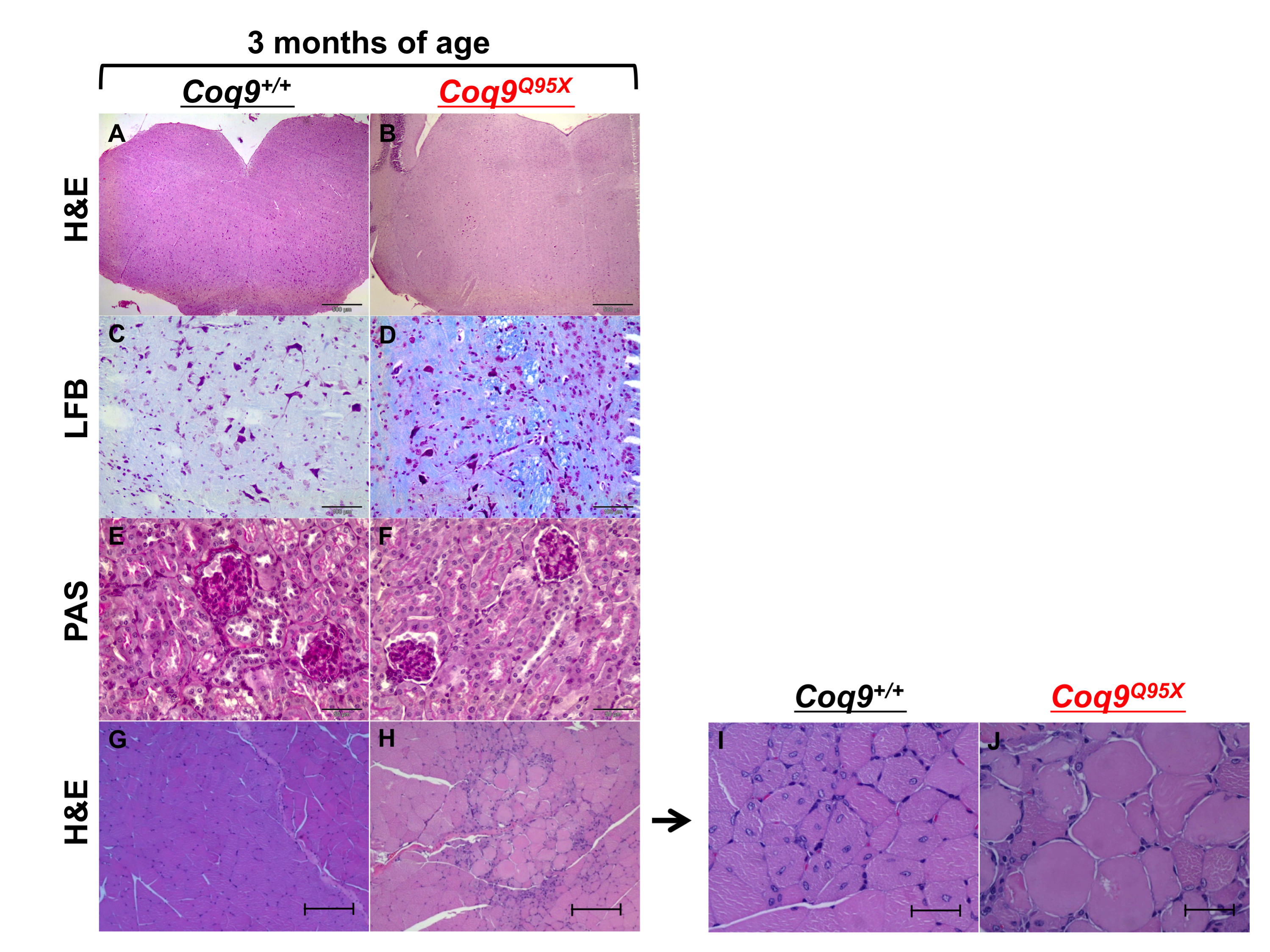

Supplement: Supplementary file 8 [file emmm0007-0670-sd8.tif]

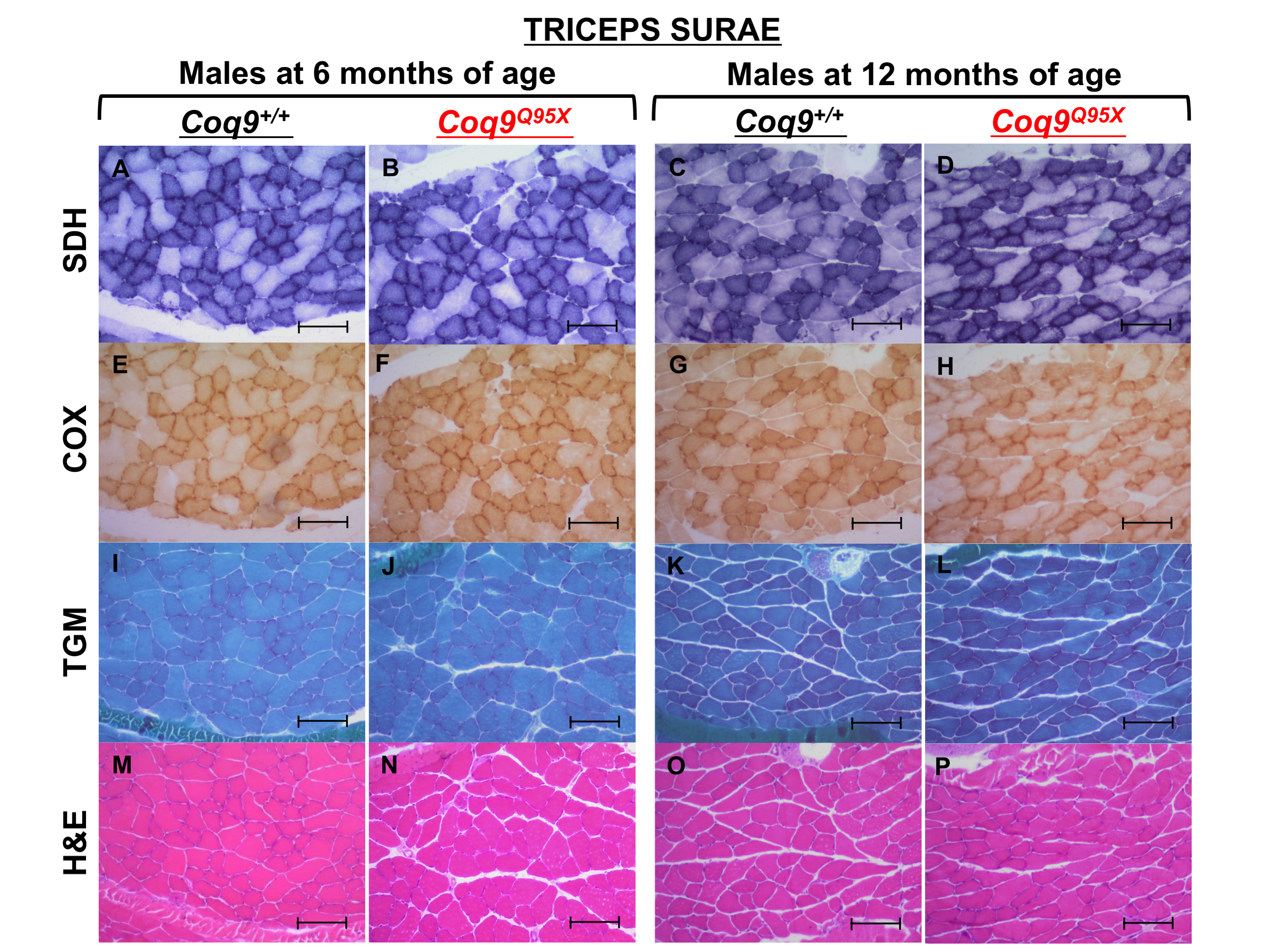

Supplement: Supplementary file 9 [file emmm0007-0670-sd9.tif]

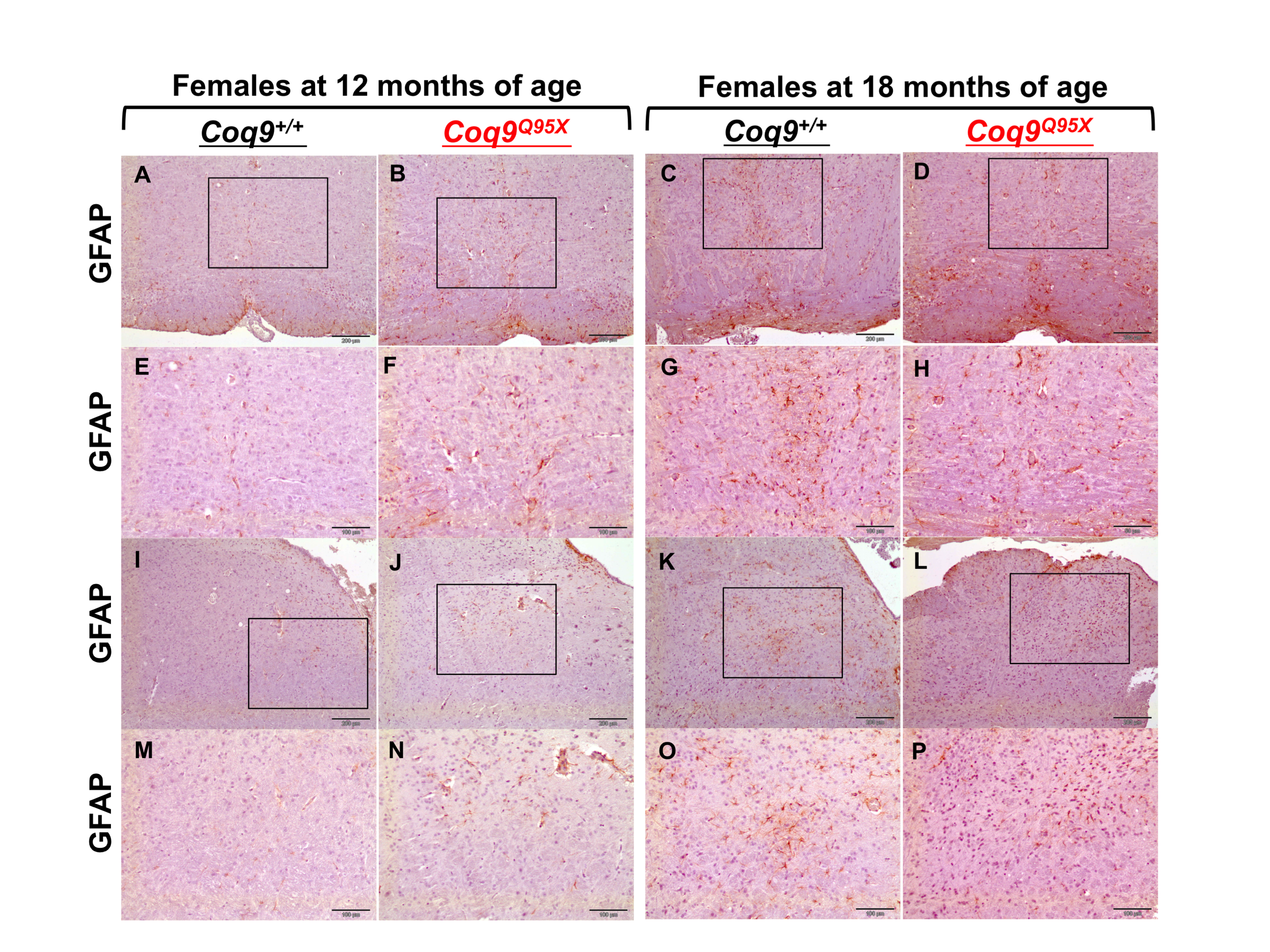

Supplement: Supplementary file 10 [file emmm0007-0670-sd10.tif]

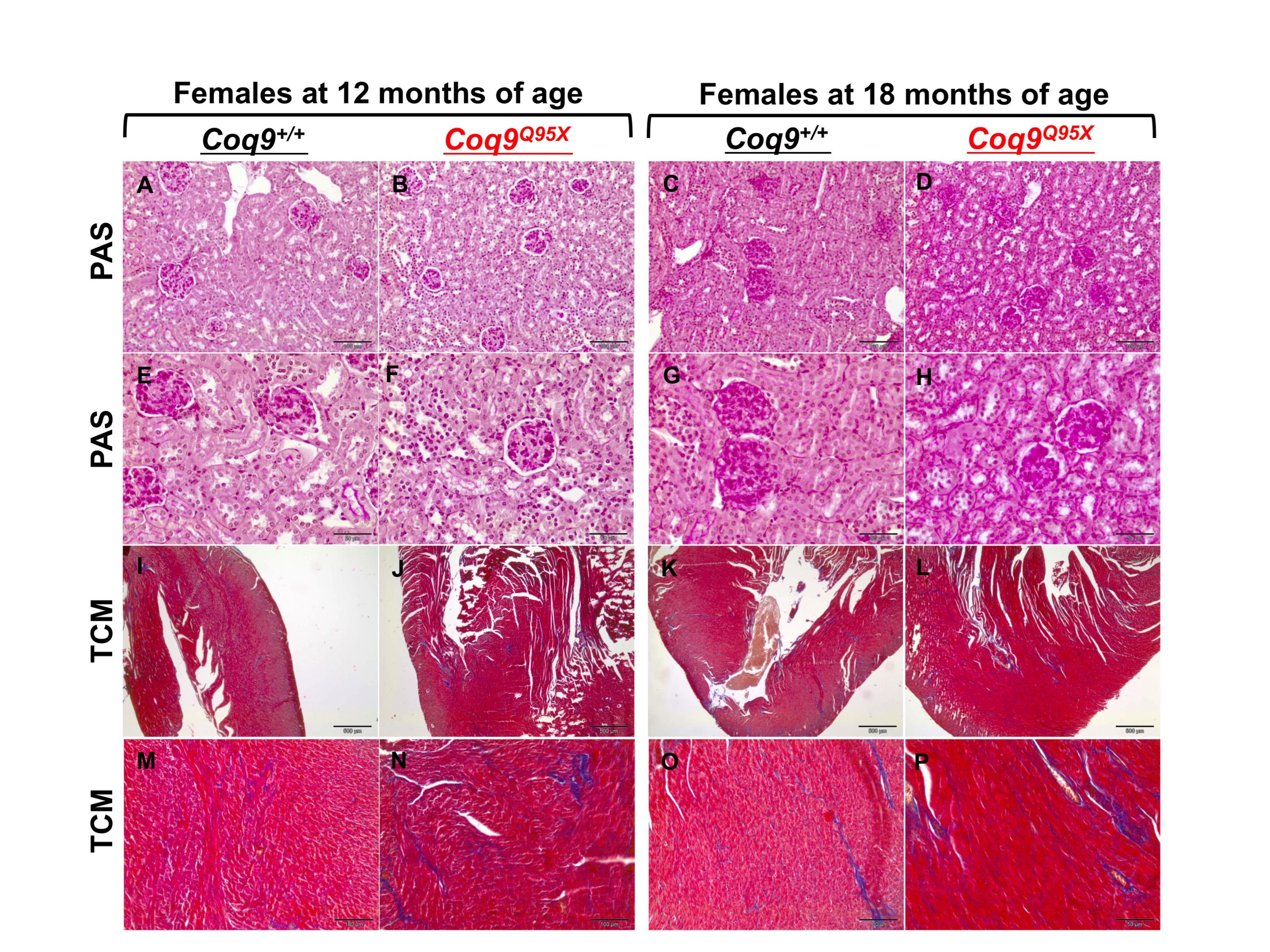

Supplement: Supplementary file 11 [file emmm0007-0670-sd11.tif]

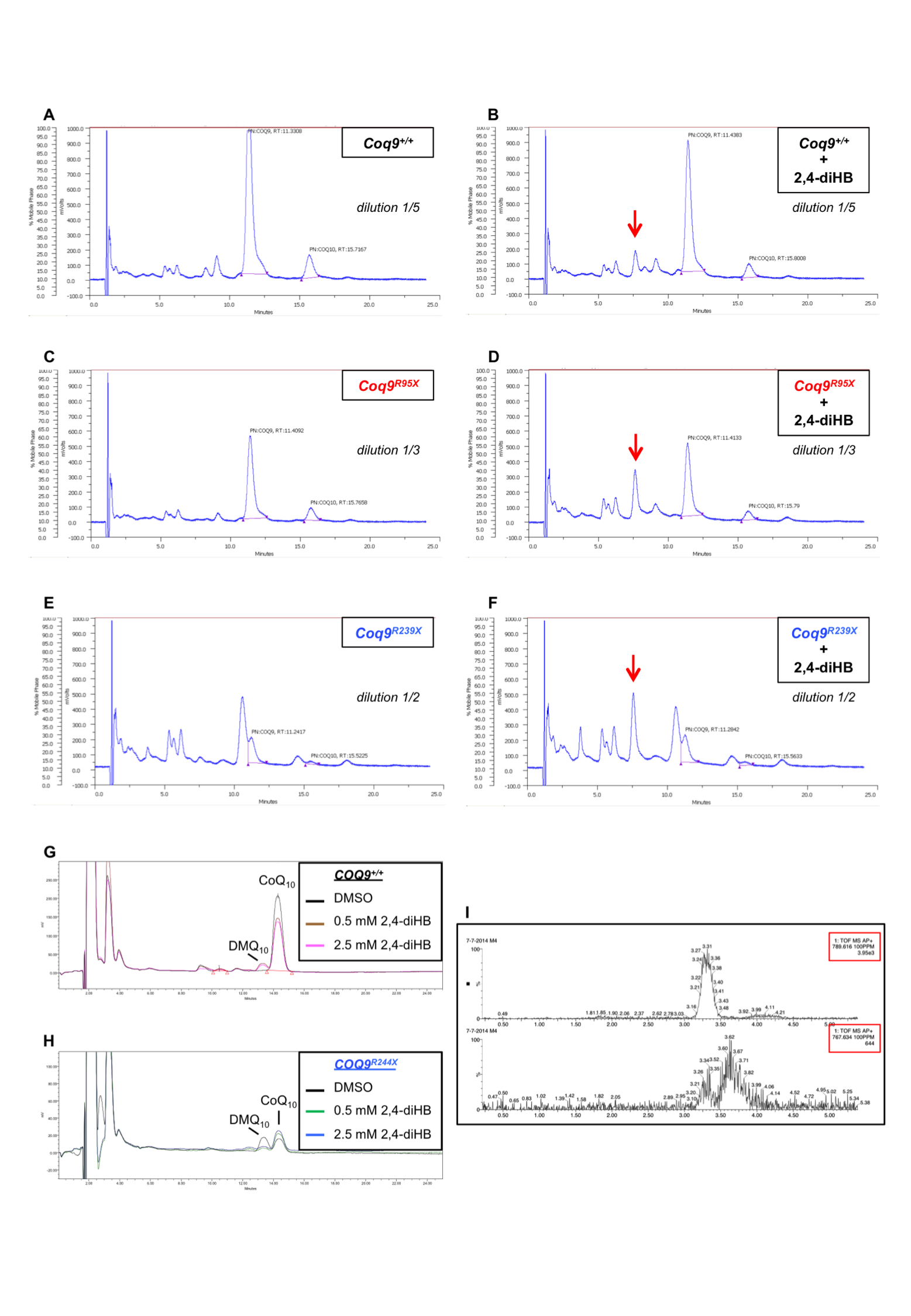

Supplement: Supplementary file 12 [file emmm0007-0670-sd12.tif]

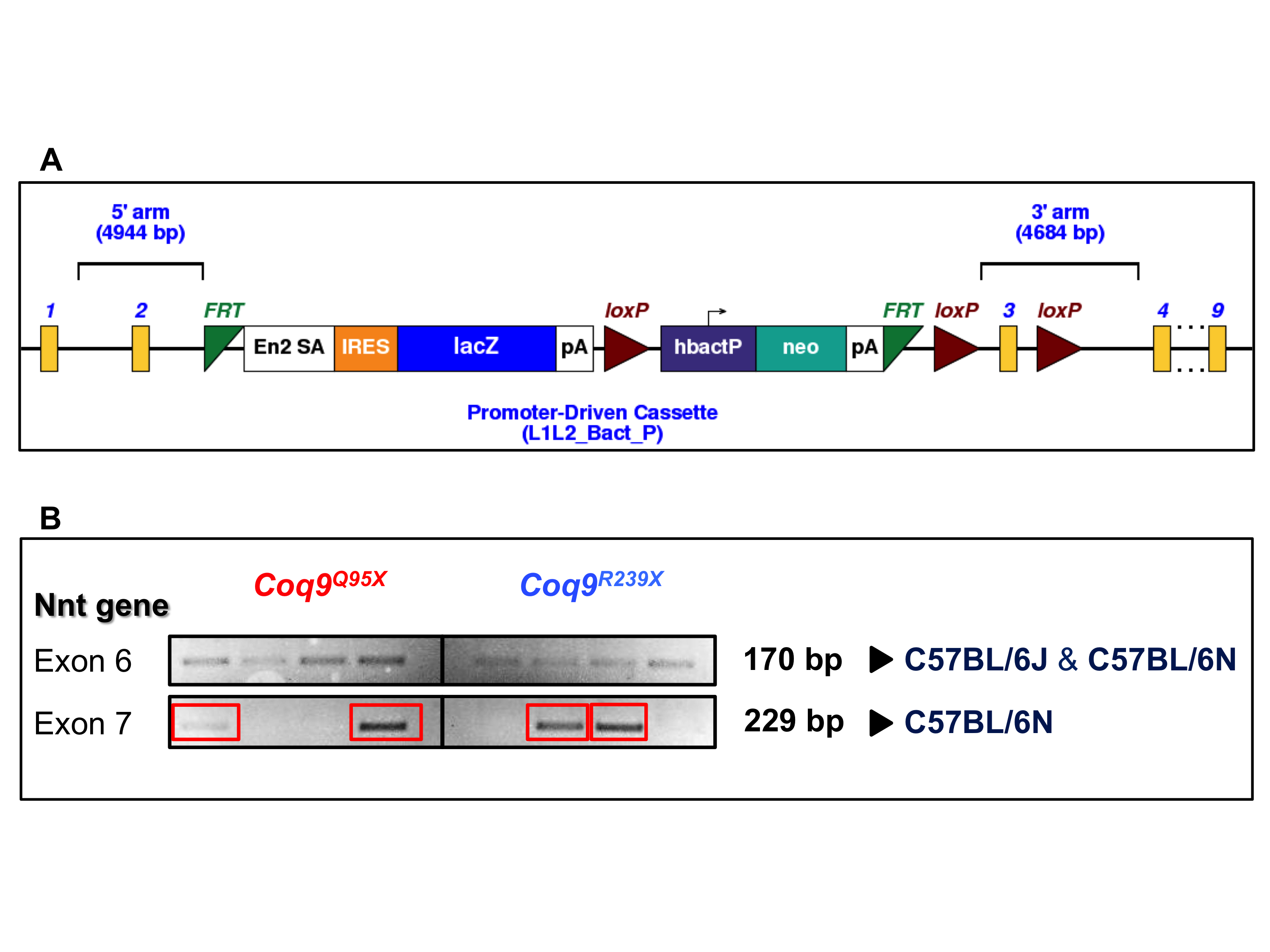

Supplement: Supplementary file 13 [file emmm0007-0670-sd13.tif]

**Figure 1. Panel B. Western blot images of COQ9 protein.**

(B) Kidney homogenate western blot of COQ9

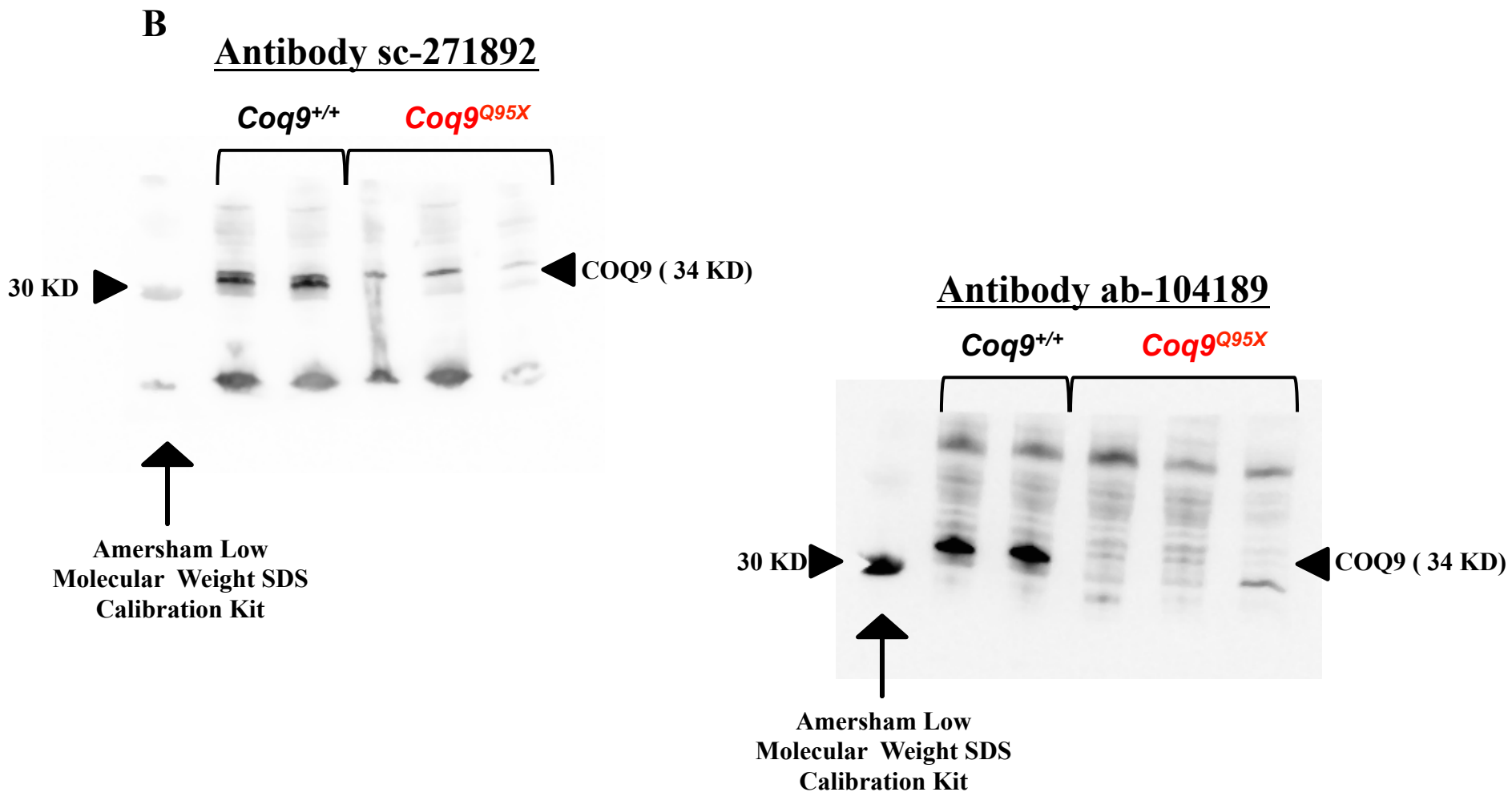

Supplement: Supplementary file 17 [file emmm0007-0670-sd17.pdf]

**Figure 2E. Truncated COQ9 protein in *Coq9*<sup>R239X</sup> mice.**

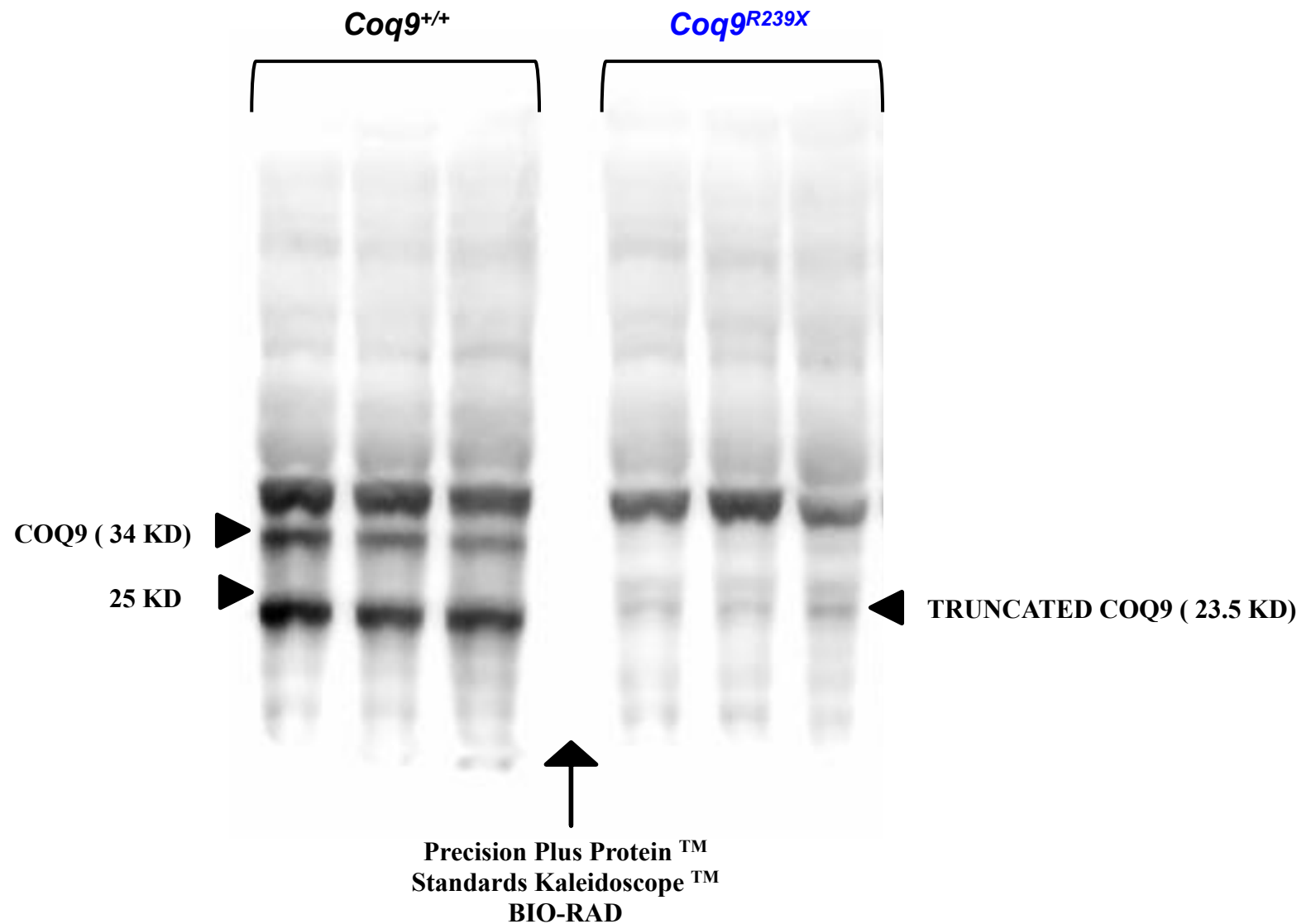

Supplement: Supplementary file 18 [file emmm0007-0670-sd18.pdf]
