# Supplementary material for: The clinical heterogeneity of coenzyme Q10 deficiency results from genotypic differences in the Coq9 gene
Source: EMBO Mol Med. 2015 Mar 23;7(5):670–87. doi: 10.15252/emmm.201404632 (PMC4492823; doi:10.15252/emmm.201404632)
Supplement: Supplementary file 14 [file emmm0007-0670-sd14.pdf]

Supplementary Information. Additional table and legends to supplementary figures.

Table of contents

A. **Supplementary tables**

B. **Supplementary figure legends**

A. Supplementary tables

Supplementary Table S1. Levels of metabolites in the urine (mg/dl).

Age	Mice Genotype	Urea	Creatinine	Albumin
3 mo.	*Coq9*$^{+/+}$	3.8 ± 2.5	49.2 ± 14.4	0.08 ± 0.05
	Coq9^{Q95X}	3.6 ± 0.3	35.8 ± 14.5	0.08 ± 0.04

Data are expressed as the mean ± SD of six experiments per group.

B. Supplementary figure legends

Figure S1. CoQ biosynthetic pathway.

CoQ_{10} is composed of a benzoquinone and a decaprenyl side chain. While the quinone ring is derived from amino acids tyrosine or phenylalanine, the isoprenoid side chain is produced by addition of isopentenyl diphosphate molecules to farnesyl diphosphate (derived from mevalonate pathway) in a reaction catalyzed by polyprenyl diphosphate synthase. After parahydroxybenzoate and polyprenyl pyrophosphate are produced, at least six enzymes (encoded by *COQ2-7*) catalyze condensation, methylation, decarboxylation, and hydroxylation reactions to synthesize CoQ. COQ9 protein interacts with hydroxylase COQ7 and it is essential for its stability and catalytic activity. n = 6 if the polyprenoid chain is nonaprenyl diphosphate (= R), the major form produced in mouse. n = 7 if the polyprenoid chain is decaprenyl diphospate (= R), the

major form produced in human. Therefore, the major proportion of CoQ in mouse is CoQ_9 while the major proportion of CoQ in human is CoQ_{10}.

Figure S2. Detection of COQ9 truncated protein en *Coq9^{R239X}*.

Representative western blot images of COQ9 protein in kidney mitochondria from *Coq9$^{+/+}$* (n=6) and *Coq9^{R239X}* mice (n=7). Antibody ab-104189 was used to map the internal sequence of the COQ9 protein. Mature form of wild-type COQ9 has a predicted molecular weight of 31.8 kDa while the mature form of the truncated version of COQ9 in *Coq9^{R239X}* mice has a predicted molecular weight of 23.5 kDa. The additional band in wild-type mice may correspond with the short isoform F6SFF5 (UniProt).

Figure S3. CoQ_{10} levels in tissue homogenates from *Coq9$^{+/+}$* and *Coq9^{Q95X}* mice.

(A-F) CoQ_{10} levels in tissue homogenates of brain (A), cerebellum (B), heart (C), kidney (D), extensor (E) and triceps surae (F) from *Coq9$^{+/+}$* and *Coq9^{Q95X}* male and female mice at 6 and 12 months of age. Data are expressed as mean ± SD. Statistical analyses were performed on 6-month-old *Coq9$^{+/+}$* mice versus 6-month-old *Coq9^{Q95X}* mice and 12-month-old *Coq9$^{+/+}$* mice versus 12-month-old *Coq9^{Q95X}* mice. *$P < 0.05$; **$P < 0.01$; ***$P < 0.001$. Student's t-test) (n=8 for each group). P = p-value.

Figure S4. Levels of COQ biosynthetic proteins in cerebrum.

(A-C) Cerebrum representative western blot and quantitation of western blot bands of COQ7 (A), ADCK3 (B) and COQ5 (C). Vdac is used as loading control. (**$P < 0.01$; ***$P < 0.001$; *Coq9^{Q95X}* and *Coq9^{R239X}* mice versus *Coq9$^{+/+}$* mice) (#$P < 0.05$; *Coq9^{Q95X}* versus *Coq9^{R239X}* mice). One-way ANOVA with a Tukey post hoc test. All values are presented as mean ± SD (*Coq9$^{+/+}$* mice n=4; *Coq9^{Q95X}* and *Coq9^{R239X}* mice n=5).

Figure S5. Decreased COQ biosynthetic proteins levels in *COQ9^{R244X}* human skin fibroblasts.

(A-D) *COQ9^{R244X}* human skin fibroblasts representative western blot and quantitation of western blot bands of COQ9 (A), COQ7 (B), ADCK3 (C) and COQ5 (D). Vinculin is used a loading control. (*$P < 0.05$; **$P < 0.01$ Controls (n=3) versus *COQ9^{R244X}* patient fibroblasts (n=1); Student's t-test) P = p-value.

Figure S6. Additional mitochondrial respiratory states of *Coq9$^{+/+}$*, *Coq9^{Q95X}* and *Coq9^{R239X}* mice.

(A-C) Kidney basal respiration (State 2) (A), resting respiration (State 4, after the addition of oligomycin) (B) and maximal uncoupler-stimulated respiration (State 3u, after the addition of FCCP) (C) from male and female *Coq9$^{+/+}$*, *Coq9^{Q95X}* and *Coq9^{R239X}* mice.

(D-E) Skeletal muscle basal respiration (State 2) (D), resting respiration (State 4, after the addition of oligomycin) (E) and maximal uncoupler-stimulated respiration (State 3u, after the addition of FCCP) (F) from male and female *Coq9$^{+/+}$*, *Coq9^{Q95X}* and *Coq9^{R239X}* mice.

All values are presented as mean ± SD. (*$P < 0.05$; **$P < 0.01$; ***$P < 0.001$; *Coq9^{Q95X}* and *Coq9^{R239X}* mice versus *Coq9$^{+/+}$* mice) (#$P < 0.05$; ##$P < 0.01$; *Coq9^{Q95X}* vs. *Coq9^{R239X}* mice). One-way ANOVA with a Tukey post hoc test. Numbers above columns indicate P-values of the one-way ANOVA test. (n=3 for each group).

Figure S7. Mitochondrial oxygen (O_2) consumption in *Coq9$^{+/+}$*, *Coq9^{Q95X}* and *Coq9^{R239X}* male mice.

(A-B) Representative O_2 consumption graphic in kidney (B) and skeletal muscle (D) from *Coq9*$^{+/+}$, *Coq9*^{Q95X} and *Coq9*^{R239X} male mice.

Figure S8. Histologic staining of tissue sections from *Coq9*$^{+/+}$and *Coq9*^{Q95X} mice at 3 months of age.

(A-D) H&E (A-B) and LFB (C-D) stains of cerebrum showing no differences between *Coq9*$^{+/+}$ (n=3) and *Coq9*^{Q95X} mice (n=3). Scale bars: 100 µm.

(E-F) PAS stain in kidney did not reveal any histologic alterations in *Coq9*^{Q95X} mice compared to *Coq9*$^{+/+}$ mice. (n=3 for each group). Scale bars: 50 µm.

(G-J) H&E stain of skeletal muscle. One *Coq9*^{Q95X} female (n=6) mice showed round shaped muscle fibers with central nuclei (H-J). Scale bars: 200 µm (G-H) and 50 µm (I-J).

Data information: H&E, Hematoxylin and eosin; LFB, Luxol fast blue; PAS, Periodic acid-Schiff.

Figure S9. Histopathology of muscle from *Coq9*$^{+/+}$ and *Coq9*^{Q95X} male mice at 6 and 12 months of age.

(A-H) Complex II (SDH) and complex IV (COX) histochemistry of *triceps surae* showing normal SDH (A-D) and COX (E-H) activity in 6- and 12-month-old *Coq9*$^{+/+}$ and *Coq9*^{Q95X} male mice.

(I-L) Gomori trichrome stain (TGM) of *triceps surae* showed no differences between 6- and 12-month-old *Coq9*$^{+/+}$ and *Coq9*^{Q95X} male mice.

(M-P) Hematoxylin and eosin (H&E) stains of triceps surae did not reveal any structural abnormality.

Scale bars: 100 µm. (n=3 for each group). Complex IV, cytochrome c

oxidase (COX); complex II, succinate dehydrogenase (SDH).

Figure S10. Immunohistochemistry of diencephalon and pons from *Coq9*$^{+/+}$ and *Coq9*^{Q95X} mice at 12 and 18 months of age.

(A-P) Anti-glial fibrillary acid protein (anti-GFAP) antibody staining of diencephalon (A-H) and pons (I-P) from 12-month and 18-month-old *Coq9*$^{+/+}$ and *Coq9*^{Q95X} mice. An overall increase of astrocytes proliferation is observed in 18-month-old *Coq9*$^{+/+}$ and *Coq9*^{Q95X} mice (n=3 for each group).

Figure S11. Histologic evaluation of kidney and heart sections from *Coq9*$^{+/+}$and *Coq9*^{Q95X} mice at 12 and 18 months of age.

(A-H) PAS stain in kidney from 12-month and 18-month-old *Coq9*$^{+/+}$ and *Coq9*^{Q95X} mice.

(I-P) TCM stain in heart from 12-month and 18-month-old *Coq9*$^{+/+}$ and Coq9^{Q95X} mice. Data information: (n=3 for each group). PAS, Periodic acid-Schiff; Masson's trichrome stain (TCM).

Figure S12. Identification of the abnormal peak in *Coq9*$^{+/+}$, *Coq9*^{Q95X} and *Coq9*^{R239X} mice treated with 2,4-diHB.

(A-F) HPLC chromatographs of 2,4-diHB treated (+2,4-diHB) and untreated (vehicle) *Coq9*$^{+/+}$, *Coq9*^{Q95X} and *Coq9*^{R239X} mice showing an abnormal peak with a retention time of 7.5 min in +2,4-diHB *Coq9* mice (B, D, F).

(G-H) HPLC chromatographs of 2,4-diHB treated (+2,4-diHB) and untreated (vehicle) *COQ9*^{R244X} skin fibroblasts.

(I) Mass spectrometric analysis of the abnormal peak indicates that its molecular ion [M + H]+ corresponds to 767.634 [M + H]+ and 789.616 [M + Na]+.

Figure S13. Generation of *Coq9* Q95X mouse model.

(A) Schematic representation of the promoter driven vector for the targeting of the *Coq9* gene inserted between exons 2 and 3. The targeting vector contains a targeting cassette with a neo-resistance gene that is driven by the β-actin promoter. This allows targeting of all genes, irrespective of their expression status in mouse ES cells. Following removal of the floxed region, a transcript is predicted to produce a truncated protein product that may be subject to non-sense mediated decay (NMD). The predicted structure of the gene transcript contains only exons 1 and 2 and the predicted protein has the following sequence:

MAATAAVSGVLGRLGWRLLQLRCLPVARCRPALVPRAFHTAVGFRSSEEQKQ
QPPHSSSQQHSETQGPEFSRPPPSPWVSPAQQPACLGAMAVS

(B) Genomic PCR specific for exons 6-7 of the *Nnt* gene. Nnt gene is complete in the sub-strain C57/BL6N while presents a deletion in exon 7 in the sub-strain C57/BL6J. Therefore, exons 6 and 7 can be amplified in the sub-strain C57/BL6N while in the sub-strain C57/BL6J only exon 6 can be amplified. The results show that the two genetically modified mouse models, $Coq9^{Q95X}$ (n=4) and $Coq9^{R239X}$ mice (n=4), have a mix of C57BL/6N and C57BL/6J genetic background.
