# Supplementary material for: The clinical heterogeneity of coenzyme Q10 deficiency results from genotypic differences in the Coq9 gene
Source: EMBO Mol Med. 2015 Mar 23;7(5):670–87. doi: 10.15252/emmm.201404632 (PMC4492823; doi:10.15252/emmm.201404632)

Figure S4. Panel A. Levels of COQ biosynthetic proteins in cerebrum

(A) Cerebrum western blot of COQ7.

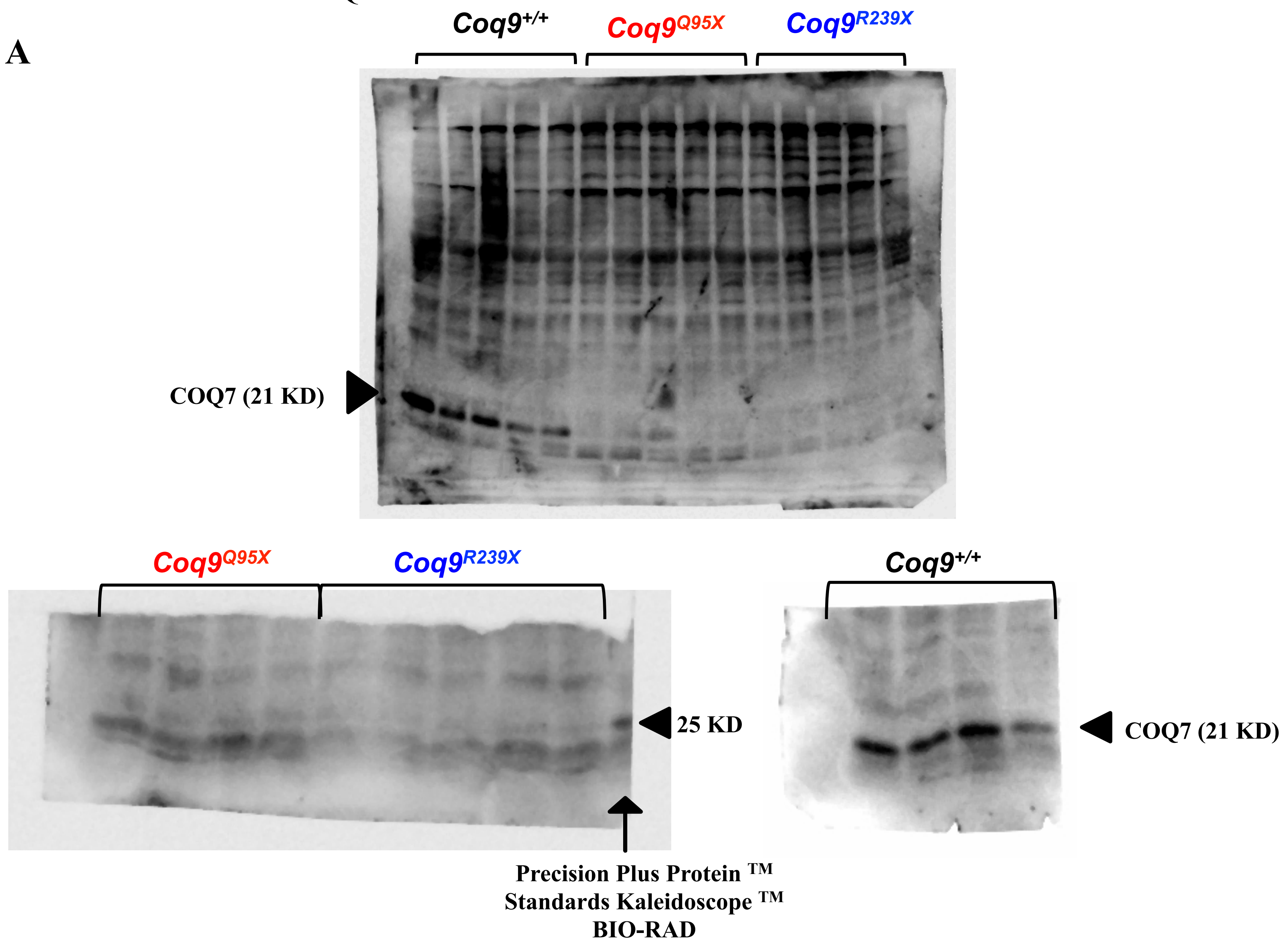

Figure S4. Panel B. Levels of COQ biosynthetic proteins in cerebrum

(B) Cerebrum western blot of ADCK3.

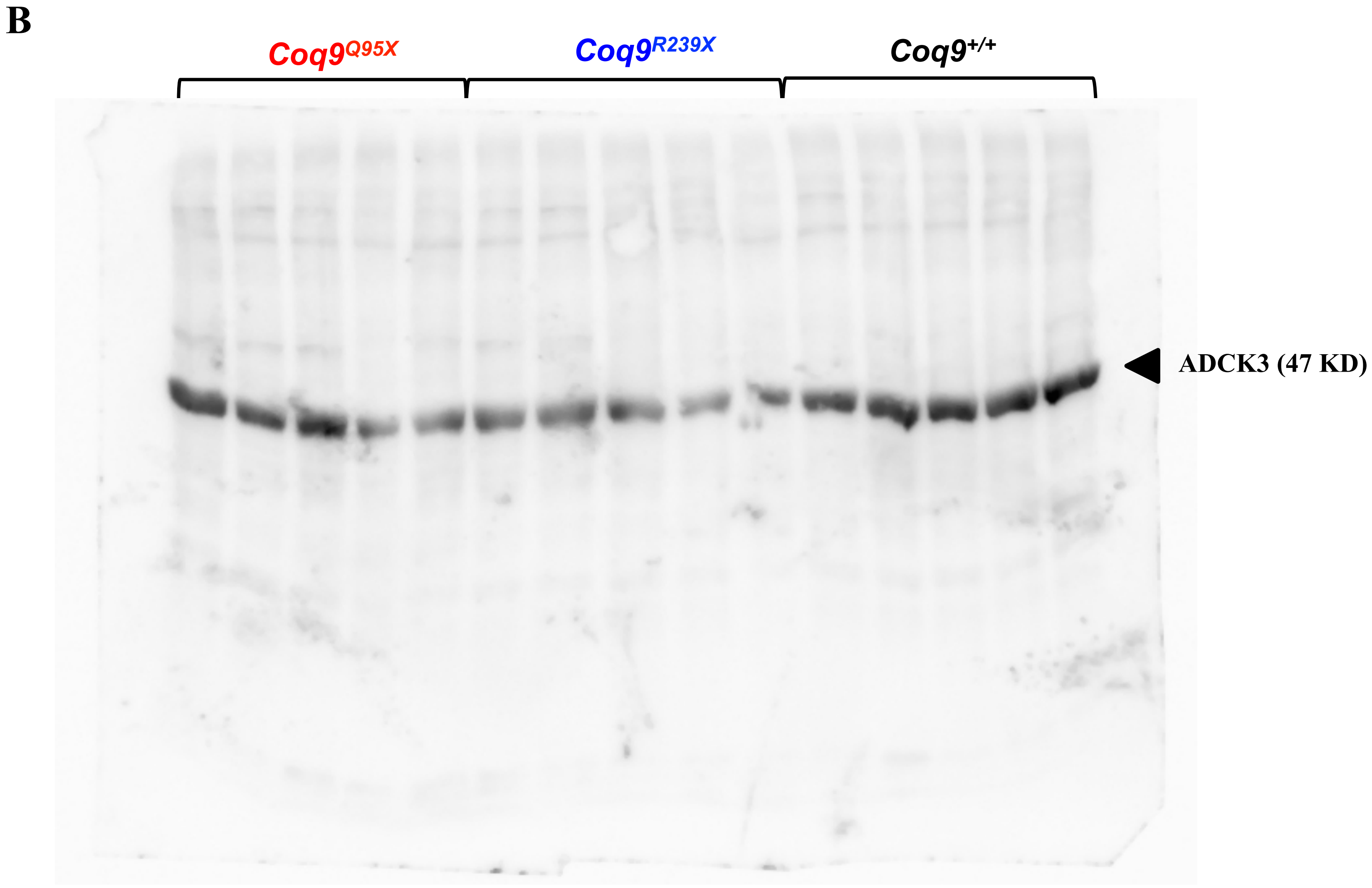

Figure S4. Panel C. Levels of COQ biosynthetic proteins in cerebrum

(C) Cerebrum western blot of COQ5.

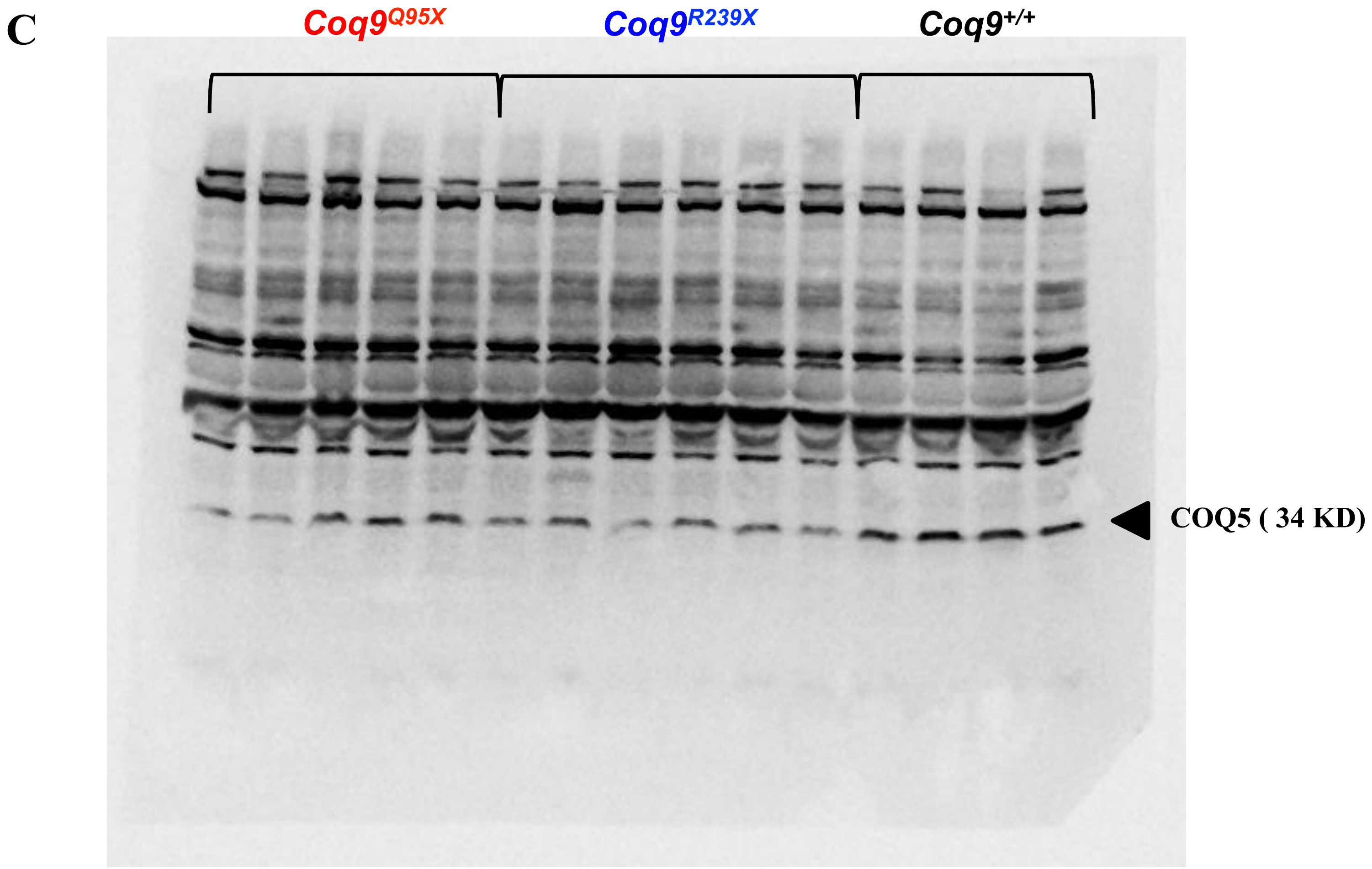

Supplement: Supplementary file 15 [file emmm0007-0670-sd15.pdf]
