# Supplementary material for: The clinical heterogeneity of coenzyme Q10 deficiency results from genotypic differences in the Coq9 gene
Source: EMBO Mol Med. 2015 Mar 23;7(5):670–87. doi: 10.15252/emmm.201404632 (PMC4492823; doi:10.15252/emmm.201404632)
Supplement: Supplementary file 16 [file emmm0007-0670-sd16.pdf]

The clinical heterogeneity of coenzyme Q10 deficiency results from genotypic differences in the Coq9 gene

Marta Luna-Sánchez, Elena Díaz -Casado, Emanuele Barca, Miguel Tejada, Angeles Montilla-GarcÌa, Enrique J.Cobos, Germaine Escames, Dario Acuña -Castroviejo, Catarina M.Quinzii and Luis Lopez

Corresponding author: Luis Lopez, University of Granada

Review timeline:

Submission date:	31 October 2014
Editorial Decision:	28 November 2014
Revision received:	03 February 2015
Editorial Decision:	19 February 2015
Revision received:	24 February 2015
Accepted:	26 February 2015

Transaction Report:

(Note: With the exception of the correction of typographical or spelling errors that could be a source of ambiguity, letters and reports are not edited. The original formatting of letters and referee reports may not be reflected in this compilation.)

Editor: Céline Carret

1st Editorial Decision 28 November 2014

Thank you for the submission of your manuscript to EMBO Molecular Medicine. We have now heard back from the referees we asked to evaluate your manuscript. Although they find the study to be of potential interest, they also raise a number of concerns that must be addressed in full in the next version of your article.

As you will see from the comments below, both referees require experimental evidence for the truncated COQ9 protein expression and request a better explanation of the human disease relevance. Should you be able to address this and all remaining referees' criticisms in full, we would be willing to consider a revised manuscript.

Please note that it is EMBO Molecular Medicine policy to allow only a single round of revision and that, as acceptance or rejection of the manuscript will depend on another round of review, your responses should be as complete as possible.

EMBO Molecular Medicine has a "scooping protection" policy, whereby similar findings that are published by others during review or revision are not a criterion for rejection. Should you decide to submit a revised version, I do ask that you get in touch after three months if you have not completed it, to update us on the status.

Please also contact us as soon as possible if similar work is published elsewhere. If other work is published we may not be able to extend the revision period beyond three months.

I look forward to receiving your revised manuscript.

***** Reviewer's comments *****

Referee #1 (Comments on Novelty/Model System):

The introduction does not make sufficiently clear the importance of the Coq9q95X mutation with respect to human disease. Does this also relate to a known pathogenic mutation in humans. If so how common is it and does it match the phenotype exhibited in their mouse model. Without this, although interesting and well conducted, this referee does not believe that EMBO Mol. Med.is the appropriate journal for this manuscript.

Referee #1 (Remarks):

The experiments are many and diverse. All appear to have been carried out well and have generated good quality data. The phenomenon of unpredictable onset and varied severity in humans with a primary CoQ10 deficiency is intriguing. It is not uncommon for mitochondrial diseases and is worthy of investigation.

Neither of the antibodies that are used to look for presence of COQ9 would recognize the fragment of protein that Q95X mice would produce. Although this would confirm that no full length protein is present it would not indicate whether a truncated but stable fragment was present. The authors use LCMSMS to show apparently detectable, residual amounts of the truncated protein but at much lower levels than in WT mice. The COQ9 westerns are rather over-exposed so that the contrast is a little too stark. This makes it hard to see any context. If accepted I would recommend a different exposure be presented.

The consequences of the 2 mutations tested show differences in the levels of CoQ9 as shown in different tissues as a % of control (fig 3). This is an interesting observation based on the similar lack of CoQ9 in each case.

Low levels of CoQ9 mRNA suggest degradation by NMD. This is a common mechanism to dispose of transcripts that cause dominant negative or deleterious gain of function proteins. Here with the exception of CoQ9, the changes in pathway related mRNAs are generally unaffected and even those with statistical significance are not altered by more than 25% in all cases except one. Indeed where protein levels have been looked at these do not correlate well with transcript levels.
The observation that activity assays only show a difference in females is curious but appears robust.

The histochemistry has generated good crisp data but this referee is not convinced that there is a significant difference in the proportion of COX negative fibres in the mutant Q95X mouse compared to control at 18 months. A better way to assess any change in Type I to type II fibres would be to specifically stain for them as is routinely done (Myofibrillar ATPase activity demonstrated at pH 9.5, after pre-incubatioin at pH 4.3 or pH 4. 6, allows the differentiation of type 1 (slow-twitch oxidative), type 2A (fast-twitch oxidative/glycolytic) and type 2B (fast-twitch glycolytic fibres)). The abstract is a little disingenuous to say that there is "a shift from type I to type II fibers", when this has not been specifically tested for and is only described as 'suggestive' in the text.

Minor points

There are points where the English is not quite correct, although this does not affect the reader's understanding of the text.
e.g. "As described in the Coq9R239X, by postnatal day 21, also the Coq9Q95X mice lost their body hair. . . . . ." This would have been better as 'As described in the Coq9R239X, by postnatal day 21 the Coq9Q95X mice had also lost their body hair. . . . .'

more importantly -
"Although COQ9 was clearly detected in the wild type samples, none of its peptides were observed in Coq9Q95X mice (Fig 1C), demonstrating that the COQ9 protein was completely absent in Coq9Q95X mice."
In this instance the sentence appears to contradict the earlier one saying that the peptide that would be found in wt and show an arrow indicating a small peak suggesting that the protein had been made but was degraded.

Referee #2 (Remarks):

In this study the authors probe the functional roles of the Coq9 polypeptide. They use a knock-in mouse model and study the genotype-phenotype of two homozygous mice - one harboring a Coq9Q95X mutation (stop codon is near the amino terminal end) and one harboring a Coq8R239X mutation (stop codon is located within the carboxyl terminal domain). The phenotypes of the two mice are quite different, with the Coq9Q95X mouse showing only mild deficiency in coenzyme Q, late onset mild mitochondrial myopathy and a detrimental response to treatment with a potential bypass aromatic ring precursor, 2,4-dihydroxybenzoic acid. In contrast, the Coq9R239X mouse shows a severe and widespread CoQ deficiency that leads to fatal encephalomyopathy, but when treated with 2,4-dihydroxybenzoic acid the mice do respond with increased CoQ content.

The findings are interesting because they shed light on the puzzling and diverse phenotypes noted in human patients with primary deficiencies in coenzyme Q biosynthesis. The findings reported suggest that the Coq9 polypeptide with the stop codon located near to the C-terminal end of the Coq9 polypeptide produces a more severe phenotype because the Coq9R239X polypeptide destabilizes the other Coq polypeptides. The data presented argue for the importance of a multiprotein complex for CoQ biosynthesis in a mammalian model, and provide new insights into possible diverse outcomes observed in human CoQ deficient patients.

The following suggestions are made to help clarify the manuscript and correct some aspects of the presentation.

1. When the Coq9R239X mouse was originally reported (Garcia-Corzo et al., 2013) the only antibody available at that time was the antibody recognizing the carboxyl terminal part of Coq9. Thus western blots reported for Coq9R239X showed only the absence of a Coq9 protein signal. Now that the authors have an antibody that recognizes the middle of the Coq9 polypeptide sequence, it is important to show that the Coq9R239X polypeptide is actually present. This is important because the authors claim that a missing Coq9Q95X polypeptide (as shown in Figure 1) is less toxic than a disruptive CoqR239X polypeptide. Thus it is incumbent upon the authors to verify the presence of the Coq9R239X polypeptide, either with Western blot analyses similar to those in Fig 1B, or with mass spectrometry proteomic analyses (Fig 1C).

2. Supplemental Figure 1E
The authors should indicate that seven (n-3) IPP molecules are utilized at the decaprenyl diphosphate synthase step to produce the decaprenyl diphosphate product. The structure of the decaprenyl diphosphate molecule is not correct. Also, the legend refers to geranylgeranyl diphosphate, yet it is farnesyl diphosphate which is depicted in the figure. Since the authors are monitoring CoQ9 content in their mouse model, it would help the reader to note that the major biosynthetic product in mouse is CoQ9.

3. In the introduction the authors note that Coq2 mediates the conjugation of the benzoquinone ring to the side chain. However, Coq2 does not act on the benzoquinone ring, but rather on aromatic ring precursors.

4. The authors note in the introduction that there are five major clinical presentations of coenzyme Q10 deficiency. However, mutations in COQ2 were also reported to be associated with multiple system atrophy (NEJM 2013) by the Multiple-System Atrophy Research Collaboration. This adds

yet another clinical presentation that has been associated with defects in coenzyme Q biosynthesis.

5. The legend to Figure 2E (supplement) does not provide an adequate description of the figure for the non-expert.

1st Revision - authors' response 03 February 2015

Referee #1

We appreciate the insightful comments of this reviewer.

1. *The introduction does not make sufficiently clear the importance of the Coq9q95X mutation with respect to human disease. Does this also relate to a known pathogenic mutation in humans. If so how common is it and does it match the phenotype exhibited in their mouse model. Without this, although interesting and well conducted, this referee does not believe that EMBO Mol. Med.is the appropriate journal for this manuscript.*

   $Coq9^{Q95X}$ does not relate to any mutation in humans; in fact, only one patient with mutations in COQ9 has been described so far, and he carried the R244X mutation (cited in page 4) (Duncan et al, 2009), homologue of the R239X of our knock in mouse. However, we think that *in vivo* comparison of two different mutations in the *Coq9* gene is the best option to address the genetically and clinically heterogeneity of human CoQ_{10} deficiency (Emmanuele et al, 2012), since the only other viable mouse model of human primary CoQ_{10} deficiency described, the *$Pdss2^{kd/kd}$* (Peng et al, 2008) (which carry a spontaneous mutation in *Pdss2*, not homologue to any of the reported human mutation) does not actually recapitulate the human disease. The others mouse models are conditional tissue-specific and, therefore, they do not have wide-spread CoQ deficiency, and the non-conditional mutations are embryonically lethal. In this context, we show that the efficiency of NMD may determine the clinical phenotype. In this regard, we have made new experiments to prove that the low Coq9 mRNA detected in mutant mice is the result of NMD (Table 1), as we suggested in the previous version. This is common to other genetic diseases and, therefore, increases the importance of the results for human diseases from a mechanistic and therapeutic point of views. We have incorporated this information in the results (page 7, first paragraph, and table 1), discussion (page 13, first paragraph) and methods (pages 17-18) with new references.

   On the other hand, our results are not only of intellectual interest, but have therapeutic implications, since the two mouse models respond differently to the administration of 2,4-diHB, as we wrote in the discussion.

2. *The experiments are many and diverse. All appear to have been carried out well and have generated good quality data. The phenomenon of unpredictable onset and varied severity in humans with a primary CoQ10 deficiency is intriguing. It is not uncommon for mitochondrial diseases and is worthy of investigation.*

   We thank the reviewer for this positive comment. It is important to remark that the unpredictable phenotype is not uncommon for mitochondrial diseases and that was one of the reasons to develop our study comparing two mouse models with different mutations in the same gene.

3. *Neither of the antibodies that are used to look for presence of COQ9 would recognize the fragment of protein that Q95X mice would produce. Although this would confirm that no full length protein is present it would not indicate whether a truncated but stable fragment was present. The authors use LCMSMS to show apparently detectable, residual amounts of the truncated protein but at much lower levels than in WT mice. The COQ9 westerns are rather*

over-exposed so that the contrast is a little too stark. This makes it hard to see any context. If accepted I would recommend a different exposure be presented.

We apologize if the arrows of figure 1 have given the impression that detectable levels of COQ9 truncated protein were found in *Coq9*^{Q95X} mice. The arrows were moved to the right during the file conversion to pdf and we have now corrected these errors. As we wrote on page 5, we were unable to detect any COQ9 peptide (including QQPPHSSSQQHSETQGPEFSRPPR) in mitochondrial samples from *Coq9*^{Q95X} mice. Moreover, we have changed the images of WB in figure 1 to show them with a different exposure.

4. *The consequences of the 2 mutations tested show differences in the levels of CoQ9 as shown in different tissues as a % of control (fig 3). This is an interesting observation based on the similar lack of CoQ9 in each case.*

We thank the reviewer for the positive comment. Our results show that a truncated version of the COQ9 protein in *Coq9*^{R239X} mice induces instability of the CoQ multiprotein complex leading to a further decrease of CoQ levels. Therefore, the truncated version of the COQ9 protein in *Coq9*^{R239X} mice is responsible for the lower CoQ levels in these mice compared to *Coq9*^{Q95X} mice. We based this interpretation on the differences found in the Coq9 transcript levels between *Coq9*^{R239X} and *Coq9*^{Q95X} mice. To be more precise in our conclusions, we have added a new figure (Supplementary Fig. E2) of two representative western-blot showing the presence of the truncated protein in *Coq9*^{R239X} mice using an antibody against the internal sequence of the protein. We have commented this result in page 5 (second paragraph) and page 13 (second paragraph), adding two additional references. We have also added a sentence in the methods (page 20).

5. *Low levels of CoQ9 mRNA suggest degradation by NMD. This is a common mechanism to dispose of transcripts that cause dominant negative or deleterious gain of function proteins. Here with the exception of CoQ9, the changes in pathway related mRNAs are generally unaffected and even those with statistical significance are not altered by more than 25% in all cases except one. Indeed where protein levels have been looked at these do not correlate well with transcript levels.*

As the reviewer noted, Coq9 mRNA is degraded by NMD in both mouse models. However, we found differences in the level of Coq9 mRNA degradation between the two mouse models: the results in *Coq9*^{Q95X} mice show total degradation of Coq9 mRNA in brain, kidney and muscle, while *Coq9*^{R239X} mice only show partial degradation of Coq9 mRNA in brain and kidney. The residual Coq9 mRNA in *Coq9*^{R239X} mice produces a truncated version of the COQ9 protein (Supplementary Fig. E2) that makes the CoQ multiprotein complex unstable. Studies in yeast previously showed that when a mutation in a COQ protein induces instability of the CoQ multiprotein complex, the majority of the components of this complex are partially degraded (Tran & Clarke, 2007). This explains the disparity in the correlation between transcript levels and protein levels.

6. *The observation that activity assays only show a difference in females is curious but appears robust.*

We agree with the reviewer's comment. As we mention in the discussion, it is interesting that gender differences has been also found in other mouse models of mitochondrial diseases.

7. *The histochemistry has generated good crisp data but this referee is not convinced that there is a significant difference in the proportion of COX negative fibres in the mutant Q95X mouse compared to control at 18 months. A better way to assess any change in Type I to type II fibres would be to specifically stain for them as is routinely done (Myofibrillar ATPase activity demonstrated at pH 9.5, after pre-incubatioin at pH 4.3 or pH 4. 6, allows the differentiation of type 1 (slow-twitch oxidative), type 2A (fast-twitch oxidative/glycolytic) and type 2B (fast-twitch glycolytic fibres)). The abstract is a little disingenuous to say that there is "a shift from type I to type II fibers", when this has not been specifically tested for and is only described as 'suggestive' in the text.*

   We agree with the reviewer's comment and we have deleted this statement in the "abstract" and "the paper explained" sections.

Minor points

There are points where the English is not quite correct, although this does not affect the reader's understanding of the text. e.g. "As described in the Coq9R239X, by postnatal day 21, also the Coq9Q95X mice lost their body hair. . . . . ." This would have been better as 'As described in the Coq9R239X, by postnatal day 21 the Coq9Q95X mice had also lost their body hair. . . . .'

We apologize for any misuse of the English language. We have carefully reviewed the manuscript and made punctual modifications, including the indicated sentence.

more importantly - "Although COQ9 was clearly detected in the wild type samples, none of its peptides were observed in Coq9Q95X mice (Fig 1C), demonstrating that the COQ9 protein was completely absent in Coq9Q95X mice." In this instance the sentence appears to contradict the earlier one saying that the peptide that would be found in wt and show an arrow indicating a small peak suggesting that the protein had been made but was degraded.

We apologize if the arrows of figure 1 have given the impression that detectable levels of COQ9 truncated protein were found in *Coq9*^{Q95X} mice. The arrows were moved to the right during the file conversion to pdf and we have now corrected these errors. As we wrote on page 5, we were not able to detect any COQ9 peptide (including QQPPHSSSQQHSETQGPEFSRPPR) in mitochondrial samples from *Coq9*^{Q95X} mice. Therefore, this sentence accurately describes our LC-MS/MS results.

Referee #2

We thank the general positive comments of this reviewer, as well as the useful recommendations to improve the manuscript.

1. *The findings are interesting because they shed light on the puzzling and diverse phenotypes noted in human patients with primary deficiencies in coenzyme Q biosynthesis. The findings reported suggest that the Coq9 polypeptide with the stop codon located near to the C-terminal end of the Coq9 polypeptide produces a more severe phenotype because the Coq9R239X polypeptide destabilizes the other Coq polypeptides. The data presented argue for the importance of a multiprotein complex for CoQ biosynthesis in a mammalian model, and provide new insights into possible diverse outcomes observed in human CoQ deficient patients.*

We thank the reviewer for this comment. We completely agree with this point of view.

2. *When the Coq9R239X mouse was originally reported (Garcia-Corzo et al., 2013) the only antibody available at that time was the antibody recognizing the carboxyl terminal part of Coq9. Thus western blots reported for Coq9R239X showed only the absence of a Coq9 protein signal. Now that the authors have an antibody that recognizes the middle of the Coq9 polypeptide sequence, it is important to show that the Coq9R239X polypeptide is actually present. This is important because the authors claim that a missing Coq9Q95X polypeptide (as shown in Figure 1) is less toxic than a disruptive CoqR239X polypeptide. Thus it is incumbent upon the authors to verify the presence of the Coq9R239X polypeptide, either with Western blot analyses similar to those in Fig 1B, or with mass spectrometry proteomic analyses (Fig 1C).*

   We thank the reviewer for suggesting this additional experiment, which makes the results and conclusions more robust. We have added a new figure (Supplementary Fig. E2) of two representative WB showing the presence of the truncated protein in *Coq9*^{R239X} mice using an antibody against the internal sequence of the protein. We have commented this result in page 5 and page 13 (second paragraph), adding two additional references. We have also added a sentence in the methods (page 20).

3. *Supplemental Figure 1E. The authors should indicate that seven (n-3) IPP molecules are utilized at the decaprenyl diphosphate synthase step to produce the decaprenyl diphosphate product. The structure of the decaprenyl diphosphate molecule is not correct. Also, the legend refers to geranylgeranyl diphosphate, yet it is farnesyl diphosphate which is depicted in the figure. Since the authors are monitoring CoQ9 content in their mouse model, it would help the reader to note that the major biosynthetic product in mouse is CoQ9.*

   We apologize for the mistakes in the CoQ biosynthetic pathway, which we have corrected. We have also made some modifications in the figure and its legend to explain that the major form of CoQ in mouse is CoQ_9 while in human is CoQ_{10}.

4. *In the introduction the authors note that Coq2 mediates the conjugation of the benzoquinone ring to the side chain. However, Coq2 does not act on the benzoquinone ring, but rather on aromatic ring precursors.*

   We apologize for this mistake in the text. We have corrected it.

5. *The authors note in the introduction that there are five major clinical presentations of coenzyme Q10 deficiency. However, mutations in COQ2 were also reported to be associated with multiple system atrophy (NEJM 2013) by the Multiple-System Atrophy Research Collaboration. This adds yet another clinical presentation that has been associated with defects in coenzyme Q biosynthesis.*

   We thank the reviewer for reminding us of this additional clinical presentation associated to mutations in *COQ2*. We have added multiple-system atrophy to the clinical presentations (page 4).

6. *The legend to Figure 2E (supplement) does not provide an adequate description of the figure for the non-expert.*

We thank the reviewer for pointing out the lack of an adequate description of this figure. We have added new information about this figure in its legend, which is in this revised version the "Supplementary Figure E13".

Literature cited:

Duncan AJ, Bitner-Glindzicz M, Meunier B, Costello H, Hargreaves IP, Lopez LC, Hirano M, Quinzii CM, Sadowski MI, Hardy J et al (2009) A nonsense mutation in COQ9 causes autosomal-recessive neonatal-onset primary coenzyme Q10 deficiency: a potentially treatable form of mitochondrial disease. Am J Hum Genet 84: 558-566

Emmanuele V, Lopez LC, Berardo A, Naini A, Tadesse S, Wen B, D'Agostino E, Solomon M, DiMauro S, Quinzii C et al (2012) Heterogeneity of coenzyme Q10 deficiency: patient study and literature review. Arch Neurol 69: 978-983

Peng M, Falk MJ, Haase VH, King R, Polyak E, Selak M, Yudkoff M, Hancock WW, Meade R, Saiki R et al (2008) Primary coenzyme Q deficiency in Pdss2 mutant mice causes isolated renal disease. PLoS Genet 4: e1000061

Tran UC, Clarke CF (2007) Endogenous synthesis of coenzyme Q in eukaryotes. Mitochondrion 7 Suppl: S62-S71

2nd Editorial Decision 19 February 2015

Thank you for the submission of your revised manuscript to EMBO Molecular Medicine. We have now received the enclosed reports from the referees that were asked to re-assess it. As you will see the reviewers are now globally supportive and I am pleased to inform you that we will be able to accept your manuscript pending the following final amendments:

1) As you can see both referees noted some typos and little mistake that should be corrected

Please submit your revised manuscript within two weeks. I look forward to seeing a revised form of your manuscript as soon as possible.

***** Reviewer's comments *****

Referee #1 (Comments on Novelty/Model System):

The model is still in mice with different consequences to humans with the mutations. Work is well done and authors have addressed the reviewers' comments.

Referee #1 (Remarks):

The Authors have responded in detail to the comments made by the reviewers.

Page 4 Line 3 - Japanese should have a capital letter.
Page 4 - "mouse, which is homologue to the human R244X mutation"; grammar needs correction.

'At the contrary' is used a few times and is not a standard phrase - 'in contrast' might be better.

Page 14 "The unbalance of the CoQ" should read 'imbalance'

Page 22 - methods -"ubiquinol-Cytochrome C Reductase C" should be italicized lower case 'c' after cytochrome.

Referee #2 (Comments on Novelty/Model System):

The authors have carefully addressed and responded to the concerns raised by the reviewers. A few minor corrections are noted:

Referee #2 (Remarks):

The authors have carefully addressed and responded to the concerns raised by the reviewers. A few minor corrections are noted:
1. Supplemental Figure 1 is much improved. The depiction of the "n" number of IPP molecules required would be more clear if the n was moved in front of the IPP structure, and the brackets were removed.
2. On page 3, the authors refer to COQ1 as synthesizing the polyprenyl-diphosphate tail. Because they are analyzing the process in mice, they should instead refer to the Pdss1 and Pdss2 gene products, which are responsible for the tail synthesis in mice and humans.
3. Change "lost" to "loss" in the sentence on page 5: To check whether the premature termination of the COQ9 protein induces the complete lost of the protein,...
4. Figure E2 shows the truncated form of Coq9 polypeptide present in the Coq9R239X homozygous mutant. Is this truncated form absent in the Coq9Q95X mouse? If a larger segment of the immunoblot was shown in Figure 1, the authors could support their claim that this short form was not observed with the same antibody that showed the truncated form was present in the Coq9R239X mutant.
5. Page 26: "Acquity ultra carrying out liquid chromatography"? Do the authors mean to refer to the Acquity Ultra-Performance liquid chromatography?

2nd Revision - authors' response 24 February 2015

Referee #1

We appreciate the insightful suggestions of this reviewer.

1. *Page 4 Line 3 - Japanese should have a capital letter.*

   We have corrected this typo.

2. *Page 4 - "mouse, which is homologue to the human R244X mutation"; grammar needs correction.*

   We have remade this sentence as follow: “To better understand the pathophysiologic consequences of primary CoQ10 deficiency, we recently generated a mouse model carrying

a homozygous mutation in Coq9 gene (R239X, Coq9R239X). This mutation is homologue to the human R244X mutation (Duncan et al, 2009). Coq9R239X mice showed wide spread CoQ deficiency (Garcia-Corzo et al, 2013) and their characterization demonstrated that:"

3. *'At the contrary' is used a few times and is not a standard phrase - 'in contrast' might be better.*

   We have made this change in the abstract (page 2) and in page 5.

4. *Page 14 "The unbalance of the CoQ" should read 'imbalance'*

   We have corrected it.

5. *Page 22 - methods -"ubiquinol-Cytochrome C Reductase C" should be italicized lower case 'c' after cytochrome.*

   We have corrected it.

Referee #2

We appreciate the insightful suggestions of this reviewer.

1. *Supplemental Figure 1 is much improved. The depiction of the "n" number of IPP molecules required would be more clear if the n was moved in front of the IPP structure, and the brackets were removed.*

   We have made the suggested change in the figure.

2. *On page 3, the authors refer to COQ1 as synthesizing the polyprenyl-diphosphate tail. Because they are analyzing the process in mice, they should instead refer to the Pdss1 and Pdss2 gene products, which are responsible for the tail synthesis in mice and humans.*

   We thank the reviewer for this recommendation. We have made the suggested change.

3. *Change "lost" to "loss" in the sentence on page 5: To check whether the premature termination of the COQ9 protein induces the complete lost of the protein...*

   We have made this correction.

4. *Figure E2 shows the truncated form of Coq9 polypeptide present in the Coq9R239X homozygous mutant. Is this truncated form absent in the Coq9Q95X mouse? If a larger segment of the immunoblot was shown in Figure 1, the authors could support their claim that this short form was not observed with the same antibody that showed the truncated form was present in the Coq9R239X mutant.*

   A larger segment of the immunoblot is now shown in Figure 1.

5. *Page 26: "Acquity ultra carrying out liquid chromatography"? Do the authors mean to refer to the Acquity Ultra-Performance liquid chromatography?*

   We have made the suggested change.
