# Supplementary material for: The clinical heterogeneity of coenzyme Q10 deficiency results from genotypic differences in the Coq9 gene
Source: EMBO Mol Med. 2015 Mar 23;7(5):670–87. doi: 10.15252/emmm.201404632 (PMC4492823; doi:10.15252/emmm.201404632)

Figure 5. Panel A. Levels of Coq biosynthetic proteins

(A) Kidney western blot of COQ7 and VDAC1 as a loading control.

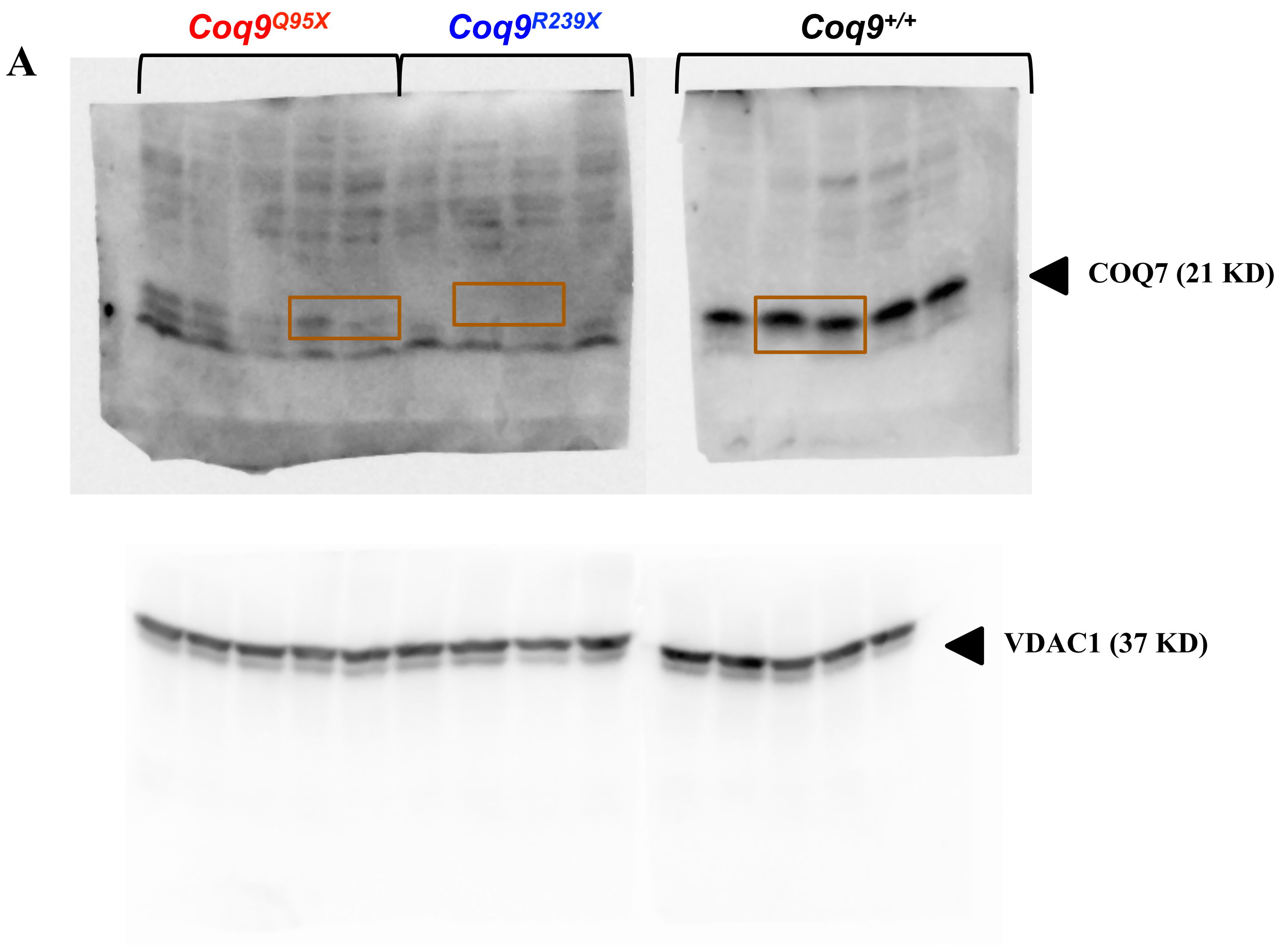

Figure 5. Panel B. Levels of Coq biosynthetic proteins

(B) Kidney western blot of ADCK3.

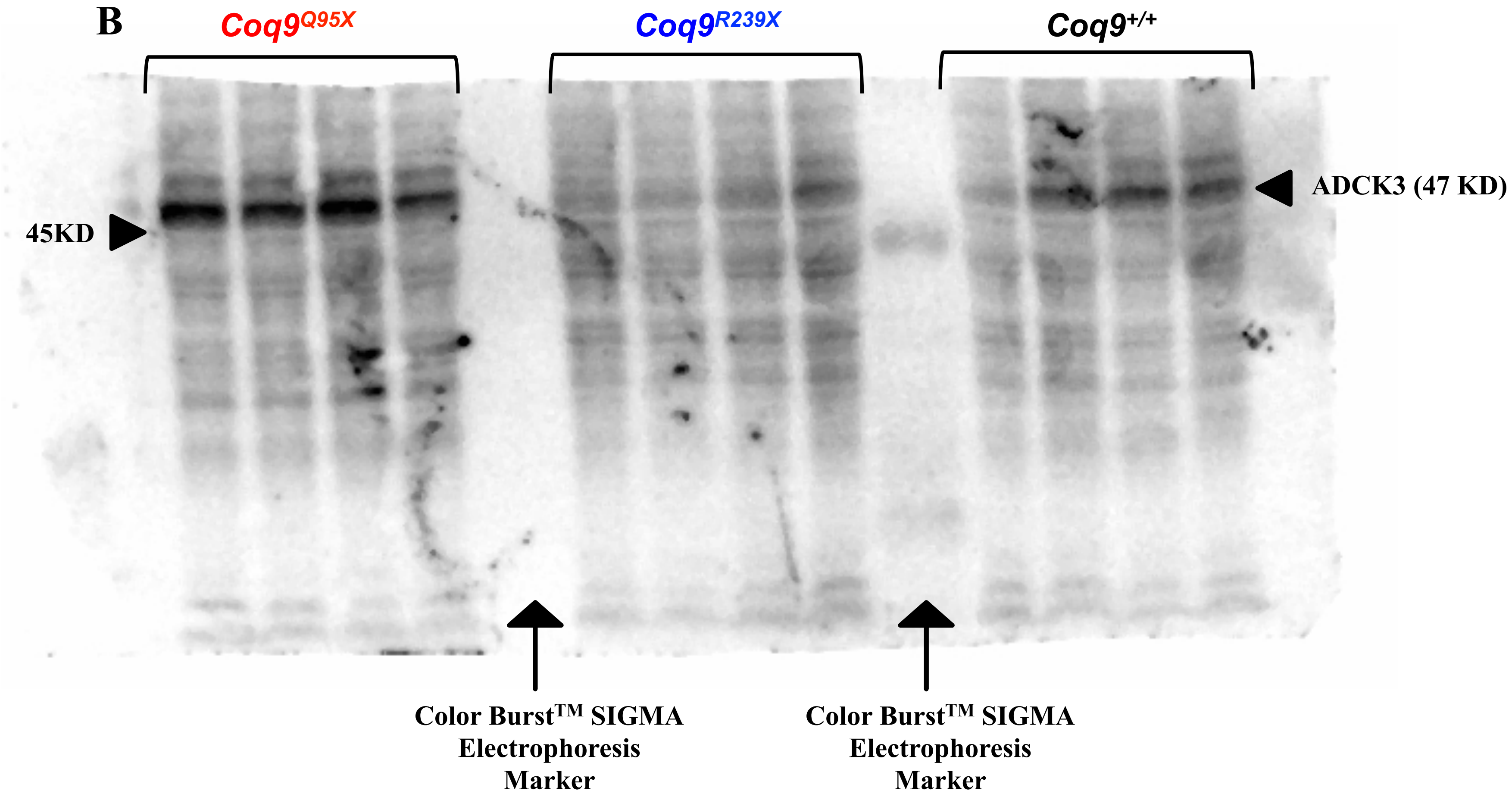

Figure 5. Panel C. Levels of Coq biosynthetic proteins

(C) Kidney western blot of COQ5.

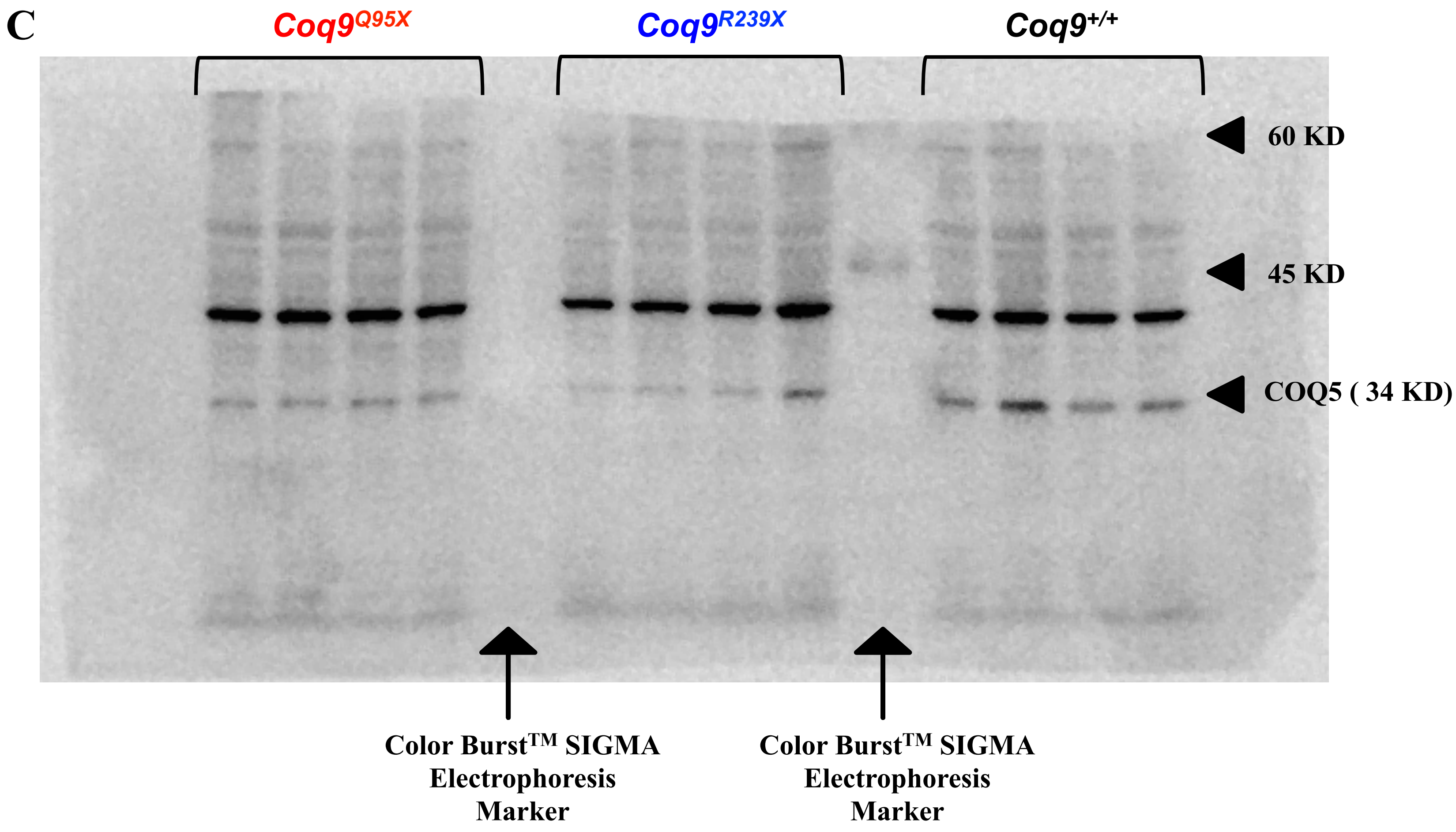

Figure 5. Panel D. Levels of Coq biosynthetic proteins

(D) Kidney western blot of COQ6.

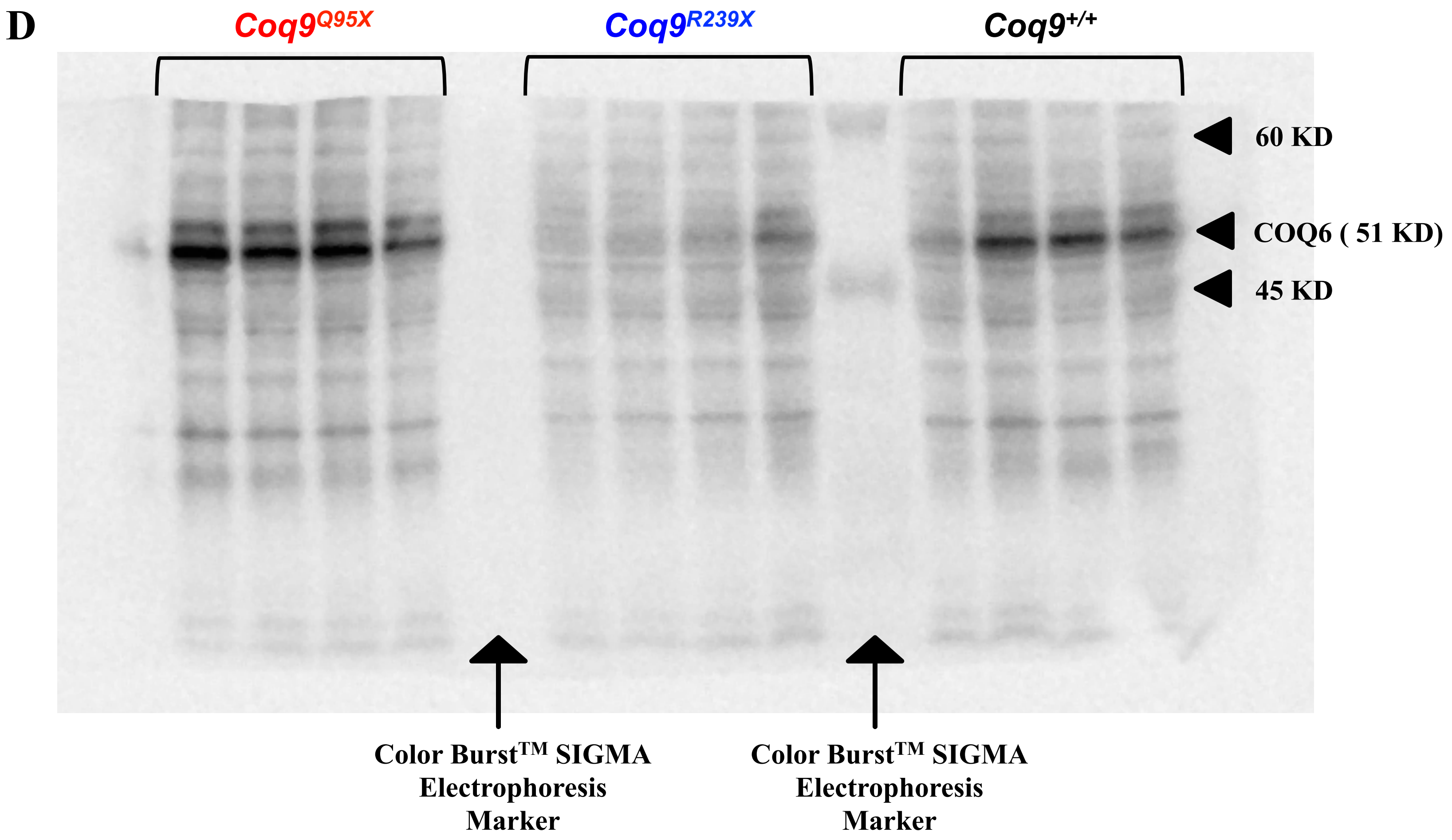

Figure 5. Panel E. Levels of Coq biosynthetic proteins

(E) T. surae western blot of COQ7.

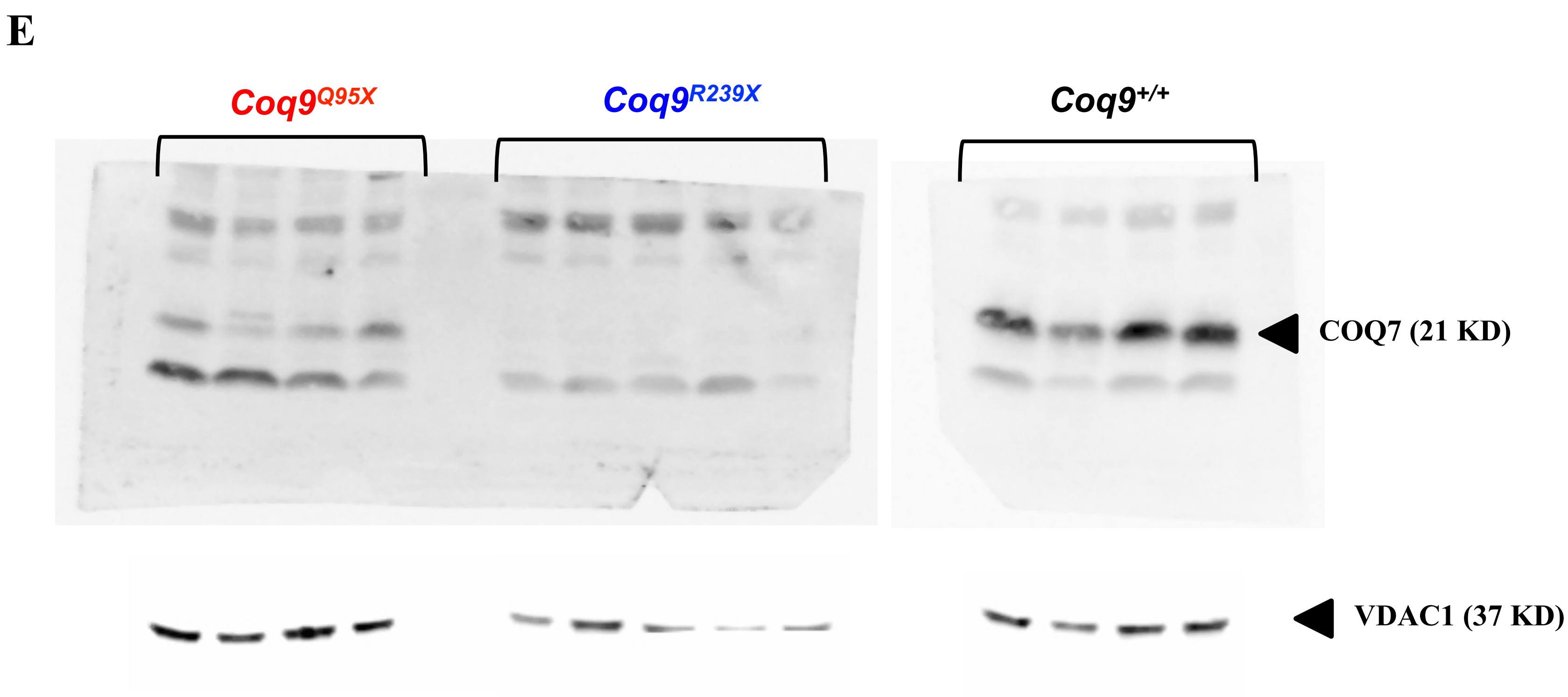

Figure 5. Panel F. Levels of Coq biosynthetic proteins

(F) T. surae western blot of ADCK3.

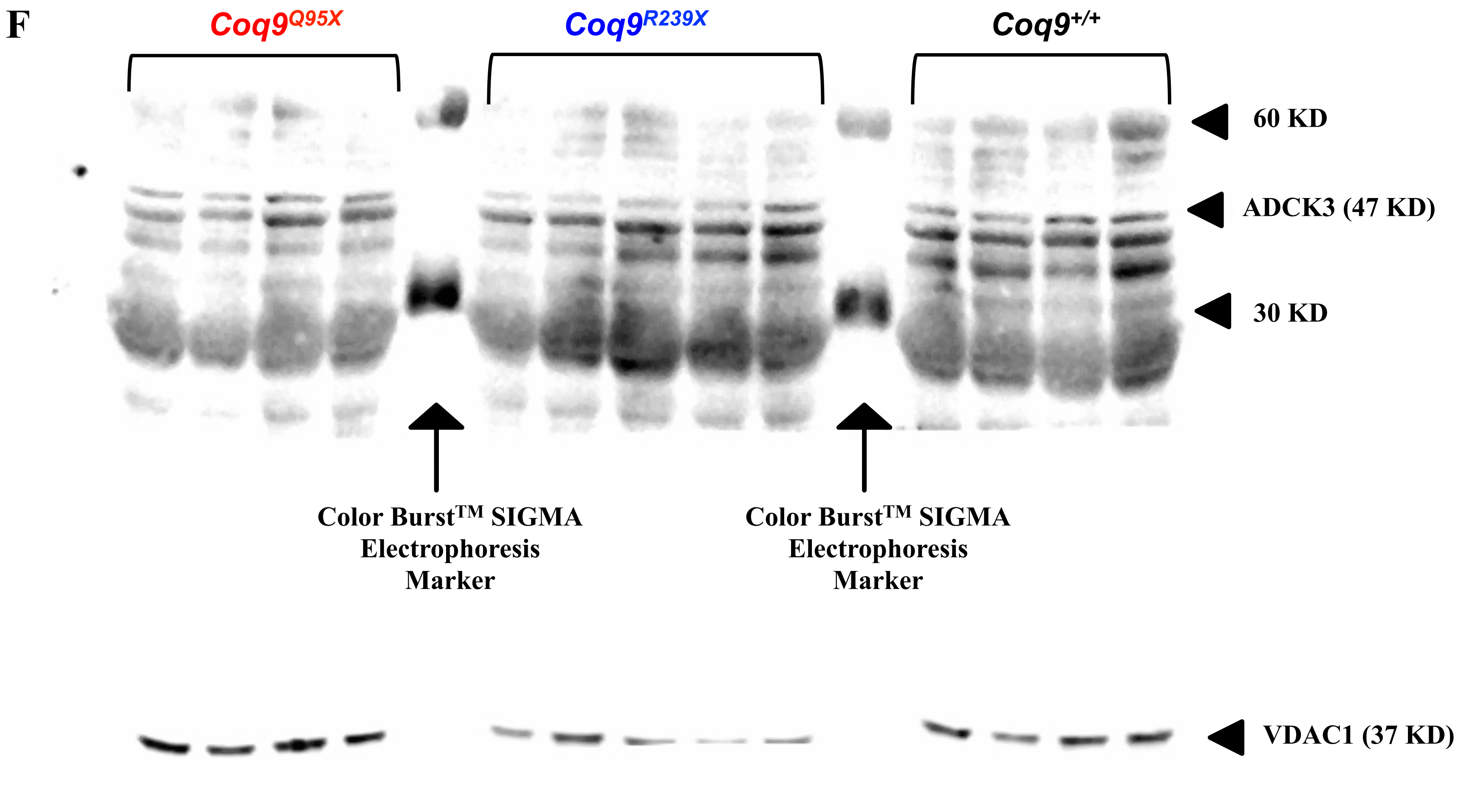

Figure 5. Panel G. Levels of Coq biosynthetic proteins

(G) T. surae western blot of COQ5.

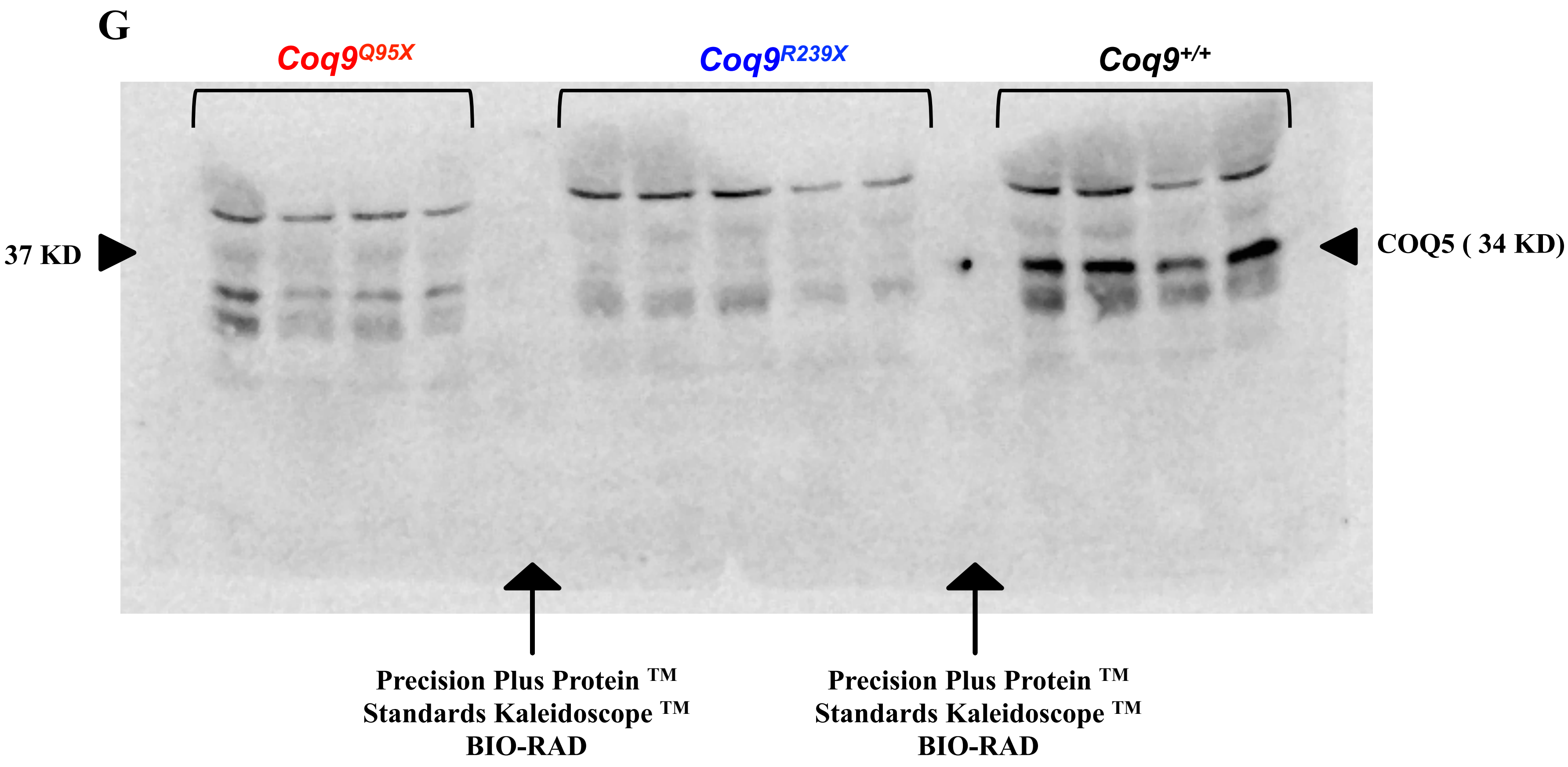

Figure 5. Panel H. Levels of Coq biosynthetic proteins

(H) T. surae western blot of COQ6.

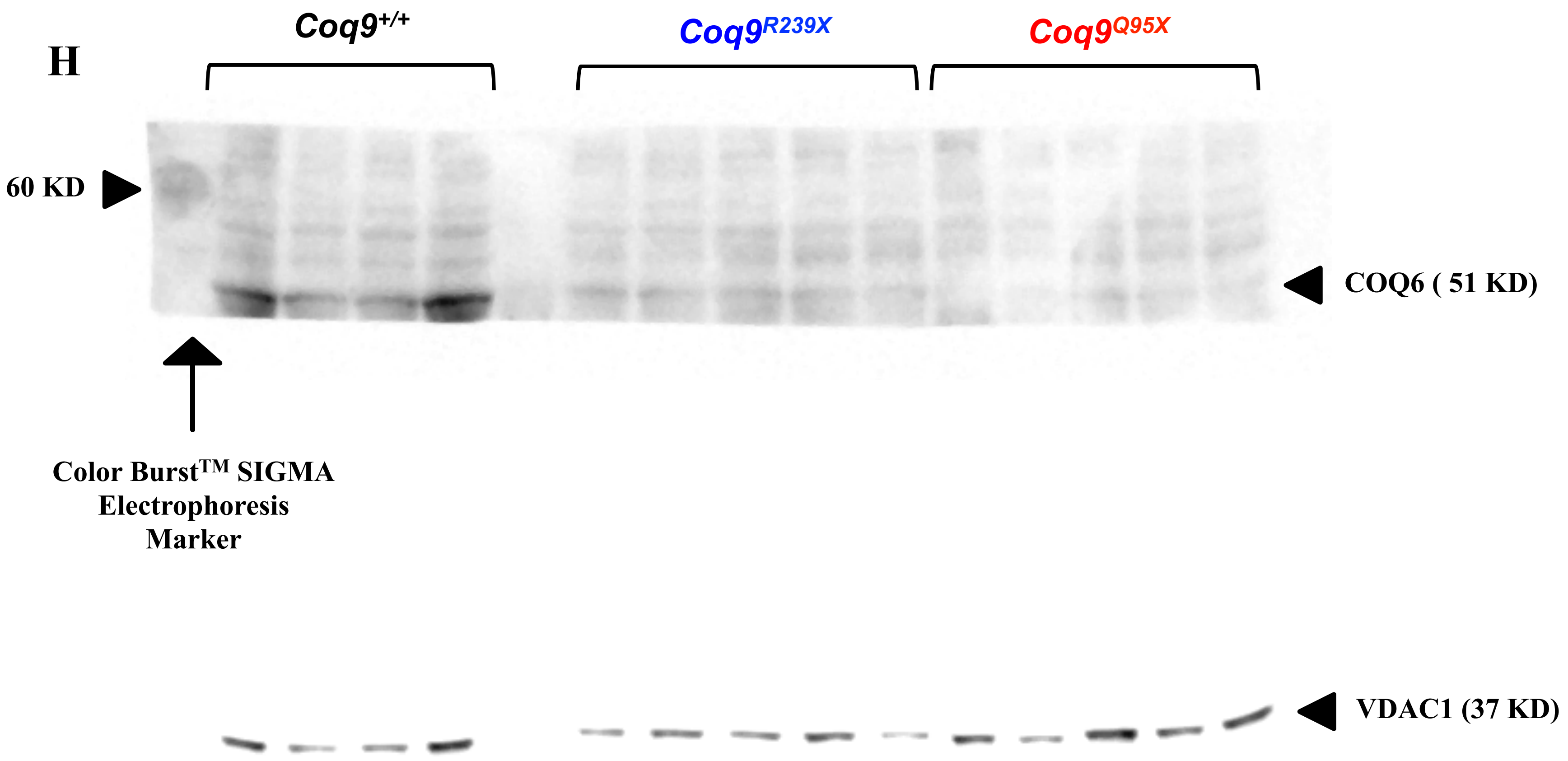

Supplement: Supplementary file 19 [file emmm0007-0670-sd19.pdf]
