# Supplementary material for: The clinical heterogeneity of coenzyme Q10 deficiency results from genotypic differences in the Coq9 gene
Source: EMBO Mol Med. 2015 Mar 23;7(5):670–87. doi: 10.15252/emmm.201404632 (PMC4492823; doi:10.15252/emmm.201404632)

Figure 6. Panel L. Blue-native gel electrophoresis (BNGE) of mitochondrial supercomplexes .

(L) Muscle (BNGE) of mitochondrial supercomplexes

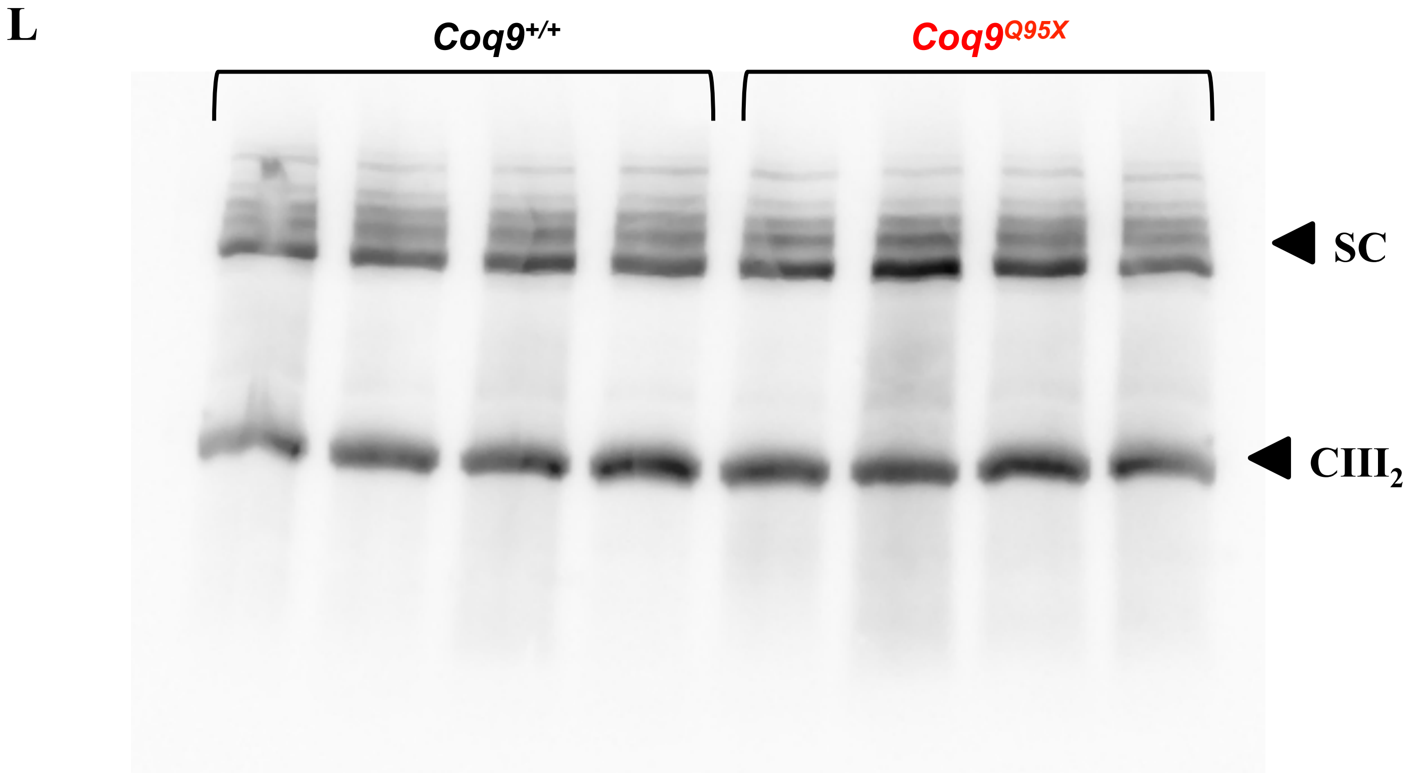

Supplement: Supplementary file 20 [file emmm0007-0670-sd20.pdf]
